# A Pilot, Randomised, Placebo-Controlled, Double-Blind Trial of a Single Oral Dose of Ivermectin for Post-Exposure Prophylaxis of SARS-CoV-2

**DOI:** 10.3390/pharmaceutics17091205

**Published:** 2025-09-16

**Authors:** Kylie M. Wagstaff, Mark S. Stein, Alan Herschtal, Jean-Jacques Rajter, Juliana Cepelowicz Rajter, Michele Sallaberger, Alexia Smileski, Amala Kanagalingam, David A. Jans

**Affiliations:** 1 Biomedicine Discovery Institute, Monash University, Clayton, VIC 3800, Australia; david.jans@monash.edu; 2 Knox Private Hospital, Wantirna, VIC 3152, Australia; 3 Holmesglen Private Hospital, Moorabbin, VIC 3189, Australia; 4 School of Public Health and Preventative Medicine, Monash University, Melbourne, VIC 3004, Australia; alan.herschtal@monash.edu; 5 Pulmonary and Sleep Consultants, 1001 South Andrews Ave, Fort Lauderdale, FL 33316, USA; jjr@pscflorida.com (J.-J.R.);; 6 Neuroscience Trials Australia, 30 Royal Parade, Parkville, VIC 3052, Australia; michele.sallaberger@florey.edu.au (M.S.); amala.kanagalingam@florey.edu.au (A.K.)

**Keywords:** ivermectin, post-exposure prophylaxis, SARS-CoV-2, randomised controlled trial

## Abstract

**Background:** The efficacy of a single oral dose of Ivermectin as prophylaxis for SARS-CoV-2 is uncertain. This trial sought to evaluate the effectiveness of a single oral low dose of Ivermectin to prevent SARS-CoV-2 infection or reduce symptoms if infection did occur. **Methods:** Asymptomatic community-dwelling adults were enrolled in this study within 72 h of close contact with a case of SARS-CoV-2. Participants were randomised, stratified by vaccination status and exposure site, to a single oral 200 µg/kg dose of Ivermectin or placebo. The primary outcome was conversion to a positive polymerase chain reaction (PCR) or rapid antigen test (RAT) for SARS-CoV-2 within 14 days of close contact. Secondary outcomes were restricted to those who met the primary outcome. They included the following: days alive free of symptoms in the 14 (DAFS1-14) and 28 (DAFS1-28) days following intervention and days from close contact until a positive PCR or RAT for SARS-CoV-2. **Results:** A total of 536 participants registered for this trial. Of these, 86 met inclusion criteria and were randomised. 68 adhered to the trial protocol and were included in the analysis. A total of 11/36 (Ivermectin arm) and 11/32 (placebo arm) met the primary outcome. After controlling for age and prior SARS-CoV-2 infection, the estimate (95% confidence interval (95% CI)) of the effect of Ivermectin (compared to placebo) on the absolute value of the proportion of participants converting to a positive PCR or RAT was −0.051 (−0.26 to 0.16), *p* = 0.63. After controlling for prior SARS-CoV-2 infection, age, body mass index, hypertension and lung disease, the average treatment effect (Ivermectin versus placebo) on DAFS1-14 was 2.5 days (95%CI 1.1 to 4.5), *p* = 0.036, and for DAFS1-28, was 2.3 days (95% CI 0.7 to 3.3), *p* = 0.35. The mean (standard deviation) number of days from close contact until a positive PCR or RAT was 5.0 (4.1) days for the Ivermectin group versus 2.6 (0.8) days for the placebo group. After controlling for age and prior SARS-CoV-2 infection, the average treatment effect (95%CI), Ivermectin versus placebo, on days from close contact until a positive PCR or RAT was 2.3 days (95% CI 1.1 to 3.4), *p* = 0.033. **Conclusions:** We did not demonstrate that a single oral low dose of Ivermectin administered to asymptomatic adults within 72 h of close contact with a case of SARS-CoV-2 prevents conversion to a positive PCR or RAT. However, the trial had a small sample size and does not exclude a clinically meaningful effect of Ivermectin on conversion to a positive PCR or RAT. Amongst those who did convert to a positive PCR or RAT, the use of Ivermectin significantly lengthened the time from close contact to conversion and increased the number of days alive free of symptoms following intervention.

## 1. Introduction

Within 6 years of its discovery in 1975, Ivermectin (a macrocyclic lactone mixture of 22,23-dihydroavermectin B1a and B1b) was employed successfully for parasitic infections in animals, and then it was approved for human use against onchocerciasis (river blindness) in 1987 [1,2,3,4]. It has since been used successfully to treat filariasis, strongyloidiasis/ascariasis, ectoparasites causing scabies, pediculosis and rosacea [2,3,4,5,6]. The work of Campbell and Omura in developing Ivermectin as a novel therapeutic against “infections caused by roundworm parasites” was recognised with the Nobel Prize in 2015 [2].

Ivermectin is usually administered as a single annual oral dose (of 150 or 200 µg/kg) to treat, respectively, onchocerciasis and strongyloidiasis in humans, with filariasis similarly treated with an annual dose (300–400 µg/kg) in endemic areas or alternatively twice yearly (150–200 µg/kg) [7]. An analysis of the first 11 years of mass global Ivermectin administration indicated a very low incidence of serious adverse side effects [6,8], with no instance of resistance reported in over 25 years. Acute administration to healthy 18–45-year-old subjects across a range of doses is well tolerated [7], while doses up to 2000 µg/kg without tolerability concerns have been reported in patients with parasitic infections [9]. However, there may be concerns in the elderly or in cases where the blood–brain barrier may be compromised [10,11,12,13,14,15,16,17,18] or in the setting of intercurrent medication which may affect Ivermectin’s systemic or CNS concentration (see [12], including Suppl. Appendix). Tolerability has not been formally examined in children below 5 years old/those under 15 kg/pregnant women/the elderly.

Multiple reports from in vitro infectious models document Ivermectin’s antiviral properties, including against flaviviruses such as dengue virus (DENV) and respiratory viruses such as SARS-CoV-2 [3,4,6,19,20,21,22,23,24,25,26,27,28,29,30,31]. Distinct from Ivermectin’s effects on helminthic chloride channels [32], Ivermectin’s broad-spectrum antiviral activity appears to be based on its ability to bind to and inhibit the nuclear transport roles of the host importin α (IMPα) protein [25,31,33], which is known to mediate the nuclear import of various viral proteins and key host factors. The effect of Ivermectin on immune function at the level of the whole organism is likely to be complex, with various ex vivo and in vivo studies highlighting both stimulatory and suppressive outcomes [34,35,36,37,38], while other quite distinct antiviral actions of Ivermectin have been proposed [26,39,40,41].

The results for a phase III human clinical trial in Thailand for DENV infection indicated that daily dosing (400 µg/kg) for 3 days reduced DENV nonstructural protein 1 antigenemia [42]. Human lung tissue pharmacokinetic (PK) data for Ivermectin derived directly from sampling beyond blood has not been reported, with modelling approaches based on human/animal tissue data yielding varying results. PK modelling based on the levels of Ivermectin achievable in human serum from standard 200 µg/kg dosing and robust measurement in large animal experiments indicates that concentrations of Ivermectin 10 times higher than the c. 2.5 μM EC_50_ for SARS-CoV-2 indicated by in vitro experiments [20] are likely achievable in the human lung [43]. Modelling based on different assumptions predicts lower values [44] but highlights the long-term stability of Ivermectin in the lung (>30 days in cattle) [45].

At the time of initiation of this trial in late 2021, the SARS-CoV-2 pandemic encompassed more than 80 million confirmed infections and close to 2 million deaths, with a number of clinical trials in progress for the treatment or prevention of SARS-CoV-2 (see [4]). The rationale for testing Ivermectin in this trial was based on the strong clinical retrospective association of statistically significantly lower mortality in patients who received oral Ivermectin (200 μg/kg) compared with usual care reported in the ICON study, a 280-patient propensity-matched cohort study at Broward Health Medical Centre (Florida, U.S.A) [46]. The mortality rate of 15% amongst subjects receiving Ivermectin (n = 173) was significantly lower compared with the 25.2% mortality in those not receiving it (n = 107), with the difference being more pronounced for the 75 subjects with severe pulmonary disease (mortality rates of 38.8% versus 80.7%). Additionally, in a retrospective study from Bangladesh, a single 12 mg dose of Ivermectin (which for a 60 kg patient is 200 μg/kg) given within 24 h of admission was associated with lower mortality from SARS-CoV-2 [47]. Meta-analyses [48,49] also reported data in keeping with benefit from Ivermectin prophylaxis, and randomised controlled trials had reported that Ivermectin reduced nasopharyngeal SARS-CoV-2 load [50] or reduced the duration of specific respiratory symptoms, concomitant with evidence for lower viral loads [39].

The prophylactic potential of a single oral dose of Ivermectin at 200 μg/kg to prevent transmission or decrease the symptoms of SARS-CoV-2, if given immediately post-exposure to a close contact, has not been previously examined. Although prophylactic trials involving Ivermectin have been performed previously (see [48,49,51,52,53]), none of these have been placebo-controlled rather than open-label, or used a single rather than repeated dose of Ivermectin; in addition, the open-label study of Shouman et al. [52] used doses higher than 200 μg/kg, whilst that of Chahla et al. [53] focused on health workers and used multiple weekly doses approximating 200 μg/kg Ivermectin in conjunction with 6 times daily iota-carrageen nasal spray. Our current trial is thus the first to test a single low dose of Ivermectin (200 μg/kg), using a randomised, double-blind, placebo-controlled methodology and recruiting community-dwelling adults to exclude participants whose exposure to a case of SARS-CoV-2 occurred in a hospital.

Here we evaluate the effectiveness of a single oral dose of 200 μg/kg Ivermectin, given to asymptomatic, rapid antigen test (RAT)- or polymerase chain reaction (PCR)-negative adults within 72 h of close contact exposure to a case of SARS-CoV-2, compared to placebo, to prevent transmission or reduce symptoms if transmission does occur. The results show that a single oral low dose of Ivermectin administered to asymptomatic, polymerase chain reaction (PCR)- or rapid antigen test (RAT)-negative participants within 72 h after close contact exposure to a case of SARS-CoV-2 did not prevent conversion to a positive PCR or RAT. However, amongst those who did convert to a positive PCR or RAT, the use of Ivermectin significantly lengthened the time to conversion and increased the number of days alive free of symptoms.

## 2. Materials and Methods

### 2.1. Trial Design and Setting

This fully remote, randomised, double-blind, placebo-controlled clinical trial investigating the potential of a single oral dose (200 μg/kg) of Ivermectin for post-exposure prophylaxis of SARS-CoV-2 was conducted from 22nd November 2021 to 31st May 2024 in the community of metropolitan (Melbourne) and regional (Ballarat and Bendigo) Victoria, Australia. The trial protocol was approved by Bellberry Human Research Ethics Committee (HREC) and prospectively registered with a trial registry (www.anzctr.org.au registration number—ACTRN12621001535864).

### 2.2. Participants

The trial was publicised through print and electronic media advertising and letter box flyer drops. Potential participants self-identified by submitting a registration via a trial website, which posted key inclusion and exclusion criteria. Once a potential participant had registered, they were contacted remotely via telephone by the staff of the contract research organisation (CRO), Neurosciences Trials Australia (NTA, Parkville, Victoria), and screened according to the inclusion/exclusion criteria, including self-reported weight and other self-reported information including sex, age, medical history, concomitant medications and symptoms.

#### 2.2.1. Inclusion Criteria

**Figure 1:**
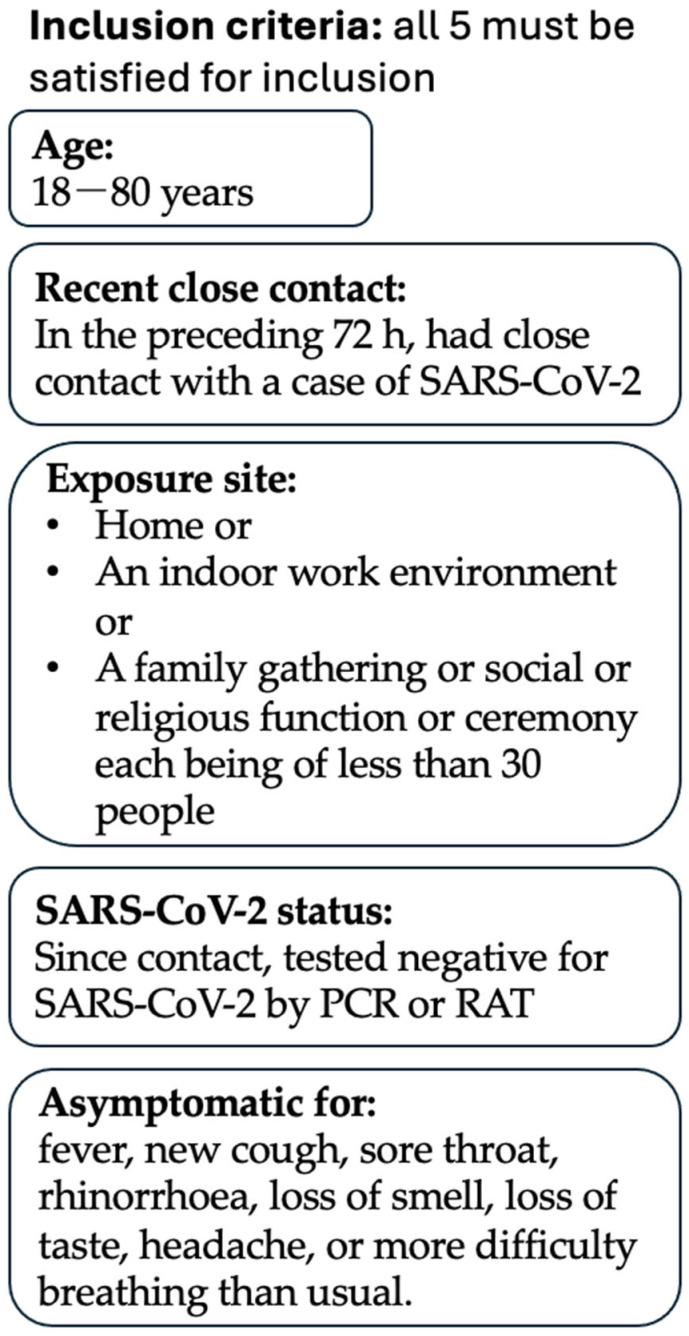
**Inclusion Criteria**. This trial’s inclusion criteria are summarised. A participant must satisfy all 5 inclusion criteria to be considered for this trial.

Inclusion criteria were as follows: aged between 18 and 80 years; AND in the preceding 72 h, had close contact with a case of SARS-CoV-2; AND this contact was in the context of (i) a home, (ii) an indoor work environment or (iii) a family gathering or social or religious function or ceremony, each comprising less than 30 people; AND since that contact, tested negative for SARS-CoV-2 by RAT or PCR; AND was asymptomatic of fever, new cough, sore throat, rhinorrhoea, loss of smell, loss of taste, headache or more difficulty breathing than usual. Inclusion criteria are summarised in Figure 1.

#### 2.2.2. Exclusion Criteria

Criteria for exclusion included the following: residing outside the recruitment catchment areas (greater Melbourne, Ballarat or Bendigo, Victoria); unable to provide contact information for a general practitioner/primary care physician with whom they had attended a consultation in the preceding 12 months; living alone; history of SARS-CoV-2 infection in the preceding 6 weeks or use of Ivermectin in the preceding 5 weeks; pregnancy or breastfeeding; and known allergies or contraindications to Ivermectin (see Supplementary Material S1 for full inclusion/exclusion criteria). Prior vaccination against SARS-CoV-2 was not an exclusion but was a randomisation stratum (see Section 2.3).

Those deemed eligible were forwarded the participant information and consent form electronically, given the public health circumstances. A trial consenting doctor discussed the trial by phone with those thereafter still wishing to participate, reviewed their inclusion and exclusion criteria and obtained informed verbal consent.

### 2.3. Randomisation

Participants were randomised to Ivermectin or placebo 1:1 by the respective regional clinical trial pharmacist using the trial REDCap (Vanderbuilt University, Nashville, TN, USA) database. Randomisation was conducted via concealed permuted blocks, stratified first by vaccination against SARS-CoV-2 and then by site of close contact exposure. In particular, vaccination status was categorised as follows: unvaccinated; received the first vaccination; received the second vaccination in the last 10 calendar days; received the second vaccination more than 10 calendar days ago but less than 6 months prior to consent; received the second vaccination 6 or more months prior to consent; received the third vaccination within the last 10 days; received the third vaccination more than 10 days ago; received the fourth vaccination within the last 10 days; received the fourth vaccination more than 10 days ago; received the fifth vaccination within the last 10 days; received the fifth vaccination more than 10 days ago; received the sixth vaccination within the last 10 days; received the sixth vaccination more than 10 days ago. If a participant satisfied more than one category, that participant was allotted to the last category in the above list which that participant fulfilled. Exposure site was categorised as follows: a home; an indoor work environment; a family gathering or a social or religious function or ceremony, each comprising fewer than 30 people.

### 2.4. Investigational Product (IP) and Intervention

Ivermectin for the trial was supplied in the form of 3 mg tablets (Edenbridge Pharmaceuticals, Parsipppany, NJ, USA), for which there were identical matched placebo tablets. Ivermectin was dispensed as follows to achieve a single oral dose of ~200 μg/kg: weight 45–50 kg, 3 tablets (9 mg); 51–65 kg, 4 tablets (12 mg); 66–79 kg, 5 tablets (15 mg); 80–91 kg, 6 tablets (18 mg); 92–105 kg, 7 tablets (21 mg) and 106–120 kg, 8 tablets (24 mg). Participants < 45 kg or >120 kg were excluded from the trial. Participants randomised to placebo received the same number of placebo tablets according to their respective weights (“as if’’ they had been randomised to Ivermectin; see Table S1, Supplementary Material S3).

Ivermectin or placebo tablets, trial RATs (see below) and pregnancy test kits (see below) were distributed to each participant from the nearest of three clinical trial pharmacies immediately following dispensing via contactless direct courier. Proof of delivery was ascertained from the courier company electronically by the CRO.

When the trial was established, government public health recommendations required Australians to use Australian Therapeutic Goods Administration (TGA)-approved RAT kits to perform a RAT on day six post-close contact. There were a variety of such kits available, and their supply varied from time to time during the trial. At trial establishment, however, a scarcity of TGA-approved RAT kits for detecting SARS-CoV-2 in Australia meant that insufficient numbers of TGA-approved RAT kits could be sourced for all the required RATs (see below).

Non-TGA-approved salivary RAT kits, included as part of the investigational product on the clinical trial notification (CTN) to the TGA, were accordingly supplied to the participants of the trial. The salivary RAT kits for SARS-CoV-2 were PCL COVID19 Ag Gold Saliva RATs (PCL Inc, Seoul, Republic of Korea). The kit product information (24 April 2023) reports a sensitivity of 94.44% for SARS-CoV-2 from a prospective study. This is comparable to available TGA-approved RAT kits.

Participants were instructed to perform a RAT on receipt of the RAT kits, prior to consuming the Ivermectin/placebo tablets and not to take trial Ivermectin/placebo tablets if the RAT was positive.

Female participants under 60 years of age were additionally dispensed a urine pregnancy test kit (1st Response instream, Church & Dwight, Sydney, Australia) to be used prior to taking the Ivermectin/placebo tablets. Participants were instructed not to consume their trial Ivermectin/placebo tablets if the pregnancy test was positive.

The negative status of the RAT and pregnancy tests and consumption of the Ivermectin/placebo tablets were confirmed by trial staff electronically from photographs of the test results and via telephone 4 h after confirmed delivery.

Participants were instructed that if the RAT and pregnancy test results were negative, they should take the trial Ivermectin/placebo tablets with water as soon as possible, at least 2 h after their last meal, and they should wait at least 1 h before eating their next meal.

Participants remained under the care of their primary care physicians who were all notified of participant enrolment by the trial consenting doctor immediately after participant consent was obtained (both over the phone and in a follow-up letter sent immediately afterwards by fax or email). The contact information of a trial senior doctor was also provided to both participants and their primary care physicians. No restriction was made on primary care physician management.

### 2.5. Follow-Up and Data Collection

Infection with SARS-CoV-2 was detected by a RAT or PCR in this trial. Sensitivity to detect infection was increased by frequent use of trial RATs whilst participants remained asymptomatic. For this purpose, trial RATs (see Section 2.4) were supplied for participants to be used immediately prior to the dose of Ivermectin/placebo and on days one, two, three, four thereafter and on day fourteen following their close contact (for that last test, day of close contact was counted as day zero).

Participants were asked to perform a TGA-approved RAT on day 6 following close contact exposure, in keeping with public health recommendations at the time. Participants obtained this RAT kit themselves. Participants were also asked to confirm any trial RAT-positive result with a TGA-approved RAT or with a PCR. Participants were encouraged to perform an additional RAT or PCR if they developed symptoms consistent with SARS-CoV-2 infection on other days and had not yet returned a positive RAT or PCR. The RAT results were verified by trial staff from photographs supplied by participants.

Participants were asked to complete a symptom diary for the first 28 days, starting on the day of consumption of their Ivermectin/placebo tablets, in which they specifically recorded the absence or presence of SARS-CoV-2-associated symptoms including: fever, new cough, sore throat, rhinorrhoea, loss of smell or taste, headache or more difficulty breathing than usual. They also noted any other symptoms and their severity. The questionnaire was submitted to the CRO electronically, weekly for the first four weeks following the consumption of their Ivermectin/placebo tablets.

CRO staff contacted each participant 4 h post-delivery of their Ivermectin/placebo tablets (for pre-Ivermectin/placebo tablet RAT and pregnancy test results, confirmation of tablet consumption and enquiry about any acute adverse reaction) and at days 8, 15, 22 and 29 (counting day 1 as the day of consumption of Ivermectin/placebo tablets for this purpose) and at months 2, 3, 4, 5 and 6. Data gathered electronically and/or by telephone included (for days 1–28) the following: RAT results, completed symptom questionnaires, concomitant medications, isolation status, adverse events and (months 2–6) mortality, hospitalisation and potential adverse events related to the Ivermectin/placebo tablets. See Supplementary Material S1 for full details of participant surveys and assessments.

### 2.6. Primary Endpoint

The primary endpoint of the trial was the difference between the Ivermectin and placebo arms in the proportion of participants who developed a positive RAT or PCR to SARS-CoV-2 within 14 days of close contact with a person infected with SARS-CoV-2.

### 2.7. Secondary Endpoints

Secondary endpoints were examined amongst those participants who returned a positive RAT or PCR to SARS-CoV-2 within 14 days of close contact. These endpoints included the difference between those who received Ivermectin versus placebo in terms of the following:

(i) Days alive free of SARS-CoV-2 symptoms (fever, new cough, sore throat, rhinorrhoea, loss of smell, loss of taste, headache, more difficulty breathing than usual) at day 14 (counting day of consumption of Ivermectin/placebo tablets as day 1 for this purpose). This endpoint is referred to as DAFS1-14.

(ii) Days alive free of SARS-CoV-2 symptoms (defined as above) at day 28. This endpoint is referred to as DAFS1-28.

(iii) Days alive free of presentation to hospital and/or acute hospital care and/or to outpatient care under hospital supervision at day 28 (counting day of consumption of Ivermectin/placebo tablets as day 1 for this purpose). Acute hospital care did not include days spent in an acute hospital ward solely because a rehabilitation or non-acute care facility bed was not available.

(iv) Time from close contact exposure with an index case of SARS CoV-2 to a positive TGA-approved RAT or PCR for SARS-CoV-2.

### 2.8. Statistics

A statistical analysis plan (SAP, see Supplementary Material S2) was prepared for the primary and secondary endpoints’ analyses and exploratory analyses. The SAP was lodged with both the trial HREC and the trial data safety monitoring board (DSMB) prior to data analyses.

#### 2.8.1. Primary Endpoint

The definitive analysis was conducted using a multivariate logistic regression model, constructed in R [54] with Firth’s bias adjustment method [55], with conversion status (defined as a positive result on TGA-approved RAT or PCR to SARS-CoV-2 within 14 days following close contact) as the dependent (response) variable and treatment arm (Ivermectin vs. placebo) as the independent variable. Confounding variables were pre-selected using prior clinical knowledge and following a blinded (to treatment allocation) analysis of the entire data set.

Sensitivity analyses of the primary endpoint were:

(i) A univariate logistic regression model, constructed as above but without controlling for any confounders.

(ii) A test of whether, amongst those who converted to a positive RAT or PCR to SARS-CoV-2 within 14 days following close contact, the proportion of Ivermectin-allocated cases differed (by the Wilson score method) significantly from 0.529, which was the overall proportion of participants who were randomly allocated to the Ivermectin arm.

#### 2.8.2. Secondary Endpoint

For DAFS1-14 and DAFS1-28, and days alive free of presentation to hospital and/or acute hospital care and/or to outpatient care under hospital supervision at day 28, the definitive analyses were multivariate beta-binomial regression models with the number of days as the response variable (dependent variable) and the treatment arm as the exposure variable (independent variable). Additional confounding variables pre-selected using prior clinical knowledge and following a blinded (to treatment allocation) analysis of the entire data set were incorporated as additional independent variables. For each of these secondary endpoints, a univariate version of each of the models was also produced, in which confounders were not controlled for. It was pre-determined that conclusions would be based on the high-dimensional (multivariate) models were they to converge and on the univariate model otherwise.

The secondary endpoint, the difference between treatment arms in time from close contact exposure to an index case of SARS CoV-2 until a positive TGA-approved RAT or PCR for SARS-CoV-2, was assessed using a negative binomial model, constructed with time until RAT or PCR positivity as the outcome variable and treatment group as the independent variable of interest, controlling for the same covariates as for the primary endpoint.

#### 2.8.3. Pre-Specified Exploratory Analyses

Several pre-specified exploratory analyses were also performed to describe the relationship between acquiring a positive RAT or PCR in the first 14 days following close contact and predictors of interest. These were performed using univariate logistic regression models with Firth’s correction. This was repeated using multivariate models controlling for treatment arm, days from close contact to IP administration and whether the participant had been infected with SARS-CoV-2 in the past. The exception was that in the modelling of the “early administration of IP” (defined as day 0 or 1 post-close contact), days from close contact to IP administration was not controlled for, due to likely collinearity between these two variables.

Pre-specified sensitivity analyses were also conducted on all trial analyses (primary and secondary) excluding participants whose administered Ivermectin dose was < 200 μg/kg (see Section 3 and Supplementary Material S3).

Statistical analyses were performed using the R environment for statistical programming, version 4.2.1 [54].

The full trial protocol and statistical analysis plan are available online (Supplementary Materials S1 and S2, respectively).

## 3. Results

### 3.1. Trial Population

**Figure 2:**
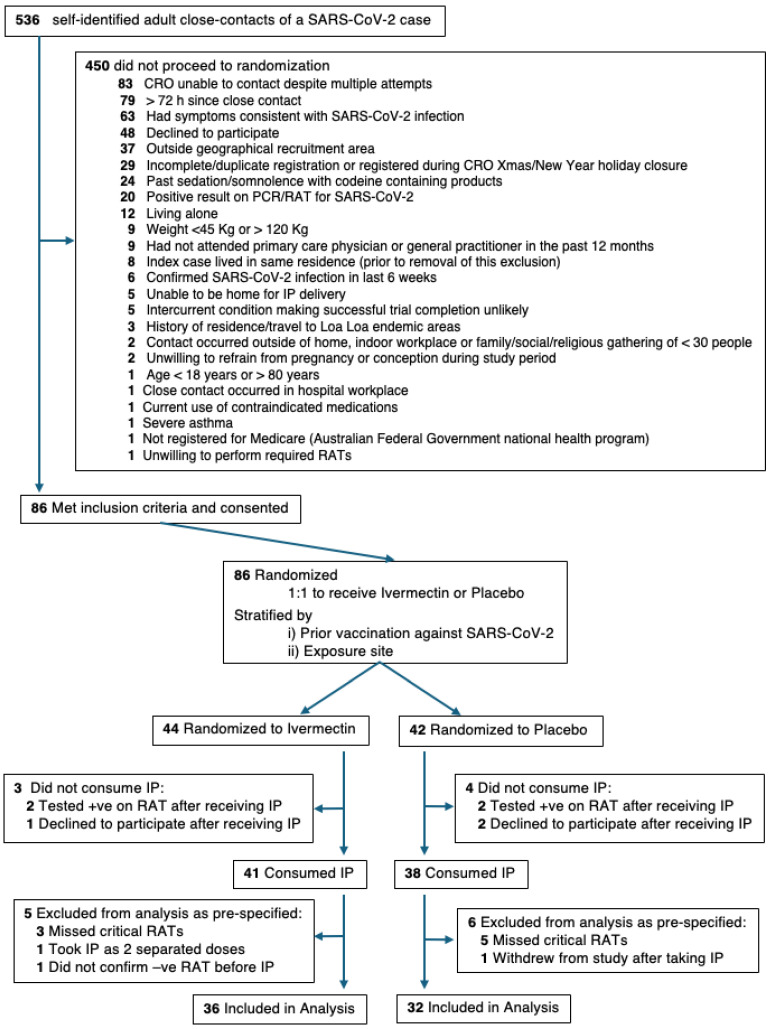
**A flow diagram of participants randomised and available for analysis in this pilot, randomised, double-blind, placebo-controlled clinical trial of Ivermectin for post-exposure prophylaxis of SARS-CoV-2.** The CONSORT diagram depicts participant flow from registration to analysis including the randomisation pathway and reasons for exclusion. All participant exclusions were actioned in a sequential fashion, as per the trial protocol, such that each participant was excluded for a single reason, even when multiple reasons may have existed.

A CONSORT diagram of the participants registered in this trial is shown in Figure 2. Recruitment was closed pragmatically due to the unexpected length of time recruiting for the trial and after reviewing power calculations. When the trial was approaching close of recruitment, power calculations based on blinded analysis using simulated data indicated that the trial had achieved approximately 80% power to detect an absolute difference between the treatment arms of 0.25 in the proportion of participants who convert to a positive RAT or PCR within 14 days of close contact and approximately 90% power to detect an absolute difference of 0.28. This was based on univariate logistic regression with Firth’s bias adjustment method with the dependent variable being conversion to a positive RAT or PCR and the independent variable being treatment allocation, with an alpha of 0.05. At this stage, 86 of the 536 potential participants who had registered for the trial met the eligibility criteria and had consented. They were randomised to Ivermectin (44 participants) or matched placebo (42 participants). Of these, seven did not take IP, because they tested positive with a trial RAT prior to taking IP (two Ivermectin, two placebo) or they withdrew consent (one Ivermectin, two placebo). Following IP, eight participants (three Ivermectin and five placebo) missed critical RATs as defined in the SAP (see Supplementary Material S2) and were excluded from analysis. A further three participants were excluded for protocol non-compliance (two Ivermectin) or due to a combination of non-compliance and the late withdrawal of consent (one placebo). This left 36 (Ivermectin) and 32 (placebo) participants available for analysis (Figure 2). Photo confirmation was received by the trial staff for all the RAT and PCR results except for one RAT which was performed over the CRO Christmas closure. Whilst the target Ivermectin dose was 200 µg/kg, the actual doses received were 180–235 µg/kg (see Table S1 in Supplementary Material S3).

The trial population included 43 women (63%) and 25 men (37%) with a median age of 50 (range 18 to 73) years and a median BMI (body mass index) of 26.4 (range 18.5 to 47.0) (Table 1). All participants reported having received at least two previous vaccinations against SARS-CoV-2 with 55 (81%) participants having received three or four and 8 (12%) having received five or six vaccinations. A total of 55 patients (81%) received their last vaccination >10 days but <6 months prior to enrolment. For their first two vaccinations, participants received vaccinations from Astra Zeneca (n = 30), Moderna (n = 1), Pfizer (n = 36) or company not recorded (n = 1). Participants were relatively healthy. A total of 91% were former or never smokers, and the median alcohol consumption was 20 g/week. There was a low prevalence of co-morbidities (Table 1).

At baseline, 34% of participants (23/68) reported isolating at home. 56% (38/68) were living with another close contact of a SARS-CoV-2 index case. 65% (44/68) were living with someone else positive for SARS-CoV-2. 34% (23/68) were living with both another close contact and with someone else positive for SARS-CoV-2. 87% (59/68) of participants were living with another close contact and/or an active case of SARS-CoV-2 (Table S2, Supplementary Material S3). 47% (32/68) of participants had a history of previous SARS-CoV-2 infection, but none had previously been hospitalised for SARS-CoV-2 infection (Table S3, Supplementary Material S3). Additional participant information, such as concomitant medication and vaccination status, compliance with RAT/PCR tests, completion of trial visits (including reasons for early termination) and reasons for exclusion from trial analysis, is provided in Tables S4–S7 (Supplementary Material S3).

The confounders selected for inclusion in the multivariate regression analysis for the primary endpoint were age at screening and whether the participant had SARS-CoV-2 at any time in the past.

### 3.2. Primary Endpoint

A total of 22 participants converted to a positive RAT or PCR within 14 days of close contact with a SARS-CoV-2-positive case. Of these, 11 received Ivermectin, and 11 received placebo. There was no significant difference in the proportion of participants who converted to a positive RAT or PCR between the Ivermectin and placebo arms, even after controlling for age and history of previous infection with SARS-CoV-2 (absolute difference = −0.051 ± 0.106; 95% CI: −0.26, 0.16; *p* = 0.632; Table 2). Sensitivity analyses led to the same conclusion (Table 2) when restricted to whether the actual dose of Ivermectin administered was at least 200 μg/kg (*p* = 0.769) or according to whether IP was administered early (on day 0 or 1) after close contact (*p* = 0.387).

### 3.3. Secondary Endpoints

The secondary endpoint analyses were restricted to the 22 participants who converted to a positive RAT or PCR for SARS-CoV-2. Symptom diaries were returned by 19. Participants who did not complete symptom diaries were excluded from the secondary analyses relating to symptoms (DAFS1-14 and DAFS1-28).

The mean number of days alive free of symptoms for days 1–14 (DAFS1-14), with day 1 being the day of IP consumption, was 7.2 (2.1) days (days (SD)) for Ivermectin and 5.2 (3.5) days for placebo. A multivariate analysis in which the covariates controlled for were past history of SARS-CoV-2 infection, age, BMI, hypertension and a history of lung disease revealed an average treatment effect of 2.5 more symptom-free days in favour of Ivermectin (95% confidence interval (CI) [1.1 to 4.5 days]; *p* = 0.036; Table 2). The estimated ratio of the odds of a symptom-free day in the Ivermectin group compared to the placebo group was 2.2. The equivalent model for DAFS1-28 yielded an average treatment effect of 2.3 days (95% CI [0.7 to 3.3 days], OR = 1.5]), but this did not reach significance (*p* = 0.35; Table 2). The average treatment effect is the difference between the model-based estimate of the number of days that a participant would be expected to be symptom-free if treated with Ivermectin and the model-based estimate of the number of days that a participant would be expected to be symptom-free if treated with placebo, averaged across all participants in the eligible set for this analysis.

The mean number of days alive and free of symptoms for each group and histograms of the number of days each participant experienced each symptom are given in Supplementary Material S3 (Table S10 and Figure S3, respectively). The use of antivirals by three participants (two who received placebo and one who received Ivermectin), given by their primary care physician, is examined in Table S11A–D, Supplementary Material S3.

No participant presented to a hospital for the care of SARS-CoV-2 in the first 28 days; hence no analysis of the secondary outcome of days alive free of presentation to hospital and/or acute hospital care and/or to outpatient care under hospital supervision at day 28 was performed.

**Figure 3:**
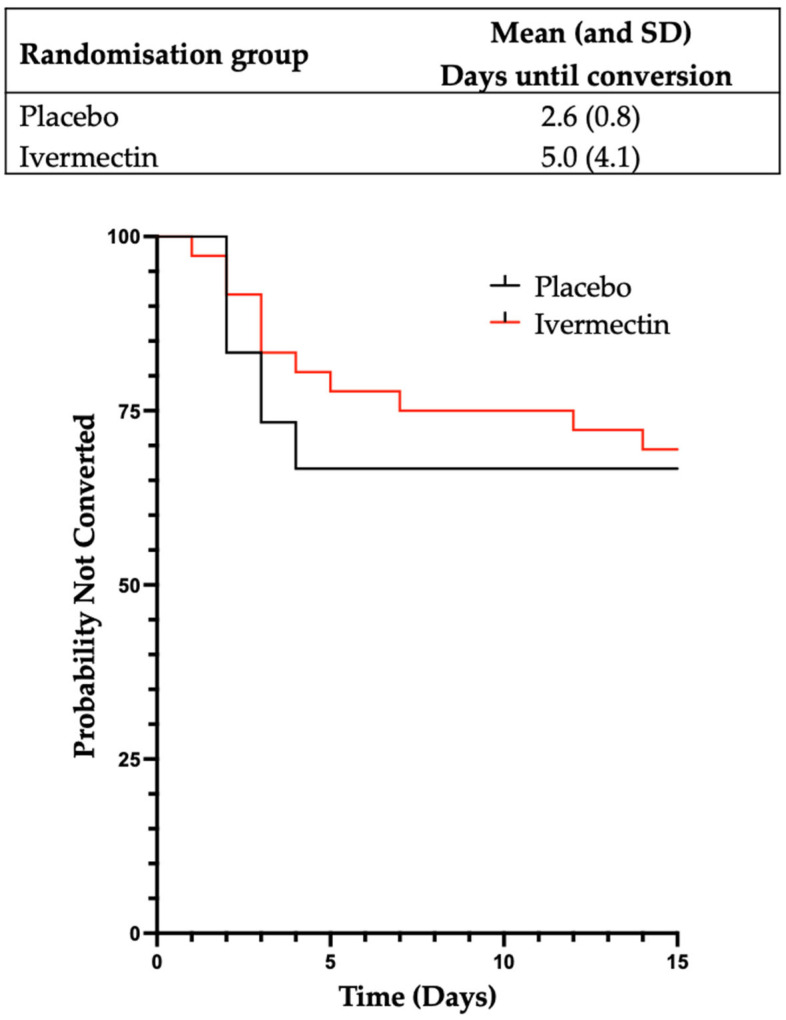
**A graphical representation of time from close contact until a positive PCR or RAT according to treatment arm**. Kaplan–Meier product limit curves for each treatment arm for the time in days from close contact until a positive PCR or RAT for SARS-CoV-2. Participants who did not convert to a positive test are considered to be censored after 14 days.

**Figure 4:**
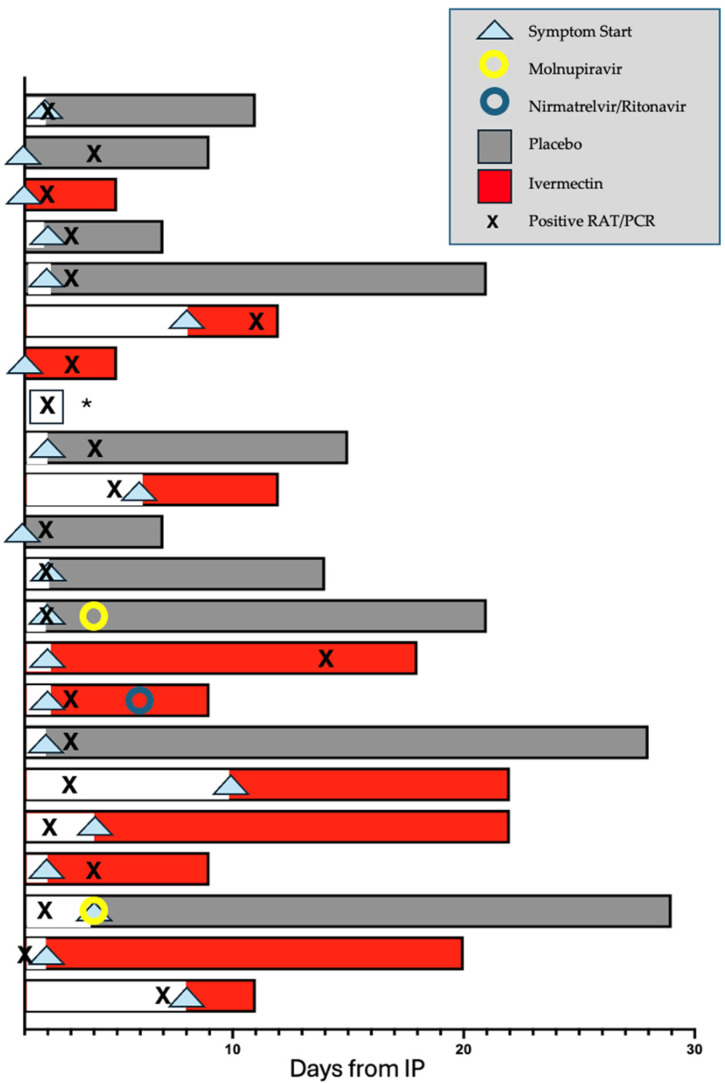
**Participant symptom timeline:** A swimmer plot showing the onset of participant symptoms (arrowhead), duration of symptoms (indicated by the shaded area of each bar), conversion to a positive PCR or RAT for SARS-CoV-2 (indicated by an X), treatment arm (Ivermectin in red and placebo in grey) and day of commencement of primary care physician-prescribed antiviral medication (nirmatrelvir/ritonavir, blue circle, or molnupiravir, yellow circle). Note that this figure focuses on the duration of symptoms after IP and plots days from day of IP (counted as day 1 for this purpose). This is distinct from the secondary endpoint of time to a positive PCR or RAT following close contact, which counts day 1 as days from close contact. * No detailed symptom siary provided was provided by this participant.

The effect of Ivermectin on the time from close contact with a case of SARS-CoV-2 to conversion to a positive RAT or PCR to SARS-CoV-2 was analysed (22 participants). A significant difference was observed, with participants in the Ivermectin group taking longer to convert to a positive PCR or RAT than those in the placebo group (mean (SD)) of 5.0 (4.1) days compared to 2.6 (0.8) days (Figure 3). In a multivariate model which adjusted for age and a history of prior infection with SARS-CoV-2, the average treatment effect was 2.3 days (95% CI [1.1 to 3.4 days]), with a rate ratio (the ratio of the mean time to a positive RAT or PCR in the Ivermectin participants compared to placebo participants) of 1.9 (*p* = 0.033). See Figure 4 for detailed participant timelines.

### 3.4. Exploratory Outcomes

The exploratory analyses describe the relationship between a positive PCR or RAT to SARS-CoV-2 within 14 days of close contact with a case of SARS-CoV-2 and potential predictors of interest (Table 2 and see Table S12, Supplementary Material S3). A history of past infection with SARS-CoV-2 was strongly negatively associated with conversion to a positive PCR or RAT for SARS-CoV-2 after close contact with a case of SARS-CoV-2, with an average treatment effect of a 30% reduction in risk of a positive PCR or RAT in both univariate (*p* = 0.003) and multivariate analyses (*p* = 0.002), Table 2. We did not observe an association between vaccine currency (defined as having received last vaccination against SARS-CoV-2 >10 days and <6 months ago) and conversion to a positive RAT or PCR following close contact. However, the numbers who did not have vaccine currency were small, being only 2 out of 22 (9%) who developed a positive PCR or RAT versus 4 out of 45 (9%) who did not (1 of whose vaccination details were not reported, so could not be included in the analysis). Additionally, no relationship was detected between age, use of ACE inhibitors/angiotensin II receptor blockers, BMI or the use of low-dose vitamin D (≤1000 IU/day) (Table 2) and conversion to a positive PCR or RAT following close contact. In contrast, days between close contact and IP administration was negatively associated with conversion to a positive PCR or RAT for SARS-CoV-2 in both univariate and multivariate analyses (Table 2). That is, the earlier administration of IP was associated with a higher likelihood of a positive PCR or RAT. This held true even in exploratory analyses restricted to the placebo arm (Table S13, Supplementary Material S3) and is considered further in Discussion.

### 3.5. Adverse Events

A number of adverse events were reported during the first 4 weeks of the trial, as detailed in Supplementary Material S3 Table S14A. These included two serious adverse events, one in each of the Ivermectin and placebo arms of this study. The events (vomiting/abdominal pain and musculoskeletal chest pain) resulted in hospitalisation of the participant. Both events were considered by a trial senior doctor and the DSMB, and ultimately, they were both considered unlikely to be related to the trial IP due to presentation and participant history. Adverse events were also reported in the 2–6-month participant follow-up period but no serious adverse events (see Supplementary Material S3 Table S14B).

## 4. Discussion

This pilot randomised controlled trial (RCT) tested a single oral low dose (200 μg/kg) of Ivermectin for post-exposure prophylaxis, following close contact with a case of SARS-CoV-2. All of the participants likely had a low viral load when they took Ivermectin, as they were all asymptomatic within 72 h of close contact and presented negative on a RAT or PCR immediately prior to treatment. In this trial, a single low dose of Ivermectin did not prevent conversion to a positive RAT or PCR within 14 days following close contact.

A limitation of this trial is the small sample size, with only 68 participants analysed and 22 conversions. As such, it does not exclude a clinically meaningful effect of Ivermectin on conversion to a positive PCR or RAT. In keeping with this, in interpreting the trial results, it is worth noting that the 95% confidence interval for the effect of Ivermectin on the absolute value of the proportion of participants who converted to a positive PCR or RAT for SARS-CoV-2 had a lower limit of –0.26. In future trials, larger sample sizes would offer greater power to detect an Ivermectin effect on conversion to a positive PCR or RAT.

For those who did convert to a positive RAT or PCR for SARS-CoV-2, the trial demonstrated that the use of Ivermectin lengthened the time from close contact to conversion (mean 5 days versus 2.6 days), and in the first 14 days following the consumption of Ivermectin/placebo tablets, it led to more days alive free of symptoms than placebo (average treatment effect of 2.5 days). A similar difference in days alive free of symptoms over the 1–28 days following the consumption of Ivermectin/placebo tablets is noted between those who received Ivermectin versus those who received placebo, although it did not reach significance.

The trial participants were at relatively low risk for significant SARS-CoV-2 disease. Although the median age was 50, participants were vaccinated and had a relatively low prevalence of heart disease, hypertension, lung disease or diabetes and had a median BMI in the mildly overweight rather than obese range. The trial thus demonstrated a clinically significant modification of disease by Ivermectin in a cohort of participants who had low clinical sensitivity to demonstrate an antiviral effect.

87% of the participants were isolating after their close contact in the same household as an active case of SARS-CoV-2 and/or another close contact of a case of SARS-CoV-2. Further exposure to SARS CoV-2, after exposure from the original close contact, could confound and reduce the trial sensitivity to detect an Ivermectin effect on the primary endpoint. If Ivermectin protects against infection from an index case but those randomised to Ivermectin become infected from a confounding secondary exposure thereafter, there should be a longer time to a positive PCR or RAT for SARS-CoV-2 amongst those who received Ivermectin, compared with those who received placebo. A longer (2.3 days) time to a positive RAT or PCR was indeed observed in those who received Ivermectin. It is thus possible that in some participants, Ivermectin did prevent conversion to a positive RAT or PCR after the initial close contact and that the longer mean time to a positive RAT or PCR observed amongst those positive cases who received Ivermectin reflected conversion following a subsequent exposure to SARS-CoV-2 after the single oral dose of Ivermectin had been metabolised or excreted. Such an explanation may be consistent with the data of the SAIVE trial (see below) which reported post-exposure prophylaxis efficacy from repeat Ivermectin dosing, although the isolation conditions/risk of secondary infection in that trial are not reported.

Another possible explanation is that Ivermectin did not prevent infection after close contact but, through direct or indirect mechanisms, altered the dynamics of viral replication so that the viral load needed to precipitate clinical disease (and a positive RAT or PCR) took longer to develop, and the subsequent disease course was shorter (2.5 more days alive free of symptoms). Both of these possibilities appear consistent with Ivermectin exhibiting a clinical antiviral effect against SARS-CoV-2.

Variability in the timing of Ivermectin administration over the span of a few days could have been a factor compromising trial sensitivity to detect early prophylactic efficacy. 37 of the 68 participants analysed (54%) received their Ivermectin or placebo on the day of, or the day following, their close contact with a case of SARS-CoV-2. When analysis was restricted to these 37 participants, the effect of Ivermectin to prevent SARS-CoV-2 infection remained not significant (Table 2), although the power of this analysis was also reduced by the smaller sample size.

This trial administered a single oral low dose of Ivermectin and did not demonstrate reduced conversion to a positive RAT or PCR, although the time to conversion was significantly lengthened. These results appear to be mostly consistent with a previous randomised, double-blind clinical trial in which 200 µg/kg Ivermectin followed by repeat daily 100 µg/kg Ivermectin doses was reported to be efficacious as post-exposure prophylaxis (SAIVE trial; NCT05305560; 2023; presented at the European Congress of Clinical Microbiology and Infectious Diseases, Copenhagen, 2023), in which a 71.5% reduction in conversion was observed in the Ivermectin group along with a significant reduction in symptoms. Thus, there appears to be merit in further RCTs testing different regimens of repeat low-dose Ivermectin for post-exposure prophylaxis of SARS-CoV-2.

It should be noted that the aforementioned SAIVE trial was conducted in an unvaccinated population. The trial here, by contrast, had a high vaccination rate. All participants had received at least two vaccinations against SARS-CoV-2. A total of 31 of the 68 participants (46%) had been vaccinated four or more times. It is possible that this high prevalence of vaccination may have impacted infection dynamics including viral load and limited the observed benefit of prophylaxis. In addition, many of the participants were recruited when omicron was a prevalent variant. Different variants may display different infection dynamics, and this could alter the observed benefit of prophylaxis.

Large sample size RCTs of Ivermectin to treat established clinical SARS-CoV-2 infection have not demonstrated definite clinical benefit. They have generally used higher doses than our post-exposure prophylaxis trial and often repeated these doses (to give an even higher cumulative dose). Ivermectin could have multiple direct or indirect effects on both SARS-CoV-2 and the host [20,34,35,36,37,38,39]. It is possible that at different doses, different effects promoting or not promoting clinical efficacy predominate. The effects of Ivermectin are also likely to vary according to whether treatment is given when viral load is low or in established disease, and all these variables merit further investigation.

Debate early in the pandemic considered whether a 200 μg/kg oral dose of Ivermectin would reach levels in human lung comparable to the Ivermectin concentrations found to have in vitro efficacy against SARS-CoV-2 [20,43,44]. As noted above in the Introduction, the decision here to test Ivermectin was based on retrospective clinical observations of efficacy of 200 µg/kg oral Ivermectin on mortality following infection with SARS-CoV-2 [46,47] rather than any in vitro observations. Ivermectin appears to impact many different pathways [4,31,39,40,41], and there are potentially more that are likely to be discovered, meaning that the consideration of a “relevant” tissue concentration for Ivermectin is complex to say the least. Clearly, the results for anti-viral efficacy from in vitro lung cell monolayer experiments are unlikely to provide full information with respect to the efficacious in vivo concentration required. The efficacious in vivo concentration may be much lower than that found to be efficacious in vitro because of synergism from other in vivo mechanisms (including innate and adaptive immune mechanisms) and/or potentially Ivermectin metabolites which are not reflected in the in vitro experimental model. The decades-long effective clinical use of drugs such as metformin and acetaminophen (paracetamol), in the absence of definitive knowledge of their mechanisms of action, emphasises that when there is a clinical observation, that alone could potentially serve as a rationale for a clinical trial, separate from PK/PD considerations stemming from in vitro work.

Epidemiological data are sparse regarding the expectation of conversion to a positive RAT or PCR following close contact. This trial therefore presents significant epidemiological findings. Most of our close contact instances occurred in the home; 11/32 (34%) of participants in the placebo arm converted to positive status, which likely mirrors the community clinical viral transmission rate from close contact in the home during the trial. The participants had been vaccinated against SARS-CoV-2. Currency of vaccination, defined as most recent vaccination more than 10 days but less than 6 months prior to enrolment, was not associated with the likelihood of conversion to a positive PCR or RAT, although the small numbers lacking such currency may have reduced our power to detect such an association. On the other hand, previous infection with SARS-CoV-2 was significantly associated with a reduced likelihood of conversion to a positive PCR or RAT. Most participants were recruited after the omicron variant became prevalent, and different findings may occur in trials conducted in the setting of other variants.

This trial also provides a template for the structure of a community-based RCT in the setting of a pandemic. The trial design enabled the screening and recruitment of participants; the dispensing and contactless drop-off of the investigational product, RAT kits and pregnancy tests; and remote data collection in a time-efficient manner while maintaining participant isolation. The trial sought to administer Ivermectin early after close contact and pragmatically selected a 72 h cut-off. In fact, half the participants (37/68, 54%) received their Ivermectin/placebo tablets on the same day or the day following close contact exposure, despite logistical restrictions associated with the pandemic.

Adequate vitamin D nutrition has been associated with a reduced likelihood of developing infection from SARS-CoV-2 [56,57,58]. ACE inhibitors and angiotensin II receptor blockers could lead to changes in the expression of the ACE2 receptor through which the SARS-CoV-2 spike protein may bind cells [59,60] and hence could affect the likelihood of developing infection following exposure to SARS-CoV-2. Exploratory analyses in this trial did not find an association between the use of low-dose vitamin D or the use of ACE inhibitors or angiotensin II receptor blockers and the likelihood of conversion to a positive PCR or RAT, but the power to detect such associations may be reduced by the small sample size. A limitation of this trial overall is its small number of participants.

Earlier administration of the investigational product following close contact was associated with a higher likelihood of a positive RAT or PCR for SARS-CoV-2 in the 14 days following close contact exposure. This was found even in exploratory analyses restricted to the placebo arm. This finding could occur if there was self-selection bias. In that case, participants with higher perceived exposure to SARS-CoV-2 may have acted faster. They may have been more motivated to rapidly register for and enrol in a post-exposure prophylaxis randomised clinical trial than participants who perceived that they were at a lesser risk of infection from their close contact exposure and hence may have taken longer to register for the trial.

In conclusion, in this pilot, randomised, placebo-controlled, double-blind trial, a single oral low dose of Ivermectin administered to asymptomatic, RAT- or PCR-negative participants within 72 h after close contact exposure to a case of SARS-CoV-2, did not prevent conversion to a positive RAT or PCR within 14 days of close contact. Amongst those who did convert to a positive RAT or PCR, the use of Ivermectin lengthened the time from close contact to conversion and increased the number of days alive free of symptoms in the 14 days following intervention.

## Figures and Tables

**Table 1:** Baseline characteristics *.

Demographic Characteristics	Ivermectin (n = 36)	Placebo (n = 32)	Overall (n = 68)
**Sex (%)**			
Female	23 (64%)	20 (63%)	43 (63%)
Male	13 (36%)	12 (38%)	25 (37%)
**Age (Years)**			
Mean (SD)	51.5 (11.5)	51.3 (12.4)	51.4 (11.8)
Median [Min, Max]	50.0 [24.0, 73.0]	50.0 [18.0, 71.0]	50.0 [18.0, 73.0]
**Weight (kg)**			
Mean (SD)	79.9 (15.7)	77.6 (14.5)	78.8 (15.1)
Median [Min, Max]	76.0 [57.0, 117]	77.0 [55.0, 113]	76.0 [55.0, 117]
**Self-Reported Height (cm)**			
Mean (SD)	169 (10.3)	170 (8.22)	170 (9.35)
Median [Min, Max]	169 [152, 195]	170 [155, 188]	169 [152, 195]
**BMI (kg/m^2^)**			
Mean (SD)	28.1 (5.77)	26.7 (4.11)	27.4 (5.07)
Median [Min, Max]	25.7 [18.5, 47.0]	27.1 [19.0, 36.5]	26.4 [18.5, 47.0]
**Vaccination against SARS-CoV-2 (highest vaccination status tabulated)**			
Received a second vaccination 6 or more months prior to consent	3 (8%)	2 (6%)	5 (7%)
Received a third vaccination within the last 10 days	3 (8%)	3 (9%)	6 (9%)
Received a third vaccination more than 10 days ago	9 (25%)	12 (38%)	21 (31%)
Received a fourth vaccination within the last 10 days	3 (8%)	2 (6%)	5 (7%)
Received a fourth vaccination more than 10 days ago	13 (36%)	10 (31%)	23 (34%)
Received a fifth vaccination within the last 10 days	1 (3%)	0	1 (2%)
Received a fifth vaccination more than 10 days ago	4 (11%)	2 (6%)	6 (9%)
Received a sixth vaccination within the last 10 days	0	1 (3%)	1 (2%)
**HISTORY**			
**Smoking status (%)**			
Never	23 (64%)	23 (72%)	46 (68%)
Former	10 (28%)	6 (19%)	16 (24%)
Current	3 (8%)	3 (9%)	6 (9%)
**Alcohol consumption per week (grams)**			
Mean (SD)	33 (43)	36 (54)	34 (48)
Median [Min, Max]	15 [0, 140]	20 [0, 210]	20 [0, 210]
**Diabetes (%)**			
No	33 (92%)	31 (97%)	64 (94%)
Yes	3 (8%)	1 (3%)	4 (6%)
**Heart disease (%)**			
No	36 (100%)	30 (94%)	66 (97%)
Yes	0	1 (3%)	1 (2%)
Missing	0 (0%)	1 (3%)	1 (2%)
**Lung disease (%)**			
No	32 (89%)	28 (88%)	60 (88%)
Yes	4 (11%)	4 (12.5%)	8 (12%)
**Hypertension (%)**			
No	26 (72%)	25 (78%)	51 (75%)
Yes	10 (28%)	7 (22%)	17 (25%)
**Kidney disease (%)**			
No	36 (100%)	31 (97%)	67 (99%)
Yes	0	1 (3 %)	1 (1.5%)
**Cancer (%)**			
No	31 (86%)	28 (88%)	59 (87%)
Yes	5 (14%)	4 (13%)	9 (13%)
**History of stroke (%)**			
No	36 (100%)	31 (97%)	67 (99%)
Missing	0 (0%)	1 (3%)	1 (2%)

* Percentages appearing not to sum to 100% are an artefact of rounding to 2 significant figures.

**Table 2:** Primary, Secondary and Exploratory Outcomes.

Outcome or Subgroup	Contrast	Model	Total (n)	Estimate ^a^	Std Error	95% CI	*p* Value
**Primary (Definitive)**							
Conversion to a SARS-CoV-2 +ve PCR or RAT	Ivermectin–Placebo	Multivariate ^b^	68	−0.051	0.106	[−0.26, 0.16]	0.632
**Primary (Sensitivity/Supplementary)**							
**Conversion to a SARS-CoV-2 +ve PCR or RAT:**							
Univariate Analysis	Ivermectin–Placebo	Univariate	68	−0.038	0.113	[−0.26, 0.18]	0.738
Proportion who received Ivermectin	Proportion that received Ivermectin	Proportion ^c^	22 (11 Ivermectin)	0.5 ^d^		[0.29, 0.71]	0.95
Actual Ivermectin dose ≥200 μg/kg ^e^	Ivermectin–Placebo	Multivariate ^b^	57	−0.033	0.113	[−0.25, 0.19]	0.769
IP administered on days 0–1 following close contact	Ivermectin–Placebo	Multivariate ^b^	37	−0.114	0.132	[−0.37, 0.14]	0.387
Proportion of placebo participants converting to a positive test, as a function of time from close contact until receipt of IP (Supplementary Analysis) ^f^	Per additional day	Multivariate ^b^	32	−0.176	0.079	[−0.33, −0.02]	0.026

	**Contrast**	**Model**	**Total** **(n)**	**ATE ^g^ (d)**	**OR/RR**	**95% CI**	***p* value**
**Secondary (Definitive)**							
Days Alive Free of Symptoms Days 1–14 ^h^ (definitive)	Ivermectin–Placebo	Multivariate beta-binomial ^i^	19	2.5	2.2 ^j^	[1.0, 4.5]	0.036
Days Alive Free of Symptoms Days 1–28 ^h^ (definitive)	Ivermectin–Placebo	Multivariate beta-binomial ^i^	19	2.3	1.5 ^j^	[0.7, 3.3]	0.350
Days from close contact until +ve PCR or RAT to SARS-CoV-2	Ivermectin–Placebo	Negative binomial ^k^	22	2.3	1.9 (RR ^l^)	[1.1, 3.4]	0.033
**Secondary (Sensitivity)**							
Days Alive Free of Symptoms Days 1–14 ^h^ (sensitivity)	Ivermectin–Placebo	Univariate beta-binomial	19	2.1	1.3 ^j^	[0.9, 3.8]	0.102
Days Alive Free of Symptoms Days 1–28 ^h^ (sensitivity)	Ivermectin–Placebo	Univariate beta-binomial	19	2.2	1.4 ^j^	[0.6, 3.4]	0.484

**Exploratory**		**Univariate ^m^**			**Multivariate ^m^**		
		**ATE^m^**	**95% CI**	***p* value**	**ATE ^m^**	**95% CI**	***p* value**
Age	Per year	0.006	[0, 0.02]	0.167	0.001	[−0.01, 0.01]	0.769
Days from close contact until IP administration	Per day	−0.163	[−0.26, −0.07]	0.001	−0.162	[−0.25, −0.07]	<0.001
IP administered on days 0–1 after close contact	No–Yes	0.225	[0.01, 0.44]	0.039	0.253	[0.06, 0.45]	0.01
History of past infection with SARS-CoV-2	No–Yes	−0.306	[−0.51, −0.1]	0.003	−0.302	[−0.5, −0.11]	0.002
Use of ACE inhibitor or angiotensin II receptor blocker	No–Yes	0.044	[−0.21, 0.3]	0.737	−0.018	[−0.24, 0.21]	0.878
BMI (kg/m^2^)	Per unit change in BMI	−0.005	[−0.03, 0.02]	0.621	−0.001	[−0.02, 0.02]	0.884
Taking low-dose vitamin D	No–Yes	−0.018	[−0.36, 0.32]	0.917	−0.066	[−0.34, 0.21]	0.634

Abbreviations: ATE, average treatment effect; IP, investigational product (Ivermectin/placebo tablets); OR, odds ratio; RR, rate ratio; Std Error, standard error; 95% CI, 95% confidence interval; +ve, positive. ^a^ Estimate of the absolute difference between the proportions of participants from each of the treatment arms who converted to a positive RAT or PCR to SARS-CoV-2 within 14 days of close contact with a case of SARS-CoV-2. ^b^ The model controlled for age at screening and whether the participant had infection with SARS-CoV-2 at any time in the past. ^c^ Participants were randomised 1:1 to receive either Ivermectin or placebo; however due to some participants being excluded (see Results), the percentage of participants in the sample eligible for analysis who received Ivermectin was not exactly 50%, but rather it was 52.9% (36/68). Thus, the proportion of participants who tested positive for SARS-CoV-2 who had received Ivermectin was tested against a null hypothesis of 52.9%. The 95% CI was calculated using the Wilson method. ^d^ Estimate of the proportion of participants who tested positive to SARS-CoV-2 who had been randomised to Ivermectin. ^e^ Ivermectin participants who received less than 200 µg/kg Ivermectin are excluded, and all placebo participants are included in this analysis. ^f^ Supplementary analysis, undertaken in the subgroup of participants who received placebo. The outcome variable is a positive RAT or PCR to SARS-CoV-2 within 14 days following close contact, and the predictor variable of interest is the time interval between close contact and receipt of placebo. See also Table S8, Supplementary Material S3. ^g^ The average treatment effect (ATE), which is the difference between the model-based estimate of the number of days that a participant would be expected to be symptom-free if treated with Ivermectin and the model-based estimate of the number of days that a participant would be expected to be symptom-free if treated with placebo, averaged across all participants in the eligible set for this analysis. ^h^ Only the subset of participants who converted to a positive RAT or PCR to SARS-CoV-2 within 14 days of close contact and completed their symptom questionnaires/diaries (n = 19) were included. For these analyses, day 1 is the day of consumption of IP. ^i^ The covariates controlled for in the multivariate beta-binomial models were as follows: past history of SARS-CoV-2 infection, age, BMI, hypertension and lung disease. ^j^ OR, odds ratio—the ratio of the odds of a symptom-free day in the Ivermectin group to the odds of a symptom-free day in the placebo group. ^k^ Negative binomial model with days from close contact with a case of SARS-CoV-2 until a positive RAT or PCR to SARS-CoV-2 as the outcome variable and treatment group as the independent variable of interest, controlling for the same covariates as for the primary endpoint. ^l^ The ‘rate ratio’ is the ratio of the mean time from close contact with a case of SARS-CoV-2 until a positive RAT or PCR to SARS-CoV-2 in the Ivermectin group to the mean time from close contact with a case of SARS-CoV-2 until a positive RAT or PCR to SARS-CoV-2 in the placebo group. A value >1 indicates a longer time from close contact with a case of SARS-CoV-2 until a positive RAT or PCR to SARS-CoV-2 in the Ivermectin group. ^m^ Exploratory outcomes were examined using univariate logistic regression models with Firth’s correction, with conversion to a positive RAT or PCR to SARS-CoV-2 within 14 days of close contact as the dependent variable (outcome variable) and the predictor of interest as the independent variable. The average treatment effect (ATE) in this case refers to the absolute change in the proportion of participants who converted to a positive RAT or PCR to SARS-CoV-2 as the predictor variable of interest increases one unit value (or goes from false to true). This exercise was repeated using multivariate models, in which the randomisation arm, days from close contact with a case of SARS-CoV-2 until IP administration and whether the participant had a history of SARS-CoV-2 infection were controlled for in all models. (The exception being that in the modelling of ‘IP administered on days 0–1′ (i.e., early administration of IP), ‘Days from close contact until IP administration’ was not controlled for due to likely collinearity between these two variables).

## Data Availability

Requests for de-identified participant-level data will be considered from recognised institutions only, taking into account HREC considerations.

## Supplementary Materials

The following supporting information can be downloaded at https://www.mdpi.com/article/10.3390/pharmaceutics17091205/s1, Supplementary Material S1 (Section 2.2.2 and Section 2.5); Supplementary Material S2 (Section 2.8 and Section 3.1) and Supplementary Material S3 (Section 2.4, Section 2.8.3, Section 3.1, Section 3.2, Section 3.3, Section 3.4, Section 3.5 and Section 4).

## Abbreviations

The following abbreviations are used in this manuscript:

ATE	Average Treatment Effect
BMI	Body Mass Index
CRO	Contract Research Organisation
DAFS	Days Alive Free of Symptoms
DSMB	Data Safety Monitoring Board
HREC	Human Research Ethics Committee
IP	Investigational Product
OR	Odds Ratio
RAT	Rapid Antigen Test
RCT	Randomised Controlled Trial
RR	Rate Ratio
SAP	Statistical Analysis Plan
