# Supplementary material for: A Pilot, Randomised, Placebo-Controlled, Double-Blind Trial of a Single Oral Dose of Ivermectin for Post-Exposure Prophylaxis of SARS-CoV-2
Source: Pharmaceutics. 2025 Sep 16;17(9):1205. doi: 10.3390/pharmaceutics17091205 (PMC12473430; doi:10.3390/pharmaceutics17091205)
Supplement: Supplementary file 1 [file pharmaceutics-17-01205-s001.zip › Pharmaceutics_3782031R1_Supplementary Material S3.pdf]

Supplementary Material S3

Supporting/Additional Tables and Figures

The following material provides further context and information related to the trial.

Table of Contents

Clinical Investigators 3

CRO (Neuroscience Trials Australia) 3

Senior Trial Doctors 3

Trial Consenting Doctors 3

Medical Monitors 3

Data Safety Monitoring Board (DSMB) 3

Clinical Pharmacy Sites and Staff 4

Medical Workforce Recruitment Agencies and Staff 4

Research Electronic Data Capture (REDCap) Management, Medical Director Prescribing Software and Trial Website staff 4

Statistician 4

Trial Pregnancy and Rapid Antigen Tests 4

S1. Number of Tablets of Ivermectin per Participant 5

Table S1a. Potential Ivermectin dose dispensed. 5

Table S1b. Actual Ivermectin dose dispensed according to trial arm and for both arms combined. 5

S2. Isolation Status 6

Table S2. Distribution of outcomes according to isolation status at the time of entry into trial. 6

S3. History of past infection with SARS-CoV-2 7

Table S3. History of past infection with SARS-CoV-2. 7

S4. Concurrent Medications and SARS-CoV-2 Vaccination Status 8

Table S4. Relationship between Conversion to a positive PCR or RAT for SARS-CoV-2 and Concurrent Medications and SARS-CoV-2 Vaccination Status. 8

S5. PCR and RAT Tests Conducted by Participants 10

Table S5. PCR and RAT tests conducted by participants according to treatment arm and overall.[#] 10

S6. Extent Of Follow Up at Trial Data Collection Timepoints 11

Table S6A. Contact at trial data collection timepoints 11

Table S6B. Timepoints missed and relevant outcomes thereafter. 11

S7. Reasons for exclusion from analysis 12

Table S7. Reasons for exclusion from analysis after consuming Ivermectin/Placebo, due to missing critical RATs 12

S8. Cross-tabulation of Days from close contact until receipt of Ivermectin/Placebo against conversion to a positive PCR or RAT for SARS-CoV-2 amongst Placebo participants. 12

Table S8. Cross-tabulation of Days from close contact until receipt of Ivermectin/Placebo against conversion to a positive PCR or RAT for SARS-CoV-2 amongst Placebo participants 12

S9. Effect of Ivermectin on Days Alive Free of Symptoms restricted to those receiving Ivermectin/Placebo on the day of or the day following close contact 13

Table S9. Effect of Ivermectin on Days Alive Free of Symptoms restricted to those receiving Ivermectin/Placebo on the day of or the day following close contact (n=15) 13

S10. Mean and Standard Deviation of the Days Alive Free of Symptoms by Treatment and Time to Ivermectin/Placebo. 14

Table S10. Mean and Standard Deviation of the Symptom Free Days per Group 14

S11. Effect of Ivermectin on Days Alive Free of Symptoms Excluding Participants given Antiviral Medication by their Primary Care Physicians From The Day They Commenced Antiviral Medication. 15

Table S11A. Effect of Ivermectin on Days Alive Free of Symptoms (DAFS), counting day of Investigational Product (IP) as day 1, after excluding participants who received Nirmatrelvir/Ritonavir (1 Ivermectin and 0 placebo) or Molnupiravir (0 Ivermectin and 2 placebo). 15

Table S11B. Effect of Ivermectin on DAFS amongst Participants who received IP early. 15

Table S11C. Analysis of the DAFS per randomisation group according to timing of IP following close contact. 16

Table S11D. Number of Days Eligible for inclusion in DAFS analysis amongst participants who received IP early. 16

S12. Cross-tabulation of Exploratory Predictors. 17

Table S12. Relationship between a positive PCR or RAT within 14 days following close contact with a case of SARS-CoV-2 and exploratory predictors. 17

S13. Relationship between early receipt of IP (defined as on the day of or the day following close contact) and conversion to a positive PCR or RAT for SARS-CoV-2, stratified by treatment group. 18

Table S13. Relationship between early receipt of IP (defined as on the day of or the day following close contact) and conversion to a positive PCR or RAT for SARS-CoV-2, stratified by treatment group. 18

S14. Adverse events in the trial. 19

Table S14A. Adverse events in the trial reported within the first 4 weeks. 19

Table S14B. Adverse events in the trial reported for months 2-6. 20

Figure S1. Goodness of fit analysis of the logistic regression model for the primary endpoint. 22

Figure S2. Histogram of Actual Doses Of Ivermectin Received 23

Figure S3. Analysis of Days of Symptoms. 24

Supplementary Reference 25

Clinical Investigators

Clinical Investigators: Kylie M. Wagstaff, Mark S. Stein, David, A. Jans, Jean-Jacques Rajter, Juliana Cepelowicz Rajter.

Associate Clinical Investigator: Joseph Torresi

CRO (Neuroscience Trials Australia)

Michele Sallaberger, Amala Kanagalingam, Alexia Smileski, Natalie Zajakovski, Jacob Niarchos, Jenny Dong, Liahna Toy, Kathy Skoff

Senior Trial Doctors

Stuart Watson, Salvatore Fiorenza

These doctors were employed by the trial to be the first point of contact for participants or their primary care physicians with any clinical concerns or questions during the trial and to follow up (obtain more information and/or provide urgent medical advice) and categorise adverse events identified by the CRO.

Trial Consenting Doctors

Xirui Stephy Zhang, Ebenezer Ojo, Jan Casey, Rebecca Warren

and,
Ahmed AlSultan, Fabian Dade, Chris Fogarty, Soma Herath, Jennifer Hunter, Tim Taulke-Johnson, Stefanie Kalfas, Jaiti Kosla, Andrea Ling, Warren Ling, Bently Logan, David Mathison, Anita Nesarajah, Jenny Nguyen, Wendy Ochtman, Kathrin Rac, Luke Rea, Philip Rodionoff, Russell Shute, Chi So, Paul Taiwo, Maria Ventura-Reyes, Sonia Volante, Wendy Walsh, Stuart Watson, Rubing Wei, Moya Wood.

These doctors were employed by the trial and trained specifically for their role in the processes of: obtaining informed verbal consent, prescribing IP and notifying primary care physicians of the enrolment of each participant in the trial.

Medical Monitors

Associate Professor Karam Kostner, Dr Renjy Nelson, Associate Profesor Paul Griffin
These specialist physicians were the trial medical monitors. All had clinical trial and/or clinical infectious diseases expertise.

Data Safety Monitoring Board (DSMB)

Professor Leigh Blizzard, Dr Ali Trad, Professor Duncan Topliss
The DSMB included specialist expertise in statistics, infectious disease and clinical trials.

Clinical Pharmacy Sites and Staff

1. HealthSmart Pharmacy, Box Hill Hospital, 8 Arnold St, Box Hill Victoria, Australia 3128
   Anthony Hrysoudis, Janice Yu, Jho Tung

2. Arnold St Pharmacy, 150 Arnold St, Bendigo, Victoria, Australia 3550
   Chia Lim

3. Eureka Pharmacy, 14 Albert St, Ballarat Central, Ballarat, Victoria, Australia 3350
   Mohammad Osman

Medical Workforce Recruitment Agencies and Staff

Medical Recruitment Pty Ltd (Varsity Lakes, Queensland, Australia)
David Shaw and Tarin Thurston recruited the above Senior Trial Doctors and Trial Consenting Doctors.

Recruitment Consultant- from CTBC (Melbourne, Victoria, Australia)
Corrine Taylor provided expert advice to the trial on workforce hiring regulation.

Research Electronic Data Capture (REDCap) Management, Medical Director Prescribing Software and Trial Website staff

Li Chin Quang wrote the REDCap (Vanderbilt University, Nashville Tennessee) database and maintained its files.

Cassie King was the liaison from Medical Director (Sydney, NSW) for the trial prescribing software.

Richard Sinnott built and maintained the trial website used for potential participant registration.

Statistician

Sabine Braat, (University of Melbourne) wrote the concealed permuted block stratified randomization protocol.

Alan Herschtal (Monash University) was the statistician responsible for the statistical analysis plan (SAP) and analysis of the trial results.

Trial Pregnancy and Rapid Antigen Tests

Pregnancy Test
1st response instream, Church & Dwight (Australia), Level 2, 22 Rodborough Road, Frenchs Forest NSW 2086

Rapid Antigen Test
PCL COVID19 Ag Gold Saliva RAT tests, PCL Inc, # 701, 99, Digital-ro 9-gil, Geumcheon-gu, Seoul, 08510, Rep. of Korea

S1. Number of Tablets of Ivermectin per Participant

Table S1a. Potential Ivermectin dose dispensed.

Weight (kg)*	Number of Tablets	Dose (mg)	Minimum Dose (μg/kg)	Maximum Dose (μg/kg)
<45	Excluded from trial	-	-	-
45-50	3	9	180	200
51-65	4	12	185	235
66-79	5	15	190	227
80-91	6	18	198	225
92-105	7	21	200	228
106-120	8	24	200	226
>120	Excluded from trial	-	-	-

[+]The trial aimed to prescribe a single oral dose of Ivermectin of 200 μg/kg using 3 mg Ivermectin tablets. Actual dispensed doses ranged around 200 μg/kg as indicated (See also Figure S2).
*Rounded up to the nearest kg

Table S1b. Actual Ivermectin dose dispensed according to trial arm and for both arms combined.

	Ivermectin (n = 36)	Placebo* (n = 32)	Overall (n = 68)
**Dose (μg/kg)**			
Mean (SD)	208 (11.3)	204 (11.0)	206 (11.3)
Median [Min, Max]	210 [185, 226]	202 [185, 225]	205 [185, 226]
Missing (percent)[a]	0 (0%)	1 (3%)	1 (1.5%)
**Dose < 200 μg/kg[a]**			
No	27 (75.0%)	23 (72%)	50 (73.5%)
Yes	9 (25.0%)	8 (25.0%)	17 (25.0%)
Missing	0 (0%)	1 (3%)**	1 (1.5%)

*Placebo "dose" calculated as if each placebo tablet had 3 mg placebo and is given for comparison.
** Placebo was dispensed but the clinical trials pharmacist failed to manually record confirmation that the number of tablets dispensed matched the number determined by the REDCap database at time of randomization.
[a]Percentages may not sum to 100% as an artefact of rounding to one decimal place.

S2. Isolation Status

Table S2. Distribution of outcomes according to isolation status at the time of entry into trial.

Isolation Status	Conversion Negative* (n = 46)	Conversion Positive* (n = 22)	Overall (n = 68)
**Isolating at home**			
Yes	17 (37%)	6 (27%)	23 (34%)
No	29 (63%)	16 (73%)	45 (66%)
**Living with another close contact of a case of SARS-CoV-2**			
Yes	30 (65%)	8 (36%)	38 (56%)
No	16 (35%)	14 (64%)	30 (44%)
**Living with someone positive for SARS-CoV-2**			
Yes	29 (63%)	15 (68%)	44 (65%)
No	17 (37%)	7 (32%)	24 (35%)
**Living with another close contact of a case of SARS-CoV-2 and someone positive for SARS-CoV-2**			
Yes	20 (43%)	3 (14%)	23 (34%)
No	26 (57%)	19 (86%)	45 (66%)
**Living with another close contact of a case of SARS-CoV-2 and/or someone positive for SARS-CoV-2**			
Yes	39 (85%)	20 (91%)	59 (87%)
No	7 (15%)	2 (9%)	9 (13%)

* Conversion Negative means the participant did not convert to a positive PCR or RAT for SARS-CoV-2 within 14 days of close contact with a case of SARS-CoV-2. Conversion positive means that the participant did convert to a positive PCR or RAT for SARS-CoV-2 within 14 days of close contact with a case of SARS-CoV-2.

S3. History of past infection with SARS-CoV-2

Table S3. History of past infection with SARS-CoV-2.

Past Infection with SARS-CoV-2	Conversion Negative* (n = 46)	Conversion Positive* (n = 22)	Overall (n = 68)
**Has the participant ever had an infection with SARS-CoV-2?**			
No**	19 (41%)	17 (77%)	36 (53%)
Yes	27 (59%)	5 (23%)	32 (47%)
**If yes, how many times?**			
Once	24 (52%)	4 (18%)	28 (41%)
Twice	3 (7%)	1 (5%)	4 (6%)
Not Applicable	19 (41%)	17 (77%)	36 (53%)
**Has the participant ever been hospitalised for SARS-CoV-2?**			
No	27 (59%)	5 (23%)	32 (47%)
Not Applicable	19 (41%)	17 (77%)	36 (53%)

* Conversion Negative means the participant did not convert to a positive PCR or RAT for SARS-SoV-2 within 14 days of close contact with a case of SARS-CoV-2. Conversion positive means that the participant did convert to a positive PCR or RAT for SARS-CoV-2 within 14 days of close contact with a case of SARS-CoV-2.

**For participants for whom the answer to this question was "No", the answers to each of the remaining questions was 'Not Applicable'.

S4. Concurrent Medications and SARS-CoV-2 Vaccination Status

Table S4. Relationship between Conversion to a positive PCR or RAT for SARS-CoV-2 and Concurrent Medications and SARS-CoV-2 Vaccination Status.

**At time of recruitment:**	**Conversion Negative*** **(n = 46)**	**Conversion Positive*** **(n = 22)**	**Overall** **(n = 68)**
	n (%)	n (%)	n (%)
**Vitamin D**			
Not taking	37 (80%)	18 (82%)	55 (81%)
Taking	9 (20%)	4 (18%)	13 (19%)
**Low Dose Vitamin D**[a]			
Not taking	41 (89%)	20 (91%)	61 (90%)
Taking	5 (11%)	2 (9%)	7 (10%)
**ACE Inhibitor?**[b]			
Not taking	42 (91%)	21 (96%)	63 (93%)
Taking	4 (9%)	1 (5%)	5 (7%)
**Angiotensin II Receptor Blocker**[c]			
Not taking	39 (85%)	17 (77%)	56 (82%)
Taking	7 (15%)	5 (23%)	12 (18%)
**ACE Inhibitor or Angiotensin II Receptor Blocker**			
Not taking	35 (76%)	16 (73%)	51 (75%)
Taking	11 (24%)	6 (27%)	17 (25%)
**Inhaled Glucocorticoids**			
Not taking	44 (96%)	22 (100%)	66 (97%)
Taking	2 (4%)	0 (0%)	2 (3%)
**HMG CoA reductase inhibitors (statins)**			
Not taking	42 (91%)	20 (91%)	62 (91%)
Taking	4 (9%)	2 (9%)	6 (9%)
**Vaccination status**[d]			
Current	41 (89%)	20 (91%)	61 (90%)
Not Current	4 (9%)	2 (9%)	6 (9%)
Missing [e]	1 (2%)	0 (0%)	1 (2%)

*Conversion Negative means the participant did not convert to a positive PCR or RAT for SARS-SoV-2 within 14 days of close contact with a case of SARS-CoV-2. Conversion positive means that the participant did convert to a positive PCR or RAT for SARS-CoV-2 within 14 days of close contact with a case of SARS-CoV-2.

[a]Low Dose Vitamin D is defined as ≤ 1000 IU daily

[b]ACE Inhibitors were included whether taken as a separate medication or as one component within a combination medication tablet.

[c]Angiotensin II Receptor Blockers were included whether taken as a separate medication or as one component within a combination medication tablet.
[d]Vaccination status = current if the individual has had at least 2 vaccines and the most recent one was >10 days but <6 months prior to accrual.
[e]Missing means that this information was not provided or collected for this patient.
*Percentages do not always sum to 100 due to rounding to the nearest integer.

S5. PCR and RAT Tests Conducted by Participants

Table S5. PCR and RAT tests conducted by participants according to treatment arm and overall.#

	Ivermectin (n = 36)	Placebo (n = 32)	Overall (n = 68)
**Did participant have an additional RAT test, beyond those mandated by the trial protocol during week 1? n (%)**			
No	25 (70%*)	17 (53%)	42 (62%)
Yes	11 (31%)	15 (47%)	26 (38%)
**Did participant have an additional RAT test, beyond those mandated by the trial protocol during week 2? n (%)**			
No	27 (75%)	22 (69%)	49 (72%)
Yes	9 (25%)	10 (31%)	19 (28%)
**Did participant have an additional RAT test, beyond those mandated by the trial protocol during week 3? n (%)**			
No	27 (75%)	25 (78%)	52 (77%)
Yes	9 (25%)	7 (22%)	16 (24%)
**Did participant have an additional RAT test, beyond those mandated by the trial protocol during week 4? n (%)**			
No	28 (78%)	27 (84%)	55 (81%)
Yes	8 (22%)	5 (16%)	13 (19%)
**PCR test performed during week 1? n (%)**			
No	26 (72%)	20 (63%)	46 (68%)
Yes	10 (28%)	12 (38%)	22 (32%)
**PCR test performed during week 2? n (%)**			
No	32 (89%)	24 (75%)	56 (82%)
Yes	4 (11%)	8 (25%)	12 (18%)
**PCR test performed during week 3? n (%)**			
No	35 (97%)	32 (100%)	67 (99%)
Yes	1 (3%)	0 (0%)	1 (2%)
**PCR test performed during week 4? n (%)**			
No	35 (97%)	31 (97%)	66 (97%)
Yes	0 (0%)	1 (3%)	1 (2%)
Missing	1 (3%)	0 (0%)	1 (2%)
**RAT performed on Day 2**? n (%)**			
No	0 (0%)	1 (3%)	1 (2%)
Yes	36 (100%)	31 (97%)	67 (99%)
**RAT performed on Day 3**? n (%)**			
No	0 (0%)	3 (9%)	3 (4%)
Yes	36 (100%)	29 (91%)	65 (96%)
**RAT performed on Day 4**? n (%)**			
No	4 (11%)	8 (25%)	12 (18%)
Yes	32 (89%)	24 (75%)	56 (82%)
**RAT performed on Day 5**? n (%)**			
No	7 (20%)	8 (25%)	15 (22%)
Yes	29 (81%)	24 (75%)	53 (78%)
**RAT performed on Day 6 following close contact using a TGA-approved ? n (%)**			
No	10 (28%)	15 (47%)	25 (37%)
Yes	26 (72%)	17 (53%)	43 (63%)

# Participants were not required to perform a PCR or RAT on days 2,3,4,5 if they had previously returned a positive result. Similarly, PCR tests were encouraged but not required.

* Percentages do not always sum to 100 percent due to rounding to the nearest integer.

** Days following trial investigational product (Ivermectin/Placebo tablets; IP) with day of IP being day 1.

S6. Extent Of Follow Up at Trial Data Collection Timepoints

Table S6A. Contact at trial data collection timepoints

Contact	Overall (n = 68)
**Was the participant contacted at every data collection timepoint? n (%)**	
Yes	51 (75 %)
No	17 (25 %)
**Reason for non contact**	
Participant withdrew consent (early termination)	1 (2 %)
Other (see **Table S6B**)	16 (24%)

Table S6B. Timepoints missed and relevant outcomes thereafter.

Timepoints missed	Relevant outcomes	Numbers (%)
Day 8 and Month 3	Data collected at Day 15 and Month 4 respectively	4 (25 %)
Day 15*	Data collected on Day 29	2 (13 %)
Day 22*	Data collected on Day 29	3 (19 %)
Days 22, 29 and Month 2	Data collected at Month 3	1 (6 %)
Month 2+	Data collected at Month 3	1 (6 %)
Month 3	Data collected at Month 4	1 (6 %)
Months 4 and 5	Data collected at Month 6	1 (6 %)
Month 5	Data collected at Month 6	2 (13 %)
Month 5	Data collected at Month 6	1 (6 %)

*Data collection missed due to Xmas/New Year CRO shutdown

+Data collection missed due to participant being overseas

S7. Reasons for exclusion from analysis.

Table S7. Reasons for exclusion from analysis after consuming Ivermectin/Placebo, due to missing critical RATs

Reason(s) for analysis exclusion	Overall (n = 8)
Did not do Day 6 TGA-approved RAT	6 (3.5%)
Did not do Day 14 RAT	2 (1.2%)

S8. Cross-tabulation of Days from close contact until receipt of Ivermectin/Placebo against conversion to a positive PCR or RAT for SARS-CoV-2 amongst Placebo participants.

Table S8. Cross-tabulation of Days from close contact until receipt of Ivermectin/Placebo against conversion to a positive PCR or RAT for SARS-CoV-2 amongst Placebo participants

Days from close contact until receipt of IP*	Converted to a Positive PCR or RAT	
	No	Yes
0	1	0
1	8	9
2	10	2
3	2	0

*Day of close contact = Day 0

S9. Effect of Ivermectin on Days Alive Free of Symptoms restricted to those receiving Ivermectin/Placebo on the day of or the day following close contact

Table S9. Effect of Ivermectin on Days Alive Free of Symptoms restricted to those receiving Ivermectin/Placebo on the day of or the day following close contact (n=15)

Model Type	Outcome	Estimate	95% CI	p value	ATE*
Multivariate	Days alive free of symptoms, Days 1-14	2.069	[0.73, 5.9]	0.174	2.339
	Days alive free of symptoms, Days 1-28	1.720	[0.58, 5.06]	0.325	3.320
Univariate	Days alive free of symptoms, Days 1-14	1.671	[0.68, 4.08]	0.259	1.749
	Days alive free of symptoms, Days 1-28	1.498	[0.53, 4.23]	0.446	2.790

*The average treatment effect (ATE), which is the difference between the model-based estimate of the number of days that a participant would be expected to be symptom free if treated with Ivermectin, and the model-based estimate of the number of days that a participant would be expected to be symptom free if treated with Placebo, averaged across all participants in the eligible set for this analysis.

S10. Mean and Standard Deviation of the Days Alive Free of Symptoms by Treatment and Time to Ivermectin/Placebo.

Table S10. Mean and Standard Deviation of the Symptom Free Days per Group

Days from close contact until IP administration*	Randomisation group	Mean (SD) Days Alive Free of Symptoms	
		Days 1-14+	Days 1-28+
0-3	Ivermectin	7.20 (2.1)	17.2 (5.8)
	Placebo	5.22 (3.5)	15.2 (9.1)
0 or 1	Ivermectin	6.86 (2.4)	17.4 (5.1)
	Placebo	5.25 (3.8)	14.8 (9.6)

*Day of close contact = Day 0.

+Days after IP (trial investigational product: Ivermectin/Placebo) administration with Day 1 being the day of IP administration

S11. Effect of Ivermectin on Days Alive Free of Symptoms Excluding Participants given Antiviral Medication by their Primary Care Physicians From The Day They Commenced Antiviral Medication.

Three participants (two who received placebo and one who received Ivermectin), were given Nirmatrelvir/Ritonavir (Paxlovid, Pfizer; 150 mg Nirmatrelvir tablets co-packaged with 100 mg Ritonavir tablets), prescribed in Australia as 2 Nirmatrelvir and 1 Ritonavir tablet every 12 h for 5 days (1 Ivermectin arm), or Molnupiravir 200 mg (Lagevrio, Merck Sharpe and Dohme) prescribed in Australia as 4 tablets every 12 h for 5 days (2 Placebo arm) by their primary care physician. Generally recommended for high-risk patients, neither agent is known to significantly affect mild-moderate symptoms of SARS-CoV-2 in low-risk patients (see Supplementary [1]). Specific analysis examining the effect of Ivermectin when censoring or excluding these participants was performed (**Table S11A-D**).

Table S11A shows estimates of the ratio of the odds of a symptom free day in the Ivermectin group to the odds of a symptom free day in the Placebo group to be in favour of Ivermectin, but not significantly; likely consequent upon reduced power due to censoring from the time of commencement of antiviral medication within the small sample (n = 19). Also reported is the average treatment effect (ATE), which is the difference between the model-based estimate of the number of days that a participant would be expected to be symptom free if treated with Ivermectin, and the model-based estimate of the number of days that a participant would be expected to be symptom free if treated with Placebo, averaged across all participants in the eligible set for this analysis.

Table S11A. Effect of Ivermectin on Days Alive Free of Symptoms (DAFS), counting day of Investigational Product (IP) as day 1, after excluding participants who received Nirmatrelvir/Ritonavir (1 Ivermectin and 0 placebo) or Molnupiravir (0 Ivermectin and 2 placebo). *

Model Type	Outcome	Estimate	95% CI	p value	ATE
Multivariate	DAFS for Days 1-14	1.5	[0.8, 3.1]	0.228	1.5
	DAFS for Days 1-28	1.1	[0.5, 2.6]	0.754	0.8
Univariate	DAFS for Days 1-14	1.3	[0.7, 2.6]	0.416	1.0
	DAFS for Days 1-28	1.0	[0.4, 2.4]	0.968	0.1

*The sample used (n = 19) censors the three participants (two placebo and one Ivermectin), who were given Nirmatrelvir/Ritonavir (1 Ivermectin arm), or Molnupiravir (2 Placebo arm) from the day that they first took an anti-viral medication. The multivariate model was adjusted for: a history of past infection with SARS-CoV-2, age, BMI, hypertension and a history of lung disease. DAFS counts day of IP as Day 1.

Table S11B shows the results of the analysis in the subset of the participants (n = 15) who received IP early (on the day of, or the day following close contact, analogous to the analysis in **Table 2** for the primary objective). The average treatment effect is reported here also, as for the analysis in **Table S11A**.

Table S11B. Effect of Ivermectin on DAFS amongst Participants who received IP early.

*Model Type	Outcome	Estimate of the odds ratio	95% CI	p value	ATE
Multivariate**	DAFS for Days 1-14	1.2	[0.4, 3.5]	0.711	0.7
	DAFS for Days 1-28	1.3	[0.4, 4.0]	0.686	1.5
Univariate	DAFS for Days 1-14	1.1	[0.5, 2.6]	0.777	0.4
	DAFS for Days 1-28	1.1	[0.4, 2.9]	0.905	0.4

*Regression analysis censors the participants (see above) who took anti-viral medications from the day that they first took an anti-viral medication.

**The multivariate model was adjusted for: a history of past infection with SARS-CoV-2, age, BMI, hypertension and a history of lung disease. DAFS counts day of IP as Day 1.

Table S11C shows the mean days alive free of symptoms per randomisation group, considering all participants (regardless of how many days after close contact they received IP) and considering only those who received IP early (on the day of, or the day following close contact).

Table S11C. Analysis of the DAFS per randomisation group according to timing of IP following close contact. *

**Days from close contact to IP**	**Randomisation group**	**Mean (and SD) of DAFS**	
		**1-14**	**1-28**
0-3	Ivermectin	7.4 (2.1)	17.8 (5.8)
	Placebo	6.3 (3.3)	18.1 (8.0)
0 or 1	Ivermectin	7.2 (2.5)	18.3 (5.0)
	Placebo	6.5 (3.5)	18.0 (8.7)

* This analysis excludes the three participants (see above) who took antiviral medications altogether in order to keep the maximum number of symptom free days consistent (either 14 or 28) across all participants summarised. The sample size is n = 16 for the days 0-3 from close contact to IP and n = 12 for the days 0 or 1 from close contact to IP. Time from close contact to IP (left hand column) counts day of close contact as day 0. DAFS1-14 and DAFS1-28 (right hand columns) count day of IP as day 1 for their timelines. The multivariate model was adjusted for: a history of past infection with SARS-CoV-2, age, BMI, hypertension and a history of lung disease.

Table S11D shows the distribution of the number of days for which participants were eligible for inclusion in the analyses of those who received IP early (days 0-1 after close contact; **Tables S11b and S11c)**, split by randomization group, considering that some were only eligible for part of the time window due to commencing anti-viral medications, as above.

Table S11D. Number of Days Eligible for inclusion in DAFS analysis amongst participants who received IP early. *

	**Placebo (n = 8)**	**Ivermectin (n = 7)**	**Overall (n = 15)**
**For DAFS analysis Days 1-14**			
4 days	2 (25 %)	0 (0 %)	2 (13%)
6 days	0 (0 %)	1 (14%)	1 (7 %)
14 days	6 (75 %)	6 (86%)	12 (80 %)
**For DAFS analysis Days 1-28**			
4 days	2 (25 %)	0 (0 %)	2 (13 %)
6 days	0 (0%)	1 (14%)	1 (7 %)
28 days	6 (75 %)	6 (86%)	12 (80 %)

*2 participants who received placebo later received antiviral medication from their primary care physician. They both received Molnupiravir and both received it on day 5 following IP. The participant who received Ivermectin and later received antiviral medication received Nirmatrelvir/Ritonavir on day 7 following IP.

S12. Cross-tabulation of Exploratory Predictors.

Table S12. Relationship between a positive PCR or RAT within 14 days following close contact with a case of SARS-CoV-2 and exploratory predictors.

	Conversion to a positive PCR or RAT for SARS-CoV-2			
	**Conversion Negative (n = 46)**	**Conversion Positive (n = 22)**	**Overall (n = 68)**	**p value^**
**Age (years)**				
Mean (SD)	50 (11)	54 (12.6)	51 (11.8)	0.167
Median [Min, Max]	48.5 [18.0, 73.0]	55.5 [24.0, 70.0]	50.0 [18.0, 73.0]	
**Days From Close Contact Until IP Administration**				
Mean (SD)	1.7 (0.8)	1.2 (0.6)	1.5 (0.8)	0.001
Median [Min, Max]	2.0 [0, 3.0]	1.0 [0, 2.0]	1.0 [0, 3.0]	
Missing*	1 (2%)	0 (0%)	1 (2%)	
**Early Receipt of IP****				
No	24 (52%)	6 (27%)	30 (44%)	0.039
Yes	21 (46%)	16 (73%)	37 (54%)	
Missing*	1 (2%)	0 (0%)	1 (2%)	
**Has the participant had past infection with SARS-CoV-2?**				
No	19 (41%)	17 (77%)	36 (53%)	0.003
Yes	27 (59%)	5 (23%)	32 (47%)	
**Taking ACE Inhibitor or Angiotensin II Receptor Blocker?**				
No	35 (76%)	16 (73%)	51 (75%)	0.737
Yes	11 (24%)	6 (27%)	17 (25%)	
**BMI (kg/m^2)**				
Mean (SD)	27.7 (5.7)	26.9 (3.6)	27.4 (5.1)	0.621
Median [Min, Max]	26.7 [18.5, 47.0]	26.1 [21.0, 35.0]	26.4 [18.5, 47.0]	
**Taking Low Dose Vitamin D*****				
No	41 (89%)	20 (91%)	61 (90%)	0.917
Yes	5 (11%)	2 (9%)	7 (10%)	

^p value determined for the difference between Conversion Negative and Conversion Positive in the indicated row.
* Missing – The data was not recorded for the indicated participants.
**Early receipt of IP is defined as receiving IP on the day of, or the day following close contact
*** Low dose vitamin D was less than or equal to 1000 international units per day

S13. Relationship between early receipt of IP (defined as on the day of or the day following close contact) and conversion to a positive PCR or RAT for SARS-CoV-2, stratified by treatment group.

Table S13. Relationship between early receipt of IP (defined as on the day of or the day following close contact) and conversion to a positive PCR or RAT for SARS-CoV-2, stratified by treatment group.

	**Early receipt of placebo**		**Early receipt of Ivermectin**	
	**No (n = 14)**	**Yes (n = 18)**	**No (n = 16)**	**Yes (n = 19)**
Conversion Negative	12 (86%)	9 (50%)	12 (75%)	12 (63%)
Conversion Positive	2 (14%)	9 (50%)	4 (25%)	7 (37%)

S14. Adverse events in the trial.

Table S14A lists Adverse Events within the first 4 weeks following consumption of investigation product (IP) recorded amongst the 68 participants included in the analysis for the primary outcome. Symptoms consistent with infection by SARS-CoV-2 (fever, sore throat, rhinorrhoea, headache, loss of smell, loss of taste, new cough, more difficulty breathing than usual) have not been included. Adverse events were listed according to their timing following IP and according to the treatment allocation of the participant. Lines with the same alphabetical superscript occurred in the same participant.

Table S14A. Adverse events in the trial reported within the first 4 weeks.

Treatment Arm	Adverse Event	Relationship to IP*	Severity**	SAE***
**Occurred between consumption of IP and the 4-hour phone review**				
Placebo[a]	Nausea	Probably	Mild	
Placebo	Numb tip of tongue	Unlikely	Mild	
**Occurred during Week 1**				
Ivermectin[f]	Nausea	Probably related	Mild	
Ivermectin[f]	Constipation	Probably related	Mild	
Placebo[c]	Generalised hive-like rash	Related	Severe	
Placebo	Lower back pain	Not related	Moderate	
Placebo	Insomnia	Not related	Moderate	
Placebo	Lightheaded and dizziness upon bending down with mild elevation of heart rate.	Unlikely	Mild	
Placebo	Generalised aching	Unlikely	Moderate	
**Occurred during Week 2**				
Ivermectin[b]	Nausea, vomiting, diarrhoea, head and body aches. Participant considered sequelae of SARS-CoV-2.	Possibly related	Moderate	
Ivermectin	Tendonitis Likely sports injury.	Not related	Moderate	
Placebo[c]	Pruritis and rash	Related	Moderate	
Placebo[d]	Widespread arthralgia	Possibly related	Mild	
Placebo	Sore legs	Probably related	Mild	
Placebo	Musculoskeletal left chest pain	Unlikely	Moderate	Yes
**Occurred during Week 3**				
Ivermectin	Abdominal pain reminiscent of peptic ulcer pain	Possibly related	Mild	
Ivermectin[b]	Nausea, vomiting, abdominal pain and abnormal liver function tests.	Unlikely	Moderate	Yes
Ivermectin[e]	Dry Mouth	Unlikely	Mild	
Ivermectin	Mouth ulcers	Not related	Mild	
Placebo[d]	Lost/weak voice	Unlikely	Mild	
Placebo	Abdominal pain	Not related	Mild	
Placebo	Cold sores	Unlikely	Mild	
**Occurred during Week 4**				
Ivermectin[e]	Difficulty swallowing.	Unlikely	Mild	
Placebo[a]	Hay fever/allergic rhinitis/asthma	Not related	Mild	

* As assessed by a senior trial doctor.
** Rated as mild if easily tolerated by the participant, causing minimal discomfort and not interfering with everyday activities.
** Rated as moderate if causing sufficient discomfort to interfere with normal everyday activities.
** Rated as severe if it prevents normal everyday activities.
*** SAE (serious adverse event). These have been described in the Results Section of the Main Manuscript.

Table S14B. Adverse events in the trial reported for months 2-6.

Month	Adverse Event	Treatment arm
2	2 cases of SARS-CoV-2 infection	Ivermectin
2	1 hay fever	Ivermectin
2	1 reaction after vaccine; headache, cough, aching	Ivermectin
2	1 fever	Ivermectin
2	1 URTI[a]	Ivermectin
2	1 case of SARS-CoV-2 infection	Placebo
2	1 hay fever	Placebo
2	2 LRTI[b]	Placebo
2	3 URTI	Placebo
2	1 coeliac disease	Placebo
3	1 case of SARS-CoV-2 infection	Ivermectin
3	1 RTI[c]	Ivermectin
3	1 URTI	Ivermectin
3	1 case of SARS-CoV-2 infection	Placebo
3	2 cases of high blood pressure	Placebo
3	1 case of lower abdominal pain	Placebo
3	1 LRTI	Placebo
3	3 URTI	Placebo
4	3 cases of SARS-CoV-2 infection	Ivermectin
4	1 case of chest tightness and cough	Ivermectin
4	1 flare of existing Crohn's disease	Ivermectin
4	1 hair loss	Ivermectin
4	3 URTI	Ivermectin
4	3 URTI	Placebo
4	1 LRTI	Placebo
4	1 Gout	Placebo
4	1 abnormal liver function	Placebo
5	1 case of SARS-CoV-2 infection	Ivermectin
5	1 URTI	Ivermectin
5	I infected tooth root	Ivermectin
5	2 RTI	Placebo
5	1 case of Influenza A	Placebo

6	1 URTI	Ivermectin
6	1 case of vertigo and URTI	Ivermectin
6	1 case of gastroenteritis with hives	Ivermectin
6	1 recurrence of infected tooth root	Ivermectin
6	1 case of SARS-CoV-2 infection	Placebo
6	1 blocked ear	Placebo
6	1 flu-like symptoms after vaccination	Placebo
6	1 LRTI	Placebo
6	4 URTI	Placebo

[a]: URTI, upper respiratory tract infection (not ascribed to SARS-CoV-2)

[b]: LRTI, lower respiratory tract infection (not ascribed to SARS-CoV-2)

[c]: RTI, respiratory tract infection (not ascribed to SARS-CoV-2)

Figure S1. Goodness of fit analysis of the logistic regression model for the primary endpoint.

The goodness of fit of the multivariate logistic regression (with Firth's correction) model for the primary endpoint was assessed graphically by ordering the data observations by risk and then splitting into quintiles, and plotting the observed (i.e. empirical) risk against the model-predicted risk for each quintile. If the goodness of fit is adequate, then the observed and model-predicted risks should be roughly equal and the plot points should thus be close to the diagonal line of equality.

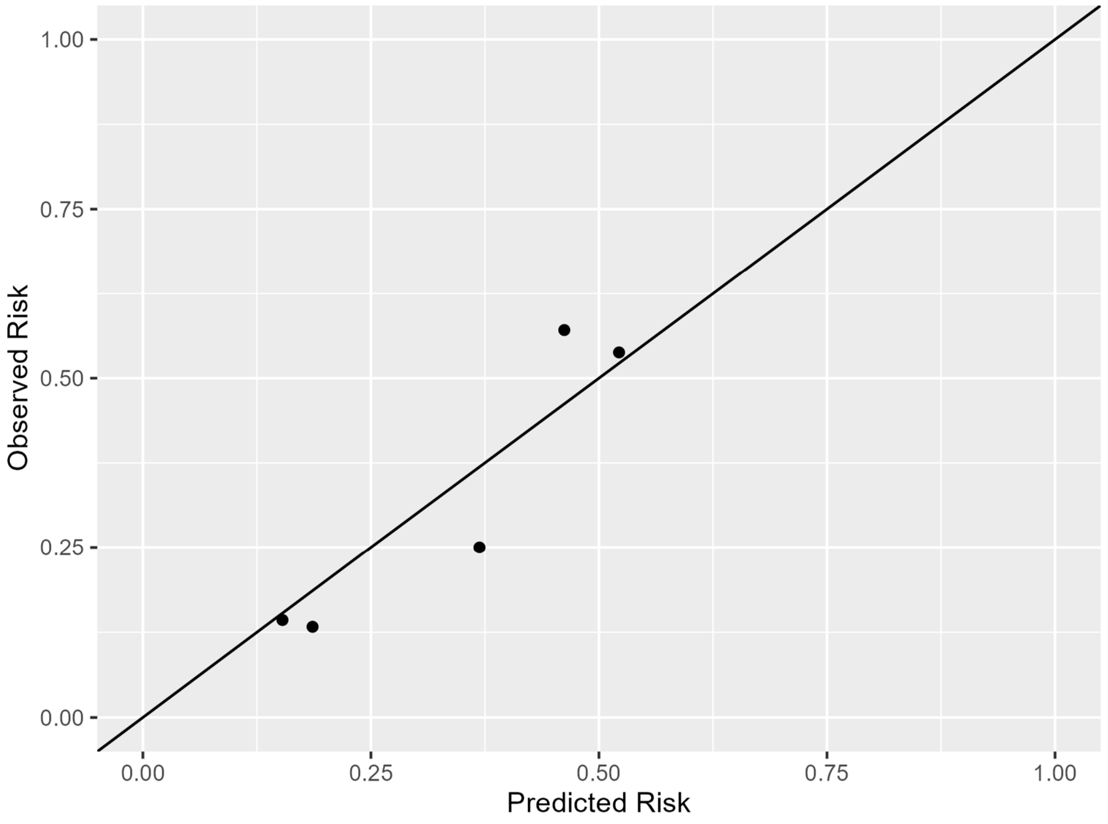

Figure S2. Histogram of Actual Doses Of Ivermectin Received.

Ivermectin was dispensed according to weight aiming to supply 200 ug/kg. As the Ivermectin came in 3 mg tablets, actual Ivermectin dose was not exactly 200 ug/kg for most participants. Actual doses are shown in this Figure. The statistical analysis plan (SAP) calls for the primary endpoint to be re-analysed excluding participants who received an actual Ivermectin dose < 200 μg/kg.

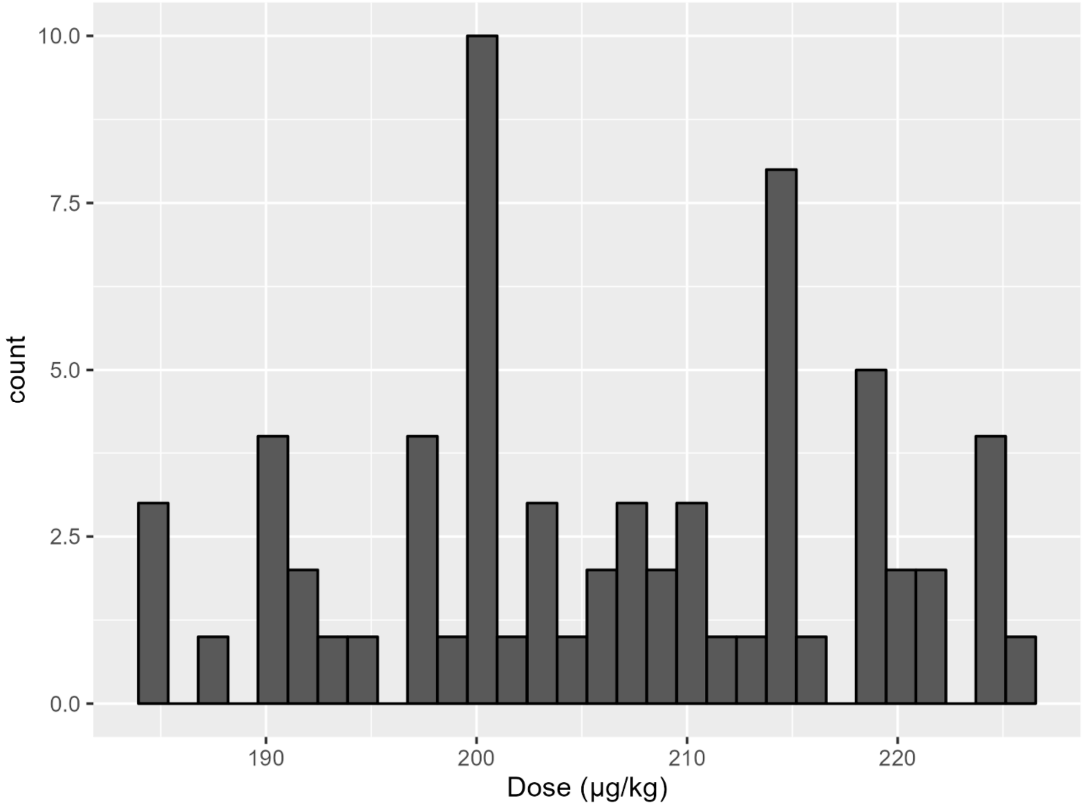

Figure S3. Analysis of Days of Symptoms. Histograms are shown of the number of days reported for each specific symptom (Days 1-28), counting Day of IP as Day 1, plotted according to treatment arm. Note: Y-axis varies between histograms.

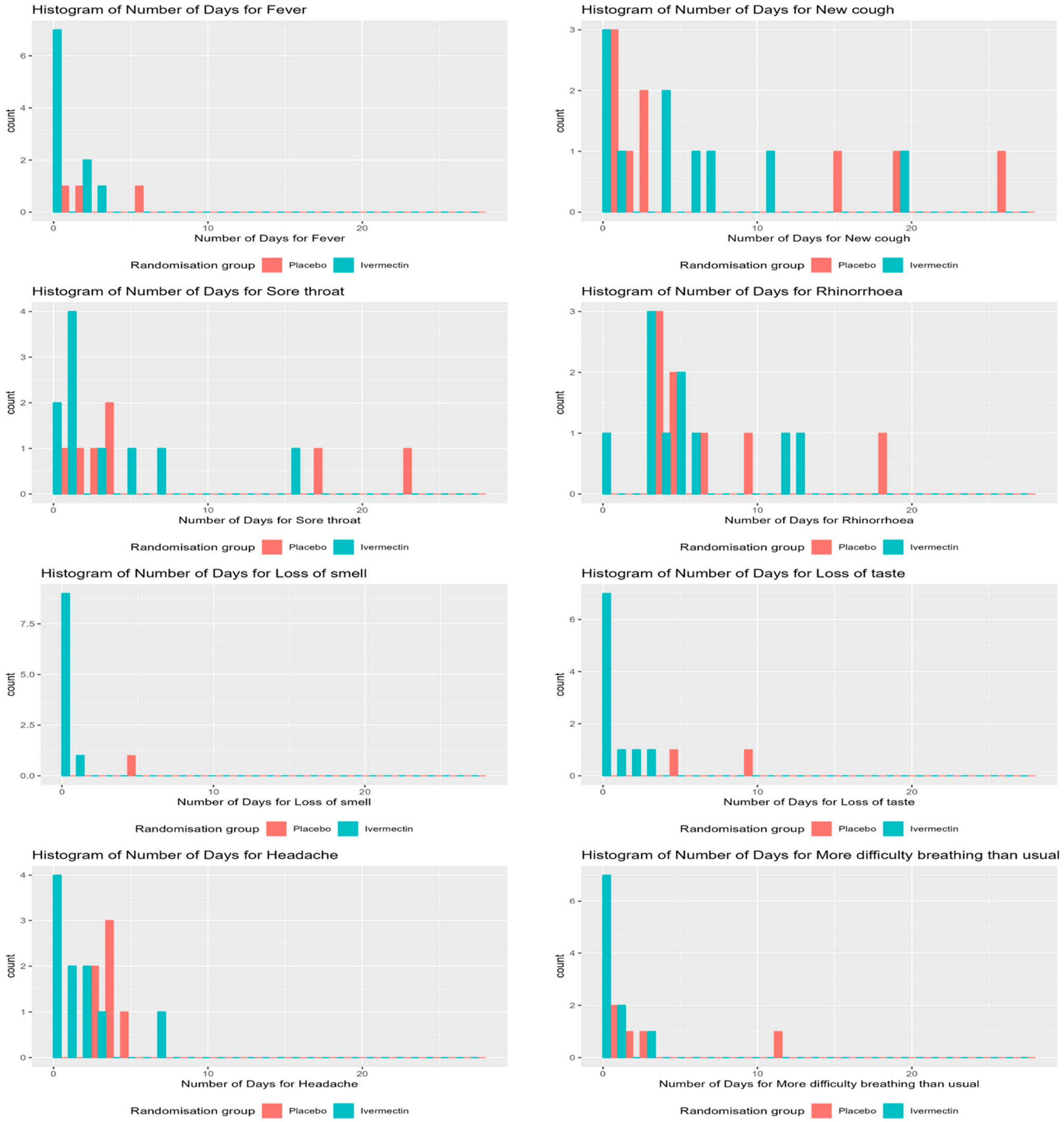

Supplementary Reference

1. Hammond, J.; Fountaine, R.J.; Yunis, C.; Fleishaker, D.; Almas, M.; Bao, W.; Wisemandle, W.; Baniecki, M.L.; Hendrick, V.M.; Kalfov, V.; Simon-Campos, J.A.; Pypstra, R.; Rusnak, J.M. Nirmatrelvir for Vaccinated or Unvaccinated Adult Outpatients with Covid-19. *N. Engl. J. Med.* **2024**, *390*, 1186-1195, doi:10.1056/NEJMoa2309003.
