# Supplementary material for: A Pilot, Randomised, Placebo-Controlled, Double-Blind Trial of a Single Oral Dose of Ivermectin for Post-Exposure Prophylaxis of SARS-CoV-2
Source: Pharmaceutics. 2025 Sep 16;17(9):1205. doi: 10.3390/pharmaceutics17091205 (PMC12473430; doi:10.3390/pharmaceutics17091205)
Supplement: Supplementary file 1 [file pharmaceutics-17-01205-s001.zip › Supplementary Material S1.pdf]

Supplementary Material S1

The following pages contain the Clinical trial protocol approved by Bellberry Human Research Ethics Committee.

Protocol Title: **A pilot randomized placebo-controlled double-blind trial of single dose oral Ivermectin for post-exposure prophylaxis of SARS-CoV-2**

Protocol Number: **EP-Ivermectin-01**

Short Title: **Ivermectin to prevent Coronavirus**

Sponsor Name: **Monash University**

Legal Registered Address: **Monash University**
Wellington Road
Clayton Vic 3800

Version 5.0

Dated: 16 February 2022

CONFIDENTIAL

This document contains information that is privileged or confidential. As such it may not be disclosed unless specific prior permission is granted in writing by the investigators or such disclosure is required by federal laws or regulations.

Principal Investigators:

Dr Mark Stein:	Melbourne, Australia
Kylie Wagstaff, PhD:	Melbourne, Australia
Professor David Jans:	Melbourne, Australia
Dr Jean-Jacques Rajter	Fort Lauderdale, USA
Dr Juliana Cepelowicz-Rajter	Fort Lauderdale, USA

Associate Investigator:

Professor Joseph Torresi	Melbourne, Australia

STUDY ACKNOWLEDGMENT/CONFIDENTIALITY

This document contains information that is privileged or confidential. As such, it may not be disclosed unless specific prior permission is granted in writing by the Sponsor or the investigators or such disclosure is required by federal or other laws or regulations. Persons to whom any of this information is to be disclosed must first be informed that the information is confidential. These restrictions on disclosure will apply equally to all future information supplied, which is indicated as privileged or confidential.

The Sponsor will have access to any source documents from which Case Report Form data may have been generated. The Case Report Forms and other data pertinent to this study are the sole property of the Sponsor and investigators, who may utilise the data in various ways, such as for submission to government regulatory authorities, or in publications of the results of the study. The results of this study may be published. Upon completion of the Study, it is the intention of the parties to prepare a publication regarding or describing the Study and the results therefrom and all parties shall co-operate in this regard.

MEDICAL MONITOR

One or more Medical Monitors will be appointed from each country in which the trial recruits.

This study will be conducted in accordance with the ethical principles that have their origin in the Declaration of Helsinki, and that are consistent with ICH GCP and the applicable regulatory requirement(s).

Protocol Approved By:

Kylie Wagstaff
Principal Investigator,
Monash University

Signature: ....

Date: ....

Monash University (Australian Sponsor): -

Signatory Name:……………………………….

Signature: ....

Date: ....

Table of Contents

1. Protocol Synopsis 7

2. Schedule of Activities 11

3. Introduction 12
3.1 Study Rationale 12
3.2 Background 12
3.3 Benefit/Risk Assessment 15
3.3.1 Risk to the participant 15
3.3.2 Risk to the trial 17

4. Objective and Endpoints 18
4.1 Primary Objective 18
4.2 Primary endpoint 18
4.3 Secondary endpoints 18

5. Study Design 19
5.1 Overall Design 19
5.1.1. Recruitment 20
5.1.2. Method of recruitment 20
5.1.3. Consent 20
5.1.4 Who can take consent? 20
5.1.5 The Consent Process and prescription of Investigational Product 20
5.1.6. Randomization 22
5.1.7. Participant Oversight 23
5.2 Justification for Dose 25
5.3 End of Study Definition 27

6. Study Population 27
6.1 Inclusion Criteria 27
6.2 Exclusion Criteria 28
6.3 Lifestyle Considerations 30
6.3.1 Diet and Activity 30
6.4. Screen Failures 31

7. Study Intervention 31
7.1 Study Intervention(s) Administered 31
7.1.1 Preparation/Handling/Storage/Accountability 32
7.1.2. Mechanism of Randomization and distribution of Treatment/Placebo 32
7.1.3. Pharmacist obtains the randomization outcome 32
7.1.4 Dispensing investigational product and its delivery to participants 32
Recording time of drug administration (time of intervention) 34
7.1.5. Tablets prepared in series not in parallel 34
7.1.6. Pharmacy data and treatment/placebo retention 34
7.1.7. Distribution of trial tablets and recording of drug administration 35

7.1.8. No return of tablets or tablet count 35
7.2. Measures to Minimize Bias: Randomization and Blinding 35
7.2.1. Unblinding Procedures 35
7.3. Study Intervention Compliance 35
7.4 Concomitant Therapy 35
7.5 Dose Modification 36
7.6 Intervention after the End of the Study 36

8. Discontinuation of Study Intervention and Participant Discontinuation/Withdrawal 36
8.1 Discontinuation of Study Intervention 36
8.1.1Temporary Discontinuation 36
8.2. Participant Discontinuation/Withdrawal from the Study 36
8.3. Lost to Follow Up 36

9. Study Assessments and Procedures 37
9.1. Consent 37
9.2. Data collection 37
9.2.1. Screening data 37
9.2.2. Baseline data on Day 1= Day of Treatment 39
9.2.3. Data collection 4 hours after Investigational product delivered 40
9.2.4. Data collection on Days 8, 15, 22, 29 41
9.2.5. Months 2,3,4,5,6 45
9.3. Efficacy Assessments 45
9.4. Safety Assessments 46
9.4.1. Physical Examinations 46
9.4.2. Vital Signs 46
9.4.3. Clinical Safety Laboratory Assessments 46
9.5. Adverse Events and Serious Adverse Events 46
9.5.1. Time Period and Frequency for Collecting AE and SAE Information 47
9.5.2. Method of Detecting AEs and SAEs 47
9.5.3. Follow-up of SAEs 47
9.5.4. Regulatory Reporting Requirements for SAEs 47
9.5.5. Disease-Related Events and/or Disease-Related Outcomes Not Qualifying as SAEs 48
9.6. Treatment of Overdose 48

10. Data management, monitoring and storage 49
10.1. REDCap data entry and backup 49
10.2. Identification or De-identification of data 50
10.2.1 Sponsor access to de-identified participant level data 50

11. Statistical Considerations 51
11.1. Power and sample size 51
11.2. Basic description of participants 51
11.2.1. For the primary endpoints 52
11.2.2. For the secondary endpoints 52
11.2.3. General statistical points 52
11.2.4. Prespecified subgroups 52

11.2.5. Further comment on study design: confounding from secondary exposure to SARS-CoV-2 whilst in isolation 53
11.2.6 Sensitivity analyses 54
11.3. Interim Analyses 54
11.4 Additional endpoints after the 40th participant converts to a positive pharyngeal swab PCR or RAT for SARS-CoV-2. 54

12. Regulatory, Ethical, and Study Oversight Considerations 55
12.1. Human Research Ethics Committee/Institutional Review Board Approval 55
12.2. Regulatory and Ethical Consideration 55
12.3. Financial Disclosure 55
12.4. Informed Consent Process 56
12.5. Data Protection 56
12.6. Committees Structure 56
12.6.1. Data Safety Monitoring Board 56
12.6.2. Trial Steering Committee 58
12.6.3. Medical Monitor 58
12.7. Dissemination of Clinical Study Data 59
12.7.1. Data Quality Assurance 59
12.7.2. Source Documents 60
12.7.3. Pharmaceutical company supplying investigational product access to data for preparation of regulatory applications 60
12.8. Study Closure 60
12.9. Trial Registration 60
12.9. Publication Policy 61

13. List of Abbreviations 62

14. References 63

Appendix 1: Stromectol (Ivermectin) Product Information 67

Appendix 2A: Number of Tablets of Ivermectin (each tablet being 3mg Ivemectin) given according to weight. 81

Appendix 2 B: Tablet Instruction Sheet (Unapproved RAT kits) 82

Appendix 2 C: Tablet Instruction Sheet (Approved RAT kits) 85

Appendix 2D: Trial clinical contact card 88

Appendix 3: Table indicating actual dose (ug/kg) of Ivermectin 90

Appendix 4: Symptom questionnaire 94

Appendix 5: Proforma Letter to General Practitioner/Primary Care Physician 96
Appendix 6: Child-Pugh classification of severity of cirrhosis 99

1. Protocol Synopsis

**Protocol Title:**	**A pilot randomized placebo-controlled double-blind trial of single high dose oral Ivermectin for post-exposure prophylaxis of SARS-CoV-2**	
**Short Title:**	Ivermectin to prevent Coronavirus	
**Rationale**	Ivermectin has in vitro efficacy against SARS-CoV-2 and its oral administration in patients hospitalised with severe pulmonary disease due to SARS-CoV-2 is associated with markedly lower mortality (Caly et al 2020, Cepelowicz-Rajter et al 2020a, Cepelowicz-Rajter et al 2020b).<br>There is an urgent need to establish through a randomized placebo-controlled trial whether this beneficial clinical association is due to a direct effect of Ivermectin.	
**Structure**	The trial will recruit participants who have, in the preceding 72 hours, had close contact with a person infectious with SARS-CoV-2. Participants must have, since that contact, tested negative for SARS-CoV-2 on a polymerase chain reaction (PCR) from a pharyngeal swab or on a rapid antigen test (RAT) and be asymptomatic of: fever, new cough, sore throat, rhinorrhoea, loss of smell, loss of taste, or more difficulty breathing than usual.<br>The trial definition of a positive case of infection with SARS-CoV-2 will be the Australian public health definition of: either a positive result on a pharyngeal PCR for SARS-CoV-2 or a positive result on a TGA-approved RAT for SARS-CoV-2.<br>Participants will be randomized 1:1 to receive either Ivermectin 200ug/kg orally or Placebo on Day 1.<br>The trial will recruit until 40 participants have converted to a positive pharyngeal swab PCR or positive TGA-approved RAT for SARS-CoV-2 within 14 days of their contact with an infectious SARS-CoV-2 index case.	
**Objective and Endpoints**	**Objective**	**Endpoints**
	**Primary**	

	To test whether Ivermectin 200ug/kg orally on Day 1, is superior to placebo as post-exposure prophylaxis for SARS-CoV-2.	• Amongst the 40 participants who convert to a positive PCR or positive TGA-approved RAT for SARS-CoV-2, the proportion who received Ivermectin. Specifically, the trial will test whether that proportion is less than half.
	**Secondary Endpoints**	• Amongst the 40 participants who convert to a positive PCR or positive TGA-approved RAT for SARS-CoV-2, the difference (those who received Ivermectin versus those who received placebo) in days alive free of SARS-CoV-2 symptoms (fever, new cough, sore throat, rhinorrhoea, loss of smell, loss of taste, more difficulty breathing than usual) at Day 14.<br><br>• Amongst the 40 participants who convert to a positive PCR or positive TGA-approved RAT for SARS-CoV-2, the difference (those who received Ivermectin versus those who received placebo) in days alive free of SARS-CoV-2 symptoms (fever, new cough, sore throat, rhinorrhoea, loss of smell, loss of taste, more difficulty breathing than usual) at Day 28.<br><br>• Amongst the 40 participants who convert to a positive PCR or positive TGA-approved RAT for SARS-CoV-2, the difference (those who received Ivermectin versus those who received placebo) in days alive free of presentation to hospital and/or acute hospital care and/or to outpatient care under hospital supervision at Day 28. Acute hospital care will not include days spent in an acute hospital ward solely because a

		rehabilitation or non-acute care facility bed was not available.<br><br>• Amongst the 40 participants who convert to a positive PCR or positive TGA-approved RAT for SARS-CoV-2, the difference (those who received Ivermectin versus those who received placebo) in time from exposure to an index case of SARS-CoV-2 to a positive PCR or positive TGA-approved RAT for SARS-CoV-2.
**Overall Design**	The study is a randomized double-blind placebo-controlled trial of a single oral dose Ivermectin (200 µg/kg) versus placebo on Day 1.<br><br>The study will be conducted as an outpatient study in participants aged 18 to 80 years old.<br><br>Data will be obtained from clinical and laboratory records and phone and electronic inquiry and captured in a REDCap database.<br><br>Potential participants will self-identify or be referred to the Contract Research Organisation (CRO). If a potential participant is interested, a participant information and consent form (PICF) will be provided. Consent will be verbal (as participants are close contacts of a person who has tested positive for SARS-CoV-2) by a doctor or research nurse.<br><br>After consent, information will be forwarded to a clinical trials pharmacy advising that a participant has been recruited to the study.<br><br>The Pharmacist will perform randomization using the REDCap database and then dispense the investigational product (Ivermectin or placebo).<br><br>This will be delivered contactlessly to participants by courier or taxi in Australia and by other means as considered appropriate at overseas trial locations.<br><br>A Trial Steering Committee is responsible for overall management of the study.<br><br>Study management and coordination will be performed by Contract Research Organisations (CROs) in each country.<br><br>One or more Medical Monitors will be appointed for each country in which the trial recruits. Each medical monitor will be a specialist doctor with clinical trials experience. If there is more than one Medical Monitor for a country, those monitors may form and function as a monitoring committee.	

	A Data Safety Monitoring Board (DSMB) is responsible for reviewing the Serious Adverse Events (SAEs) that occur in the study and to address any concerns raised by a Medical Monitor or The Trial Steering Committee.
**Number of Participants**	• The trial aims to recruit until 40 participants convert to a positive pharyngeal PCR or positive TGA-approved RAT for SARS-Cov-2 within 14 days of their exposure to an infectious index case.
**Intervention Groups and Duration**	• Participants are outpatients who are followed weekly for 28 days and subsequently monthly for 6 months. Follow up is by telephone.<br>• Participants will be randomized 1:1 to<br>Ivermectin (200µg/kg) orally on Day 1<br>or<br>Placebo orally on Day 1
**Preparation/ Handling/Storage/**<br><br>**Accountability**	• The study will use clinical trials pharmacies.<br>Ivermectin and placebo tablets will be delivered respectively to each clinical trial pharmacy prior to enrolment of the first participant in that location.<br>Each clinical trial pharmacy must confirm appropriate temperature conditions have been maintained during transit for all study intervention received and any discrepancies are reported and resolved before use of the study intervention.<br><br>• Receipt and dispensing of Ivermectin and placebo will be captured on appropriate pharmacy logs.<br><br>• Clinical trials pharmacies will dispense Ivermectin or placebo for individual participants.<br><br>• Pharmacists will prepare a package for delivery to the individual participant instruction sheet for the investigational product and arrange for the investigational product to be passed to the courier or taxi for delivery to the participants.

2. Schedule of Activities

Procedure	Screening[1]	Consent[1]	Follow Up[3]									
			Treatment (Days)					Longer term AE/SAE follow up (Months±3 days)				
			1[1]	8	15	22	29	2	3	4	5	6
Identification	X											
Inclusion and exclusion criteria	X											
Informed consent		X										
Weight	X[2]											
Height			X[2]									
Randomization			X[1]									
RAT for SARS-CoV-2[4]			X									
Urine pregnancy test[5]			X									
Study treatment			X									
Past and current medical conditions			X									
Concomitant drug and clinical course review			X	X	X	X	X					
AE/SAE review			X[6]	X	X	X	X	X	X	X	X	X

1. Pre-screening, consent, and randomization can occur on the same day. Day 1 is baseline and day of treatment.

2.Self-reported

3.Follow up is by telephone.

4. Participants will have a RAT for SARS-CoV-2 immediately prior to study treatment and will be instructed not to take the study treatment if that RAT is positive or inconclusive (for example if the positive control on that test did not work). Participants who are asymptomatic will have a further RAT for SARS-CoV-2 on Day 2, 3, 4 and 5, and on each of days 6 and 14 following close contact (with the day of close contact counted as day 0). If practical and available, which may depend on public health circumstances, they may be asked to have a PCR for SARS-CoV-2 at days 6 and/or 14 following close contact.

Participants will be instructed that they must present for a pharyngeal swab PCR for SARS-COV-2 whenever symptoms of SARS-CoV-2 present (unless public health instruction is to the contrary at the time of their trial participation) If a positive pharyngeal swab PCR for SARS-CoV-2 result is returned no further pharyngeal swab PCR or RAT for SARS-CoV-2 is performed for that participant.

5. Female participants under age 60 years will perform this immediately prior to study treatment and will be instructed not to take the study treatment if that test is positive.

6.First AE/SAE review will be on Day 1 at 4 hours after IP delivery

3. Introduction

3.1 Study Rationale

Ivermectin has in vitro efficacy against SARS-CoV-2 (Caly et al 2020). Its oral administration to patients hospitalised with severe pulmonary disease due to SARS-CoV-2 is associated with markedly lower mortality (Cepelowicz-Rajter et al 2020a, Cepelowicz-Rajter et al 2020b). Similarly, a retrospective study from Bangladesh reported that a single 12mg dose of Ivermectin (which for a 60kg patient is 200ug/kg) given within 24 hours of admission was associated with lower mortality from SARS-CoV-2 (Khan et al 2020a).

There is thus an urgent need to establish through a randomized placebo-controlled trial whether these beneficial clinical associations are due to a therapeutic effect of Ivermectin.

3.2 Background

In 2012, principal investigators Kylie Wagstaff and David Jans reported that Ivermectin reduced transport of single stranded RNA viral proteins from the cytoplasm to the nucleus by inhibiting a specific nuclear transport pathway, the Importin alpha/beta1 pathway, (Wagstaff et al 2012). When the current pandemic arose, they hypothesized that this pathway would be amenable to intervention in SARS-CoV-2 because that virus was also a single stranded RNA virus. The resulting experiments demonstrating in vitro inhibition of SARS-CoV-2 replication by Ivermectin published in April 2020 (Caly et al 2020) sparked global interest in the use of Ivermectin in this pandemic.

Within Australian infectious diseases circles, concern was expressed that the in vitro concentration used in that paper (Caly et al 2020) was not clinically attainable in plasma.

There are three key scientific responses to that concern. First, the initial in vitro experiment (Caly et al 2020) examined SARS-CoV-2 in Vero-hSLAM cell cultures. Vero cells are African Green Monkey renal fibroblasts. More recently, in a more appropriate cell line, Kylie Wagstaff and David Jans have demonstrated an IC50 which was lower. In this respect, the in vitro data from Vero cells could instead be considered a proof of concept of a molecular pathway, rather than a definitive indicator of the IC50.

Second, The Antiviral Res paper (Caly et al 2020) is an in vitro study; other peer-reviewed studies address the question of dosing, and in particular the question of concentrations of Ivermectin in relevant tissues, as opposed to serum. Arshad et al (2020), for example in their Figure 5, report modelled human tissue Cmax/EC 50 ratios for Ivermectin. They developed their data from the data in the paper of Caly et al (2020)) and they modelled following a single oral dose of 200ug/kg Ivermectin (see also the Supplementary Table 2 of Arshad et al 2020). Their modelling found human tissue Cmax/EC50 ratios of: lungs 21.02, kidney 16.10, gut 34.39, liver 17.22, brain 32.99, heart 13.92, bone 14.92, muscle 10.14, pancreas 35.87, skin 51.40, spleen 8.6 and thymus 16.32 Furthermore, Schmith et al (2020) cite their reference 4 to state that “ivermectin concentrations remained steady in cattle lungs for 8 days after a single subcutaneous dose”.

Third, there are clinical data from two other principal investigators (Jean-Jacques Rajter and Juliana Cepelowicz-Rajter), who, aware of the in vitro data above, administered Ivermectin to patients hospitalised with SARS-CoV-2 and published that Ivermectin administered as 200 µg/kilogram orally, with a potential second such oral dose after one week, is associated with reduced mortality amongst inpatients hospitalised with SARS-CoV-2 infection (ICON study, Ceplowicz-Rajter et al 2020a and 2020b).

In particular, they reported a retrospective analysis of 280 non pregnant adult patients consecutively admitted mid-March to mid-May 2020 with SARS-CoV-2 to Broward County Hospitals (Florida, United States of America). They call this the ICON study. 173 patients received 200ug/kg Ivermectin as a single dose orally during their admission "in addition to usual clinical care" (13 of these received a second such dose on day 7 "since they were still hospitalized"). 107 patients received "usual care". Participants were of mean (+-standard deviations) age 59.6 (17.9) years.

Ivermectin use was associated with lower mortality. In univariate analysis the OR for Ivermectin versus usual care was 0.52 (0.29 to 0.96), P = 0.03. Severe pulmonary involvement at study entry was characterized as "an FiO2 of 50% or greater, high-flow nasal oxygen, noninvasive ventilation, or intubation and mechanical ventilation". In the subgroup of 75 patients who had severe pulmonary involvement, the OR for Ivermectin versus usual care was 0.15 (0.05 to 0.47), P = 0.001.

More of the "usual care" patients received hydroxychloroquine or the combination hydroxychloroquine plus azithromycin (97.2 vs 90.2%, P = 0.03 and 91.6 vs 81.7 %, P =0.04). However, Ivermectin use was independently associated with mortality. "In the multivariate analysis, adjusting for demographic factors, between-group differences in mortality risks, and concomitant use of hydroxychloroquine (with or without azithromycin), independent predictors of in-hospital mortality included treatment group, age, severe pulmonary disease category, and reduced lymphocyte count." In a "multivariate analysis of factors associated with mortality" the OR (CI) for Ivermectin was 0.27 (0.09, 0.85) (Table 4 of Cepelowicz-Rajter et a 2020a). "Similarly, the Cox regression showed ivermectin was associated with a significantly lower hazard ratio for mortality of 0.37 (CI 0.19 - 0.70, p=.003)" (Cepelowicz-Rajter et al 2020a).

The ICON investigators have since reported a propensity analysis (Cepelowicz-Rajter et al 2020b). Again, the point estimate of the OR for mortality overall and the OR for mortality in the subgroup who presented with severe pulmonary involvement, both reduced in the propensity analysis compared with the unmatched analysis, counting against confounding variables leading to the beneficial association.

Importantly, Jean-Jacques Rajter and Juliana Cepelowicz-Rajter and their colleagues noted in the ICON peer-reviewed report "Because of the retrospective observational nature of the study, despite adjustment for known confounders and propensity score matching, we cannot exclude the possibility of unmeasured confounding factors" and drew attention to the need for randomized controlled trials (Cepelowicz-Rajter et al 2020b).

Similarly, an editorial by authors with research interest in Ivermectin in the setting of tropical diseases (Chaccour et al 2020a) stated: "…We believe the recent findings regarding ivermectin warrant rapidly implemented controlled clinical trials to assess its efficacy against SARS-CoV-2." They do, however, highlight potential areas of caution with such trials.

The current trial's rationale to test the efficacy of Ivermectin is independent of any potential mechanisms of action of Ivermectin. There could be many mechanisms of action and the current trial does not rely on a single postulated mechanism of action as the critical rationale for this trial.

Other mechanisms which may be operative include: that Ivermectin appears to have direct immune effects in various animal models (which have been reported as stimulatory or repressive depending on the system) including a capacity to affect T-cells (Corbo-Rogers et al 2012) and to affect response to antigen (Blakley, Rousseaux 1991, Sajid et al 2007, Stankiewicz et al 1995, Ventre et al 2017) and see also the Discussion of Chaccour et al (2021) for other potential mechanisms of action. Ivermectin has a long half-life and is metabolised in vivo. The effect of these metabolites and/or the long persistence of the drug may not have been apparent in the in vitro model of Caly et al (2020).

Regardless of potential mechanism(s) of action, the clinically important beneficial association of Ivermectin 200ug/kg with lower mortality from SARS-CoV-2, an association which improved after adjustment for known confounders, is a sufficient rationale for a randomized placebo-controlled trial to test whether Ivermectin is therapeutic in SARS-CoV-2.

Two trials in particular have tested Ivermectin administered early after SARS-CoV-2 infection. Chaccour et al (2020a, 2021) randomized participants already infected by SARS-CoV-2 within 72 hours of symptoms to receive Ivermectin 400ug/kg or placebo. They sought a therapy which would "reduce the transmission of SARS-CoV-2 when administered early after disease onset". The primary outcome of PCR positivity at Day 7 was not different between the Ivermectin and placebo cohorts (each n = 12). Secondary and post-hoc analyses were, however, not inconsistent with a benefit from Ivermectin.

Biber et al (2021) treated participants a median 4 days after symptom onset with SARS-CoV-2 with Ivermectin 200ug/kg versus placebo daily for 3 days. In their non-peer reviewed report, Ivermectin treatment was a predictor in a logistic regression of a Day 6 high PCR cycle threshold (Ct) (>30), which they considered a non-infective level.

Meta-analyses have considered Ivermectin prophylaxis data for SARS-CoV-2. Popp et al (2021) reported a risk ratio (95% confidence interval) of 0.13 (0.08 to 0.21) for prevention of SARS-CoV-2 infection by Ivermectin (see their Analysis 3.1) This favours Ivermectin but was only based on one study and Popp et al (2021) noted significant limitations with the methodology of that study. Bryant et al (2021) analysed three Ivermectin SARS-CoV-2 prevention trials and wrote: "Ivermectin prophylaxis versus no ivermectin prophylaxis. Three studies involving 738 participants evaluated ivermectin for COVID-19 prophylaxis among health care workers and COVID-19 contacts. Meta-analysis of these 3 trials, assessing 738 participants, found that ivermectin prophylaxis among health care workers and COVID-19 contacts probably reduces the risk of COVID-19 infection by an average of 86% (79%–91%) (3 trials, 738 participants; a RR 0.14, 95% CI 0.09–0.21; 5.0% vs. 29.6% contracted COVID-19, respectively; low-certainty evidence; downgraded due to study design limitations and few included trials) (Figure 15). In 2 trials involving 538 participants, no severe adverse events were recorded (SoF Table 4)."

At the height of the pandemic in Victoria, Australia (winter 2020), public health SARS-CoV-2 PCR testing was largely limited to people with symptoms and test results could take 5 days to

return. Now in Australia, PCR testing of asymptomatic close contacts of infectious index cases (for example Victorian Department of Health and Human Services Tier 1 exposure) commonly provides results within 12-24 hours.

This change in the availability and speed of public health testing, together with the rapid communication to the public of SARS-CoV-2 exposure times and sites, provides an opportunity, which was not present earlier in the pandemic, to test Ivermectin's efficacy for post exposure prophylaxis to prevent SARS-CoV-2 disease.

Another important variable not always present in Australia earlier in the pandemic, is the very low community incidence and prevalence of SARS-CoV-2. This is important for a post-exposure prophylaxis community based RCT because it reduces confounding that would occur if such an RCT was conducted in a setting of high community incidence and prevalence. In the latter setting, the possibility of unknown/unwitting exposure of participants to multiple other index cases at variable times prior to RCT enrolment could confound the interpretation of the RCT.

There is thus a unique opportunity in Western countries with low SARS-CoV-2 prevalence and rapid contact identification and population testing to now conduct a community based RCT of Ivermectin for post-exposure prophylaxis against SARS-CoV-2. If such an RCT was positive, it would provide an important therapeutic tool to limit further outbreaks in such societies and buttress the case for testing Ivermectin in established SARS-CoV-2.

3.3 Benefit/Risk Assessment

"Ivermectin, a highly active broad-spectrum antiparasitic agent, is approved in Australia and is indicated for the treatment of:

a) onchocerciasis and intestinal strongyloidiasis (anguillulosis).

b) crusted scabies in conjunction with topical therapy.

c) human sarcoptic scabies when prior topical treatment has failed or is contraindicated."

(Stromectol Product Information _ Appendix 1)

Ivermectin has been used in large numbers of people worldwide to treat parasitic diseases for approximately 40 years with a favourable side effect profile in that setting (Crump and Omura 2011 and see also Chaccour et al 2017 who consider Ivermectin pharmacokinetics and pharmacodynamics with respect to reducing malaria transmission).

3.3.1 Risk to the participant

The risk of side effects in the context of the very wide beneficial international experience with this drug in other settings has been noted above (including as discussed by Crump and Omura 2011).

Most of that use has been doses of 150-200ug/kg. This RCT will administer 200ug/kg orally. The reason for this dose is discussed in Section 5.2 "Justification of dose" below. However with reference to risk, the work of Guzzo et al (2002) is also relevant.

Guzzo et al (2002) administered Ivermectin to healthy 18-45 year olds, (50-90kg), non- smoking

men and women. They explained “Panel 1 subjects received treatment A: ivermectin 30 mg (347-594 µg/kg) or placebo, three times a week (days 1, 4, 7), in the fasted state during study week 1. After a washout period of at least 1 week, they then received treatment E: ivermectin 30 mg (347-541 µg/kg) or placebo, single dose, in the fed state during study week 3. Panel 2 received treatment B: ivermectin 60 mg (713-1091 µg/kg) or placebo, three times a week (days 1, 4, 7), in the fasted state during study week 5. Panel 3 received treatment C: ivermectin 90 mg (1031-1466 µg/kg) or placebo, single dose, in the fasted state during study week 9. Panel 4 received treatment D: ivermectin 120 mg (1404-2000 µg/kg) or placebo, single dose, in the fasted state during study week 11.” Safety monitoring included: neurological examination, pupillometry, vital signs, ECG, CBC, blood chemistry and urinalysis. They reported “No indication of CNS toxicity associated with oral ivermectin was observed for any of the doses administered in this study. Two subjects developed increases in LFTs during the study, 1 on ivermectin 30 mg and 1 on placebo. The subject on active drug was subsequently identified as having cholelithiasis, which was unlikely to be related to acute treatment with ivermectin. No other clinically significant LFT abnormalities occurred despite dose escalation to 120 mg. These data, together with extensive animal and human exposure, suggest that routine monitoring of laboratory values is unnecessary at the dose levels anticipated for headlice treatment.”

The ICON study (Cepelowicz-Rajter et al 2020a) retrospectively studied standard dose Ivermectin (200ug/kg) in 173 patients hospitalised with SARS-CoV-2 (13 of whom received an identical repeat dose a week later) and reported “We also did not observe any significant side-effect from Ivermectin use.”

In a non-SARS-CoV-2 setting, in the IVERMAL study (which investigated Ivermectin’s efficacy against mosquitoes which could spread malaria), 47 participants received received 600ug/kg Ivermectin daily for 3 days without serious adverse effect (Smit et al 2018) although one participant had anaphylaxis which may have related to Ivermectin. (In this study participants also received another drug, dihydroartemisinin-piperaquine). Smit et al (2018) wrote: “The highest dose (600 µg/kg per day) ivermectin recipients had more adverse events than did those who received placebo (table 2). Treatment-related adverse events showed evidence of a dose-response, predominantly reflecting transient minor visual disturbances (tables 2, 3; appendix (of Smit et al 2018)). All adverse events were non-severe (grade ≤2), except for three patients with pre-existing liver enzyme elevations (two at grade 1 and one at grade 3) who showed grade 3 elevations after treatment (appendix (of Smit et al 2018)).”

The risks may be higher in patients who are older and/or have dementia or structural or functional lesions of the blood brain barrier and/or who take other medication (see: Barkwell, Shields 1997a, Barkwell Shields 1997b, Baudou et al 2020, Bredal 1997, Coyne, Addiss 1997, Currie and McCarthy 2010, Edwards 2003, Reintjies, Hock 1997). In particular, Barkwell and Shields (1997a) reported an observation of excess deaths in a residential care facility in the 6 months after treatment with Ivermectin. In addition, The Product Information of Stromectol states: “Clinical studies of STROMECTOL did not include sufficient numbers of elderly subjects aged 65 years and over to determine whether they respond differently from younger subjects. In general, treatment of elderly patients should be cautious, reflecting the greater frequency of decreased hepatic, renal or cardiac function, and of concomitant disease or other drug therapy.”

Baudou et al (2020) explain: “This drug (Ivermectin) is reputed to be remarkably safe thanks to its ability to be effluxed by the ATP-binding cassette subfamily B member 1 (ABCB1) transporter (also known as MDR1 and P-glycoprotein) in the blood–brain barrier.” They explain that in the Collie dog which has an abnormal P-glycoprotein and in a mouse model of absent P-glycoprotein, “ivermectin induces neurologic disorders that can be fatal.” They continue: “Few cases of neurologic disorders after ivermectin treatment have been reported in humans, and data are lacking on such a deleterious mutation in the human gene ABCB1.” They then “report the case of a 13-year-old boy admitted to the pediatric intensive care unit for impaired consciousness. He had received a single oral dose of ivermectin (0.23 mg per kilogram of body weight) to prevent scabies infection 2 hours 30 minutes before the onset of impaired consciousness. His condition worsened 6 hours after he received ivermectin, with persistent neurologic signs, including coma, ataxia, pyramidal signs, and binocular diplopia, as well as abdominal pain and vomiting. He was monitored for 48 hours; during this period, he had a fluctuating Glasgow score and normal results on paraclinical tests. He fully recovered after 48 hours.” Baudou et al (2020) later demonstrated that the 13 year old had inherited an abnormal gene from each parent for the P-glycoprotein which transports Ivermectin out of the brain.

Chandler wrote about “Serious neurological adverse events after Ivermectin” (Chandler 2018). She reviewed cases of neurotoxicity amongst whom she identified 2 potential fatalities. One was an 81 year old who received 12mg Ivermectin on Day 0 and Day 7. A second was a 64 year old who received 3 oral and 2 subcutaneous doses of Ivermectin 12mg every 48 hours in the setting of multiorgan failure and S.Stercoralis hyperinfection after sepsis after aortic valve replacement. In her conclusion about serious neurological adverse events after Ivermectin, she wrote: “Potential explanations include concomitantly administered drugs which inhibit CYP3A4 and polymorphisms in the mdr-1 gene. By comparison with the extensive post marketing experience with ivermectin in the successful treatment of parasitic infections, the total number of reported cases suggests that such events are likely rare. However, elucidation of individual-level risk factors could contribute to therapeutic decisions that can minimize harms. Further investigation into the potential for drug interactions and explorations of polymorphisms in the mdr-1 gene are recommended.”

In view of the risks factors for neurological toxicity noted in the three paragraphs immediately above, the trial attempts to reduce the risk to each participant by excluding: those taking other medication which potentially strongly inhibits both P-Glycoprotein activity and CYP3A4/5 activity (CYP3A4/5 metabolises Ivermectin) (see Supplementary Appendix of Baudou et al 2020), those aged over 80 and those with clinical history suggesting a risk of dementia and/or impaired blood brain barrier (See Section 6.2, Exclusion Criteria). The trial also excludes those living alone to reduce the risk that a participant could experience a serious event without someone else knowing about it and summoning an ambulance if the participant was unable to do so.

3.3.2 Risk to the trial

With respect to recruitment, if the incidence of SARS-CoV-2 falls, recruitment will fall.

The National Statement on Ethical Conduct on Human Research (2018) notes “Research is ethically acceptable only when its potential benefits justify any risks involved in the research”. In

this trial a widely used drug with good safety profile is being tested during a viral pandemic, which causes significant morbidity and mortality. If Ivermectin is efficacious as post-exposure prophylaxis for SARS-CoV-2 in preventing disease it could also serve, in the words of Chaccour et al (2021), to "reduce the transmission of SARS-CoV-2 when administered early." A therapy which prevented SARS-CoV-2 disease and thus stopped transmission would be an important contribution to pandemic public health measures. The potential benefit of the trial both to participants and the wider community appears to justify the risks.

4. Objective and Endpoints

4.1 Primary Objective

- To test whether Ivermectin 200ug/kg orally on Day 1, is superior to placebo as post-exposure prophylaxis for SARS-CoV-2.

4.2 Primary endpoint

Amongst the 40 participants who convert to a positive PCR or positive TGA-approved RAT for SARS-CoV-2, the proportion who received Ivermectin. Specifically, the trial will test whether that proportion is less than half.

This is a post-exposure prophylaxis trial. The endpoint of positive pharyngeal swab PCR or positive TGA-approved RAT for SARS-CoV-2 was selected, rather than symptomatic SARS-CoV-2 infection, because it is less subjective. It is also in keeping with the past Australian public health clinical endpoints that those people who were isolating after exposure to an infectious case of SARS-CoV-2 required a negative pharyngeal swab PCR or a negative rapid antigen test result for SARS-CoV-2 before ending the isolation.

4.3 Secondary endpoints

- Amongst the 40 participants who convert to a positive PCR or positive TGA-approved RAT for SARS-CoV-2, the difference (those who received Ivermectin versus those who received placebo) in days alive free of SARS-CoV-2 symptoms (fever, new cough, sore throat, rhinorrhoea, loss of smell, loss of taste, more difficulty breathing than usual) at Day 14.

- Amongst the 40 participants who convert to a positive PCR or positive TGA-approved RAT for SARS-CoV-2, the difference (those who received Ivermectin versus those who received placebo) in days alive free of SARS-CoV-2 symptoms (fever, new cough, sore throat, rhinorrhoea, loss of smell, loss of taste, more difficulty breathing than usual) at Day 28.

- Amongst the 40 participants who convert to a positive PCR or positive TGA-approved RAT for SARS-CoV-2, the difference (those who received Ivermectin versus those who received placebo) in days alive free of presentation to hospital and/or acute hospital care and/or to outpatient care under hospital supervision at Day 28. Acute hospital care will not include days spent in an acute hospital ward solely because a rehabilitation or non-acute care facility bed was not available.

- Amongst the 40 participants who convert to a positive PCR or positive TGA-approved RAT for SARS-CoV-2, the difference (those who received Ivermectin versus those who received placebo) in time from exposure to an index case of SARS-CoV-2 to a positive PCR or positive TGA-approved RAT for SARS-CoV-2.

5. Study Design

5.1 Overall Design

The study is a randomized double-blind placebo-controlled trial of a single oral dose 200 µg/kg Ivermectin versus placebo on Day 1.

The study will be conducted as an outpatient study in participants aged 18 to 80 years old.

Data will be obtained from clinical and laboratory records and phone and electronic inquiry and captured in a REDCap database.

Potential participants will self-identify or be referred to the Contract Research Organisation (CRO). If a potential participant is interested, a participant information and consent form (PICF) will be provided. Consent will be verbal (as participants are close contacts of a person who has tested positive for SARS-CoV-2) by a doctor or research nurse.

After consent, information will be forwarded to a clinical trials pharmacy advising that a participant has been recruited to the study.

The Pharmacist will randomize using the REDCap database and then dispense the investigational product (Ivermectin or placebo)

This will be delivered contactlessly to participants by courier or taxi in Australia and by other means as considered appropriate at overseas trial locations.

A Trial Steering Committee is responsible for overall management of the study.

Study management and coordination will be performed by Contract Research Organisations (CROs) in each country.

One or more Medical Monitors will be appointed for each country in which the trial recruits. Each medical monitor will be a specialist doctor with clinical trials experience. If there is more

than one Medical Monitor for a country, those monitors may form and function as a monitoring committee.

A Data Safety Monitoring Board (DSMB) is responsible for reviewing the Serious Adverse Events (SAEs) that occur in the study and to address any concerns raised by a medical monitor or The Trial Steering Committee.

5.1.1. Recruitment

The trial aims to recruit until 40 participants have converted to a positive pharyngeal swab PCR or RAT for SARS-CoV-2 within 14 days of their exposure to an infectious index case.

5.1.2. Method of recruitment

Potential participants will self-identify or be referred to the CRO. Self-identification may be by telephoning the CRO or by indicating a wish to hear more about the trial by entering contact details on a trial website or sending them to a trial email address. These participants may have heard about the trial from a variety of possible sources including electronic/print and/or social media publicity and/or publicity from laboratories/organisations performing PCR or RAT testing for SARS-CoV-2 and/or public health authorities. If a potential participant is interested, a participant information and consent form (PICF) will be provided electronically, as these participants will have had recent contact with an infectious index case of SARS-CoV-2. Participants may also be referred directly to the CRO by public health officials/organisations and/or the doctors who are monitoring them and/or who are their usual treating doctors. Such referral would be done after such organisations/public health officials and/or doctors asked for and received permission to refer from a potential participant, in keeping with each organisation’s policy relevant to such referral for clinical trials.

5.1.3. Consent

5.1.4 Who can take consent?

A trial research nurse, trial doctor and/or investigator who may or may not also be a member of the usual treating medical team of the potential participant. To ensure potential participants are able to be consented 7 days a week, the task of consenting will be delegated by the Principal Investigators to doctors hired for this purpose. These doctors will be fully trained in the study protocol and procedures and will also need to demonstrate their competency via GCP certification and adequate completion of a training quiz provided for this study.

5.1.5 The Consent Process and prescription of Investigational Product

If a potential participant is identified, he/she will be contacted and screened by telephone by an employee of a CRO, a trial research nurse or a trial doctor according to the inclusion and exclusion

criteria, including self-reported weight. If the potential participant then appears suitable for the trial, he/she will be invited to participate. A PICF will be provided electronically to potential participants, or the person responsible, for review. If a potential participant indicates a wish to enroll in the study, a trial research nurse, doctor or investigator will ask for consent.

As the potential participant is a close contact of a case of SARS-CoV-2 and is potentially infectious, written consent and acceptance and retention of a written consent form is not appropriate because of cross-infection risk. A trial research nurse, doctor or investigator will obtain verbal consent. In Australia this will be performed by a doctor working for the trial. This study's approach of verbal consent is in keeping with other approved SARS-CoV-2 community studies (including for example the Australian study TPEP-Corona-1, (see Australia New Zealand Clinical Trials Registry ACTRN12620000982910)). The dialogue between the person taking consent and the participant will not be guided by a prepared script.

Immediately following Consent, a doctor will prescribe the Investigational Product. In Australia the process will be as follows. The doctor who obtained verbal consent will prescribe the Investigational Product using medical prescribing software which will generate a verification barcode on the printed prescription. The doctor will transmit electronically an image of that prescription to the CRO who will forward it to a trial pharmacy for immediate dispensing. The doctor will post the hard copy of that prescription to the CRO who will forward the hard copy to the trial pharmacy. This is in keeping with current clinical practice in the pandemic in which prescriptions with electronic verification barcodes are dispensed based on images of the prescriptions transmitted electronically with the hard copy arriving after the event at the pharmacy for storage in the pharmacy

The consenting doctor will instruct the participant of the need to be at home for the investigational product delivery and if required by the participant, print and sign a medical certificate stating the participant is participating in a clinical trial and for that trial is required to be at home for the investigational product delivery. The consenting doctor will transmit electronically an image of that signed certificate to the CRO who will forward it electronically to the participant.

The consenting doctor will instruct the participant to perform a rapid antigen test for SARS-CoV-2 upon receipt of the investigational product and not to take the tablets if that test is positive or inconclusive (for example if the positive control on that test did not work). Participants will be instructed that those who are asymptomatic are to have a further RAT for SARS-CoV-2 on Day 2, 3, 4 and 5, and on each of days 6 and 14 following close contact (with the day of close contact counted as day 0).

Participants will be instructed that they must present for a pharyngeal swab PCR for SARS-COV-2 whenever symptoms of SARS-CoV-2 present (unless public health instruction is to the contrary at the time of their trial participation). If a positive pharyngeal swab PCR for SARS-CoV-2 result is returned no further pharyngeal swab PCR or RAT for SARS-CoV-2 is performed for that participant.

The PICF will explain that participants may be required to have a pharyngeal swab PCR for SARS-CoV-2 at day 6 and/or 14 following close contact (with day of close contact counted as day 0) if they have remained asymptomatic to that point of time. Although at the time of writing this is not available under the recent government guidelines, these public health rules may change. Participants will be advised in the PICF that this PCR may or may not be available, possible or practical, including as public health guidelines change and that they will accordingly be advised shortly before their day 6

and/or 14 by the CRO whether such a test should be done. They will be provided with a request form written by a consenting doctor should such a test be possible.

The consenting doctor will advise each participant that if at any stage during the trial the participant receives a notification of a positive RAT or PCR result for SARS-CoV-2, that participant should immediately telephone (in this order) 1) the public health authority to confirm they are aware of that result and to ask what management is required 2) his/her GP to confirm the GP is aware of that result and to discuss management.

5.1.6. Randomization

Participants will be randomized 1:1 to

Ivermectin 200μg/kg orally on Day 1

OR

Placebo orally on Day 1

Randomization will be by a clinical trials pharmacist using the REDCap database using concealed permuted block randomization.

Randomization will be stratified:

First by Coronavirus vaccination status, as: unvaccinated, received first vaccination, received second vaccination in the last 10 calendar days, received the second vaccination more than 10 calendar days but less than 6 months prior to Consent, received the second vaccination 6 or more months prior to Consent, received a third vaccination. (If a participant fulfils both the last category and any other category, that participant is allotted to the last category).

And

Second by Exposure site: i) a home or ii) an indoor work environment or iii) a family gathering or a social or a religious function or a ceremony each being of less than 30 people.

5.1.6.1. Process of Randomization

A clinical trials pharmacist will randomize using the REDCap database.

The number of placebo tablets for a participant randomized to placebo tablets will be numerically equal to the number of tablets that participant would have received had he/she been randomized to Ivermectin.

5.1.7. Participant Oversight

- Participants have been exposed to an infectious index case of SARS-CoV-2. As such, they cannot be called in to Australian hospital clinics for routine review nor are there resources for community visits with infection control procedures.
- Participants' care during the trial will be by their usual treating medical team and/or by public health authority staff and not by trial staff. This is in keeping with an HREC approved approach of an Australian community- based treatment trial for SARS-CoV-2 (see Australia New Zealand Clinical Trials Registry (ACTRN) trial number 12620000982910).

In keeping with this, the following apply.

- Prior to recruitment,

  The relevant public health authority for that geographic area will be informed of the trial and that it is about to recruit.

- Immediately following a participant consenting:

  The consenting doctor will telephone the practice of that participant's general practitioner/primary care physician.

  The consenting doctor will seek to speak with that practitioner (or if he/she is unavailable a practitioner colleague or if there is no doctor present, will endeavour to leave a message for a doctor at that practice with a call back contact phone number).

- The consenting doctor will advise:

  That the participant has self-identified as a close contact of an index case of SARS-CoV-2,

  That the participant has identified that practitioner as his/her general practitioner/primary care physician,

  That the participant has consented to participate in this trial and has been prescribed a single oral dose of 200ug/kg Ivermectin or placebo,

  That a letter will follow electronically with references to more information about the trial,

  That the trial is NOT offering clinical care or monitoring which is left to that practitioner,

  And the consenting doctor will give a contact phone number should the practitioner wish to contact a trial doctor.

- Immediately following a participant consenting, the CRO will forward

  A letter by fax or email addressed to the attention of that participant's general practitioner/primary care physician (Appendix 5).

A copy of that same letter by fax or email addressed to the attention of the practice principal of that participant's general practitioner's practice/primary care physician's practice

- It will explain that:

  The participant has advised the trial team that the participant is a close contact of an index case of SARS-CoV-2,

  The participant has consented to join this trial and has been prescribed a single oral dose of Ivermectin 200ug/kg or placebo.

  That the participant has identified that practitioner as his/her general practitioner/primary care physician.

  That whilst the study will be gathering follow up data by telephone, the trial is not clinically examining or monitoring the participant and leaves this to the general practitioner's rooms.

  Provides a reference to the Australian New Zealand Clinical Trials Registry website summarizing the trial,

  Directs the practitioner to immediately accessible copies of the Product Information for Ivermectin,

  Provides a contact phone number should that practitioner wish more information about the trial or wish to speak with a doctor associated with the trial.

- Participants are given the following written instruction both in the PICF and in the Instructions to their symptom diary

  "NB: This trial is gathering information but is not providing medical care.

  If you develop any symptom, if you feel it is an emergency call an ambulance immediately on (insert country specific ambulance phone number).

  If you don't feel it is an emergency, you must telephone your usual general practitioner and remind the doctor you are a close contact of a person with Coronavirus.

  You must also telephone your local public health agency on this 24 hour telephone number (insert country specific number)."

- Note that participants are excluded from enrolment if they cannot provide the name, address and phone number of a general practitioner/primary care physician AND confirm attendance at the rooms of such a practitioner within the last 12 months (such attendance may have been by telehealth given the pandemic) (see Exclusion Criteria, Section 6.2).
- However, in the subsequent event that a nominated general practitioner/primary care

physician denies that the participant is a patient of his/her practice (including of his/her colleagues), then:

- The CRO will attempt to contact the participant to see if there is a different general practitioner/primary care physician attended within the last 12 months.
- If there is,

  A doctor associated with the trial will telephone that general practitioner/primary care physician as above and the letters above will be transmitted electronically to that general practitioner/primary care physician and to the practice principal of that practice.
- If there is not,

  A doctor associated with the trial will telephone the relevant public health authority to advise that:

  There is a close contact of an index case of SARS-CoV-2 who has enrolled in the trial but whose nominated general practitioner/primary care physician denies being their general practitioner/primary care physician,

  That this person has consented to participate in this trial which is already known to that public health authority (as above),

  That the participant has been prescribed a single oral dose of 200ug/kg Ivermectin or placebo,

  That whilst the study will be gathering follow up data by telephone, the trial is not clinically examining or monitoring the participant,

  That the trial will leave to that public health authority to arrange ongoing clinical care and monitoring for that member of the public who does not have a general practitioner/primary care physician and is a close contact of an index case of SARS-CoV-2.

- Participants are also excluded if they live alone. This reduces the risk to participants should there be a serious adverse event to Ivermectin/Placebo. Note in this context that Ivermectin has been used in large numbers of people worldwide to treat parasitic diseases for approximately 40 years with a favourable side effect profile in that setting (Crump and Omura 2011 and see also Chaccour et al 2017 who consider Ivermectin pharmacokinetics and pharmacodynamics with respect to reducing malaria transmission) and See Sections 3.3 and 3.3.1 above.

5.2 Justification for Dose

This trial will use a single oral dose of Ivermectin (200ug/kg)/Placebo. This Ivermectin dose is already approved in Australia for another indication (scabies) and has an established favourable safety profile. As the Ivermectin/Placebo is administered early after exposure to an index case infected with SARS-CoV-2, participants are asymptomatic of SARS-CoV-2 at enrolment and have had a negative pharyngeal PCR or RAT for SARS-CoV-2, the Ivermectin/placebo in this trial is being administered to participants whose SARS-CoV-2 viral load is likely to be low.

Arshad et al (2020) considered tissue, rather than just plasma, concentrations of Ivermectin. In their Figure 5, they report the following modelled human tissue Cmax/EC 50 ratios for Ivermectin (which they based upon data from the paper of Caly et al (2020)) and which they modelled based upon a single oral dose of 200ug/kg Ivermectin (see also the Supplementary Table 2 of Arshad et al 2020): lungs 21.02, kidney 16.10, gut 34.39, liver 17.22, brain 32.99, heart 13.92, bone 14.92, muscle 10.14, pancreas 35.87, skin 51.40, spleen 8.6 and thymus 16.32 Furthermore, Schmith et al (2020) cite their reference 4 to state that "ivermectin concentrations remained steady in cattle lungs for 8 days after a single subcutaneous dose" and, in a large animal model (cattle), lung Cmax for Ivermectin was about 2.5 that of plasma Cmax (Lifschitz et al 2000).

More recently, however, two of the investigators (Kylie Wagstaff and David Jans) have demonstrated that in a more appropriate cell line (as opposed to the African Green Monkey fibroblast cell line used in the report of Caly et al (2020)) the IC50 was lower than the data on which Arshad et al (2020) modeled.

In the ICON study, which reported an association between Ivermectin use for those hospitalised with SARS-CoV-2 and lower mortality, the Ivermectin dose was 200ug/kg on Day 1, repeated on Day 8 for those still in hospital (Cepelowicz-Rajter et al 2020a and 2020b). In addition, in a retrospective study from Bangladesh, a single 12mg dose of Ivermectin (which for a 60kg patient is 200ug/kg) given within 24 hours of admission was associated with lower mortality from SARS-CoV-2 (Khan et al 2020a).

Given the above, one might consider an Ivermectin dose of 200ug/kg orally could be therapeutic as a post-exposure prophylaxis. As noted above, Ivermectin may have many potential pathways and mechanisms for efficacy against SARS-CoV-2 (see Section 3.2 above).

Against this are several other publications. Modelling by Schmith et al (2020) considers this dose would be far lower than the in vitro IC50 of Caly et al (2020) and even of the IC50 of the more recent work of Jans and Wagstaff in a different cell line noted above.

Smit et al's (2019) report from IVERMAL demonstrates that even if Ivermectin is given at 600ug/kg for 3 days, most plasma levels are under 100ng/ml. This is in keeping with a mean maximum plasma concentration (Cmax) of approximately 85ng/ml reported by Guzzo et al (2002) on both Days 1 and 7 when Ivermectin 347-594ug/kg (fixed 30mg dose given to participants of different weight) was administered in the fasting state on Days 1,4 and 7.

In keeping with this hypothesis, Krolewiecki et al (2021) present data consistent with a greater efficacy, compared with placebo, against SARS-CoV-2 in hospitalized patients when Ivermectin plasma concentrations are greater than 160ng/ml. In their Discussion, they note: "our results indicate a concentration-dependent antiviral activity of IVM (Ivermectin) in SARS-CoV-2 infected patients treated within 5 days of symptom onset. This statistically significant difference was identified for the relationship between IVM (Ivermectin) plasma concentrations and the primary outcome (Figs. 3 & 4); which confirms previous in vitro activity shown in cell cultures."

However, we consider the in vitro data of Jans and Wagstaff to be proof of concept of a molecular pathway and not prescriptive of a definitive therapeutic plasma concentration. The IC50 in vitro varies with the experimental parameters of each experiment. For example, the published IC50 in Vero Cells (African Green Monkey renal fibroblasts) is much higher than the IC50 when

experiments are performed in a more physiologic human respiratory cell line (personal communication Jans and Wagstaff). In addition, these experiments were conducted at a multiplicity of infection (MOI) that may exceed early exposure but may be more relevant for late established SARS-CoV-2 infection. For these reasons and recognizing that a direct effect of Ivermectin on virus may not be the main or sole pathway of efficacy and given the observational data, of better clinical outcomes with a single dose of 200ug/kg oral Ivermectin in later stages of infection by SARS-CoV-2 and the widespread international safety experience with 200ug/kg Ivermectin, we consider this dose to be justified for a post-exposure prophylaxis RCT.

5.3 End of Study Definition

A participant is considered to have completed the study if he/she has had all follow up to month 6 or has died before then.

6. Study Population

The trial will recruit participants who have, in the preceding 72 hours, had close contact with a person infectious with SARS-CoV-2. Participants must have, since that contact, tested negative for SARS-CoV-2 on polymerase chain reaction (PCR) of pharyngeal swab or on a rapid antigen test (RAT) and be asymptomatic of fever, new cough, sore throat, rhinorrhoea, loss of smell, loss of taste, or more difficulty breathing than usual.

The trial will recruit until 40 participants have converted to a positive PCR or positive TGA-approved RAT for SARS-CoV-2 within 14 days of their contact with an infectious SARS-CoV-2 index case. Such testing in the first 14 days until recently was standard in Australian public health management of such exposed persons, if unvaccinated, and within 7 days if they have received at least 2 doses of vaccination against SARS-CoV-2.

Participants will have a RAT for SARS-CoV-2 immediately prior to study treatment and will be instructed not to take the study treatment if that RAT is positive or inconclusive (for example if the positive control on that test did not work). Participants who are asymptomatic will have a further RAT for SARS-CoV-2 on Day 2, 3, 4 and 5, and on each of days 6 and 14 following close contact (with the day of close contact counted as day 0). If practical and available, which may depend on public health circumstances, they may be asked to have a PCR for SARS-CoV-2 at days 6 and/or 14 following close contact.

Participants will be instructed that they must present for a pharyngeal swab PCR for SARS-COV-2 whenever symptoms of SARS-CoV-2 present (unless public health instruction is to the contrary at the time of their trial participation) If a positive pharyngeal swab PCR for SARS-CoV-2 result is returned no further pharyngeal swab PCR or RAT for SARS-CoV-2 is performed for that participant.

6.1 Inclusion Criteria

Participants are eligible to be included in the study only if all the following criteria apply:

- Aged 18 years to 80 years.
- AND, in the preceding 72 hours, had close contact with a person infectious with SARS-CoV-2.
- AND that contact was in the context of i) a home or ii) an indoor work environment or iii) a family gathering or a social or a religious function or a ceremony each being of less than 30 people.
- AND since that contact, tested negative for SARS-CoV-2 on polymerase chain reaction (PCR) of pharyngeal swab or on a rapid antigen test (RAT).
- AND are asymptomatic of fever, new cough, sore throat, rhinorrhoea, loss of smell, loss of taste, or more difficulty breathing than usual.

With respect to the above Inclusion Criteria, it is prespecified that the Trial Steering Committee (see Section 12.6.2) will regularly consider the trial recruitment experience and whether interval specified above, since potential participant close contact with an index case (currently 72 hours) is too short to provide practical opportunity to recruit sufficient participants to conduct the trial. The Trial Steering Committee may determine to extend that interval in the light of ongoing recruitment experience after the trial opens, balancing the intention to test the Investigational Product early after contact with an index case against the practicality of participant recruitment.

The Trial Steering Committee will review the number of participants who returned a positive pharyngeal PCR or RAT for SARS-CoV-2 after the 200th participant has reached study day 14 and consider whether to alter the Inclusion Criteria in the light of the proportion of those 200 participants who are reaching that end point.

The Trial Steering Committee may also determine to pause and/or resume recruitment and/or change recruitment location (for example in Australia, the State(s) within which recruitment occurs) in response to changes in SARS-CoV-2 incidence.

6.2 Exclusion Criteria

Participants are excluded from the study if any of the following criteria apply:

- Not agreeable to being at home for delivery of the investigational product
- The close contact with an infectious index case of SARS-CoV-2 occurred in a hospital
- Residing outside the current geographic recruitment area
- The index case who has SARS-CoV-2 lives in the same residence as the potential participant.

- Another person who lives in the same residence as the potential participant has returned a positive pharyngeal PCR or a RAT for SARS-CoV-2 in the last 2 weeks.

- Unable to provide the name, address and phone number of the potential participant's general practitioner/primary care physician OR does not have such a general practitioner/primary care physician.

  (NB: The trial gathers data by telephone/email but does not clinically examine or monitor participants. That is done by their general practitioner/primary care physician and/or public health authority See Section 5.1.7 above, Participant Oversight).

- Has not attended a doctor at the practice of the above general practitioner/primary care physician for more than 12 months. (NB Given the pandemic, that attendance may have been by telehealth).

- Lives alone (as potentially at higher risk should there be a serious adverse event).

- Unable to provide the name and phone number of a back-up contact person.
- History of past infection with SARS-CoV-2.
- Use of Ivermectin for any purpose in 5 weeks prior to enrolment.
- Known past allergy or severe adverse reaction to Ivermectin.
- Weight <45kg or > 120kg.
- Pregnant or breast feeding.
- Not willing to refrain from falling pregnant or fathering a child for 6 months after last dose of investigational product.
- Cirrhosis or known decompensated liver disease (Child-Pugh B or C- Refer to Appendix 6).
- Current use, or use within the last 3 months, of the drug amiodarone.
- Current use of any of the following drugs: warfarin, verapamil, diltiazem, quinidine, spironolactone, ciclosporin, tacrolimus, cobicistat, indinavir, ritonavir, didanosine (DDI), ketoconazole, itraconazole, fusidic acid, erythromycin, clarithromycin.
- Past sedation or somnolence from products containing codeine (as Sistonen et al (2012) found this may be associated with a genotype for P-Glycoprotein (which pumps Ivermectin out of the central nervous system), which may be a marker for risk for rare serious adverse effect from Ivermectin, see Edenbridge Investigational Brochure, Section 5.1.1 "Ivermectin metabolism in humans."
- History of residency or travel to loa loa endemic areas ("Angola, Cameroon, Central African Republic, Chad, Democratic Republic of Congo, Ethiopia, Equatorial, Guinea, Gabon, Republic of Congo, Nigeria and Sudan", Chaccour et al 2020b).

- Severe Asthma (as Stromectol product information notes in "Post-marketing Experience" "very rarely,…worsening of bronchial asthma"
- Encephalopathy.
- Head injury requiring medical attention in the last 6 months.
- Concussion within the last 6 months.
- Fit, seizure, stroke, TIA (transient ischaemic attack) or transient global amnesia in the last 6 months.
- History of epilepsy (as Stromectol product information notes in "Post-marketing Experience" "very rarely,…seizures").
- Dementia of any type.
- Not usually fully independent in activities of daily living and self-care, including: washing, toileting, dressing and dental care.
- Inability of participant to communicate to the level necessary to provide verbal or written consent.
- Incarcerated by local, state or federal authorities.
- Conditions which in the opinion of the investigative team would make successful trial completion (including follow up data collection) unlikely, for example including uncontrolled substance use, poorly controlled mental state disorder.
- Unable to advise trial staff of Coronavirus vaccination status including date of administration of last vaccine dose.

  (NB past vaccination against Coronavirus is NOT an exclusion as whilst it may reduce disease severity it may not prevent infection. However, randomization stratifies by Coronavirus vaccination status, knowledge of which is thus a prerequisite for the protocol)
- Already enrolled in another Coronavirus RCT
- In Australia, lack of a valid Medicare Card
- Unable or unwilling to have a RAT upon receipt of investigational product, on Days 1,2, 3, 4 and 5, and on days 6 and 14 following close contact.
- Unable or unwilling to have a pharyngeal swab PCR test for SARS-CoV-2 at 6 and/or 14 days post initial exposure to a close contact if such tests are then practical and available given the then public health circumstances.

6.3 Lifestyle Considerations

6.3.1 Diet and Activity

Participants will not have any dietary or activity restrictions imposed while participating in the clinical

trial.

6.4. Screen Failures

Individuals who do not meet the criteria for participation in this trial (screen failure) may be rescreened if there are changes in circumstance.

7. Study Intervention

Ivermectin (Stromectol), is an approved therapeutic in Australia (Australian Register of Therapeutic Goods (ARTG ID: 181338)).

7.1 Study Intervention(s) Administered

Clinical trial participants will be randomized to Ivermectin or placebo 1:1.

	Ivermectin	Placebo
Dosage formulation	3mg tablets	Tablets
Route of administration	Oral	Oral
Dose	200ug/kg	NA
Dosing Instructions	i. On Day 1, participants will take the dose Ivermectin/Placebo orally with water as soon as possible on receipt of the Ivermectin/Placebo and at least 2 hours after their last meal. They will then wait at least 1 hour before their next meal.	
Description<br>As for Australia, USA, Europe and Israel. May vary with country.	White, round, flat, bevel-edged tablets debossed with “806” on one side and plain on the other side.<br>Tablets come in blister packs (each pack has 20 tablets) in a carton.	White, round, flat, bevel-edged tablets debossed with “806” on one side and plain on the other side.<br>Tablets come in bulk bottles
Storage	Stored below 30 degrees	Stored below 30 degrees
**Packaging and Labelling**	Study Intervention will be provided in packs of 20. These tablets are all in individual blisters with perforations between blisters. Packs will be labelled as required.	Study Intervention will be provided in bulk bottles.
Supplier	Edenbridge Pharmaceuticals LLC Parsippany, NJ 07054, USA	Edenbridge Pharmaceuticals LLC Parsippany, NJ 07054, USA

7.1.1 Preparation/Handling/Storage/Accountability

i. The study will use clinical trials pharmacies. Ivermectin and placebo tablets will be delivered respectively to the clinical trials pharmacy prior to enrolment of the first participant.

ii. The clinical trial pharmacy must confirm appropriate temperature conditions have been maintained during transit for all study intervention received and any discrepancies are reported and resolved before use of the study intervention.

iii. Receipt and dispensing of Ivermectin and placebo will be captured in appropriate pharmacy logs.

iv. The clinical trials pharmacy will dispense investigational product or placebo for individual participants.

v. Following consent, the pharmacy is notified by trial personnel that a participant has been recruited to the study. The pharmacist will randomize using the REDCap database, prepare the participant dosage instruction sheet to accompany the investigational product, and arrange for the investigational product to be delivered to the participant.

7.1.2. Mechanism of Randomization and distribution of Treatment/Placebo

Immediately following consent, trial personnel (of the CRO) will contact the relevant clinical trials pharmacy, forward the electronic prescription, and provide the following participant information:

- name,
- date of birth
- weight (must be >45kg and <121kg).
- **SARS-CoV-2 vaccination status**: unvaccinated, received first vaccination, received second vaccination in the last 10 calendar days, received the second vaccination more than 10 calendar days but less than 6 months prior to Consent, received the second vaccination 6 or more months after consent, received a third vaccination. (If a participant fulfils both the last category and any other category, that participant is allotted to the last category).
- **nature of exposure to index case**: i) a home or ii) an indoor work environment or iii) a family gathering or a social or a religious function or a ceremony each being of less than 30 people.

7.1.3. Pharmacist obtains the randomization outcome

After notification of a participant requiring trial tablets, a clinical trials pharmacist uses the REDCap database for the randomization outcome.

7.1.4 Dispensing investigational product and its delivery to participants

The pharmacist will refer to Appendix 2a to determine the number of required Ivermectin/placebo

tablets for a participant. When dispensing Ivermectin, the pharmacist will need to decant the Ivermectin from the blisters for each participant. The pharmacist will then obtain the number of tablets from that number of tablet blisters (see Section 7.1 above) and will transfer that number of tablets into a bottle with a child proof lid (HDPE bottle is preferred) containing dessicant. Dessicant will not be required if the interval between dispensing and consumption is within 24-48 hours. The bottle will be labelled as required and placed into a bag or box together with an instruction sheet (Appendix 2b), for delivery to the participant. The pharmacist will also include in the dispensing bag or box: a trial card with a clinical contact phone number (Appendix 2c), 4 symptom diary sheets (Appendix 4).

The pharmacist will then store in the pharmacy, in a bag labelled with the participant name, one spare packet of investigational product (Ivermectin/placebo tablets) identical to that dispensed to that participant. This will be retained by the pharmacy until trial close out. It preserves the opportunity for future audit of the correct attribution and dispensing of Ivermectin versus placebo.

Female participants aged under 60 years will also be dispensed a urine pregnancy test kit to be used prior to taking the investigational product as pregnancy is an exclusion criterion. The dosage instruction sheet above will instruct female participants to conduct the urine pregnancy test kit prior to taking tablets and NOT to take any of the tablets if they are pregnant. It will state that if she is pregnant, she must immediately telephone her general practitioner/primary care physician and must immediately telephone their public health authority and report to both that she is a close contact of an index case of SARS-CoV-2 and has just returned a positive urine pregnancy test. The participant will also need to take a photograph of the pregnancy test kit outcome and send it to the CRO personnel via the study email.

Trial participants will each receive rapid antigen test (RAT) kits for SARS-CoV-2. They will be instructed to perform a RAT on receipt of the test kits and not to consume the investigational product if that test returns a positive or inconclusive (for example failure of the positive control) test result. They will be instructed to perform a RAT again on days 1, 2, 3 and 4 after taking the investigational product (with day of investigational product consumption counted as Day 0). The participants will also be instructed to perform a RAT on days 6 and 14 following their close contact with an infectious index case (with day of close contact counted as Day 0) if asymptomatic. The RAT kits being used in Australia may, or may not, be TGA approved. The study participants will be instructed to use a TGA approved RAT at Day 6 if the kits provided for the study are not TGA approved. The participant will be instructed to take a photograph of the RAT testing cassette following each RAT (see Appendix 2b) and send it to the CRO personnel via the study email.

Trial participants are potentially infectious and cannot safely collect trial drug. It must be delivered to their residence. Trial participants will need to be at home until at the time the investigational product is delivered to them. The consenting doctor will write a Medical Certificate if required in order for the participant to be at home to receive the investigational product.

In each country this will be done in accordance with local policies.

In Australia, a courier or taxi service will be responsible for contactless delivery of the investigational

product to the participant.

The procedure in Australia will be as follow:

- Immediately after the pharmacist is notified of the newly enrolled participant, the courier or taxi service will be contacted by trial personnel or Pharmacy and advised of the address of the new trial participant and when the study drug will be ready for collection.
- Either the Pharmacy or NTA personnel will call the participant and confirm the address details and expected time of delivery and instruct that they should first confirm a negative rapid antigen test, and then take all the tablets as per the instruction on the dosage instruction sheet in the delivery box, as long as the RAT is negative, (and that female participants should first use the urine pregnancy test kit and not take the tablets if pregnant). They will be instructed to contact the phone number on the Clinical Contact Card if they have any questions.
- The courier or taxi service or Pharmacy will contact trial personnel to confirm approximate time of delivery of the tablets to the participant.
- The courier or taxi service will dispatch staff to the pharmacy and then to participant's address.
- The courier or taxi service staff will arrive at that address, drop off the tablets, rapid antigen kits and urine pregnancy test kit (the latter for women under age 60) through contactless delivery at the front door. They will take a photo of the drop-off destination.
- Either the courier or taxi service or Pharmacy emails NTA the photo of the drop-off point with confirmation of applicable participant name.

Recording time of drug administration (time of intervention)

Once the photo of the drop-off point with confirmation of applicable participant name has been received at NTA , trial personnel will record date and time of that photo as proxy for date and time of drug administration.

7.1.5. Tablets prepared in series not in parallel

A pharmacist will only dispense investigational product for one participant at a time to minimise the risk of error.

7.1.6. Pharmacy data and treatment/placebo retention

The clinical trial pharmacy will retain the result of treatment allocation in hard copy in addition to the electronic version maintained on REDCap. At the end of the study the CRO will perform drug accountability and confirm correct drug allocation and then close out the Pharmacy.

7.1.7. Distribution of trial tablets and recording of drug administration

See Section 7.1.4 Dispensing investigational product and its delivery to participants above.

7.1.8. No return of tablets or tablet count

There will be no return of (unconsumed) tablets to the clinical trials pharmacy as participants may be infectious with SARS-CoV-2.

7.2. Measures to Minimize Bias: Randomization and Blinding

- This trial is double blinded as the usual medical staff caring for/monitoring the participant, the participants taking the tablets, the CRO and personnel collecting data and the investigators will not know in real time the treatment allocation of any given participant.

- All participants will be assigned to randomized study intervention using the REDCap database.

7.2.1. Unblinding Procedures

In the event of medical emergency, health care workers may contact the investigators to request participant allocation. The site Pharmacist should then be contacted to obtain the treatment allocation. Unblinding may also occur at the request of a Medical Monitor (MM) or a member or the Data Safety and Monitoring Board (DSMB).

7.3. Study Intervention Compliance

Clinical Trials Pharmacy will maintain accountability logs and dispensing logs that will be made available to monitoring staff.

Drug reconciliation at Clinical Trial Pharmacy will occur at the end of the study.

Four hours after delivery of the study drug to the study participant, the participant will be contacted via phone by the CRO to ascertain:

1. Whether all/some of the study drug was taken
2. If not, the reason why
3. Whether any adverse effects were noted

7.4 Concomitant Therapy

The following drugs are exclusions as they are potential strong inhibitors of both cytochrome CYP3A4/5 which metabolises Ivermectin and of P-glycoprotein which transports Ivermectin out of the central nervous system: amiodarone, verapamil, ciclosporin, cobicistat, ritonavir, didanosine, fusidic acid, erythromycin, clarithromycin, ketoconazole and itraconazole (see The Supplementary Appendix of Baudou et al 2020) . The Stromectol Product Information notes post-marketing reports

of changes in International Normalised Ratio (INR). Ongoing use of warfarin is an exclusion criterion.

7.5 Dose Modification

The protocol does not allow for dose modification.

7.6 Intervention after the End of the Study

None

8. Discontinuation of Study Intervention and Participant Discontinuation/Withdrawal

8.1 Discontinuation of Study Intervention

Participants receive first dose Ivermectin 200ug/kg/Placebo on Day 1. There are no further doses. Each female participant is dispensed a urine pregnancy test together with the Ivermectin/placebo tablets and is instructed to perform this prior to taking tablets and NOT to take any tablets if she is pregnant. Each participant is dispensed with a Rapid Antigen Test (RAT) to be performed prior to taking the tablets. If the RAT result is positive or inconclusive, participants are NOT to take any tablets.

8.1.1Temporary Discontinuation

See 8.1 above.

8.2. Participant Discontinuation/Withdrawal from the Study

A participant may withdraw from the study at any time at his/her own request or may be withdrawn at any time at the discretion of the investigator for safety, behavioural, or compliance reasons.

If the participant withdraws consent for disclosure of future information, the sponsor may retain and continue to use any data collected before such a withdrawal of consent.

8.3. Lost to Follow Up

A participant will be considered lost to follow-up if he or she is repeatedly unable to be contacted by the study personnel.

Before a participant is deemed lost to follow up, the CRO, investigator or designee must make every effort to regain contact with the participant (where possible, 3 telephone calls to the participant, 3 telephone calls to the participant’s nominated contact back-up person and 2 telephone calls to the participant’s general practitioner/primary care physician). These contact attempts should be documented in the REDCap database.

9. Study Assessments and Procedures

No physical assessments will be performed by trial personnel during this study (except if they are a participant's usual treating physician and are performing usual care). All data collection will be performed by phone or through electronic communication or by review of hospital, laboratory, specialist or primary care physician files.

9.1. Consent

See Sections 5.1.3-5.1.5 above.

9.2. Data collection

Data will be collected from participants, hospital and laboratory records and specialist and/or primary care physicians records. All data collected will be entered into REDCap by trial personnel.

9.2.1. Screening data

From participant

Identifiers:

- Name
- Sex
- Date of birth (and Age)
- Weight
- Current residence address
- Participant's back-up contact person
    - name,
    - relationship to participant (e.g. sibling, friend),
    - back-up contact person phone number

Inclusion criteria

- Date of close contact with an index case of SARS-CoV-2
- Date of negative pharyngeal swab PCR or negative RAT for SARS-CoV-2
- If PCR was performed
    - copy of notification report of that PCR result for example screen shot photograph of laboratory text message) sent to trial email address
- If RAT was performed:
    - which type of RAT was used (saliva or nasal swab)
    - trade name of the RAT
    - and if available, photograph of RAT test cassette result sent to trial email address
- Symptoms: fever, new cough, sore throat, rhinorrhoea, loss of smell, loss of

taste, more difficulty breathing than usual

- Site of exposure to the close contact: i) a home or ii) an indoor work environment or iii) a family gathering or a social or a religious function or a ceremony each being of less than 30 people.

Exclusion criteria

- Participant declines to be at home when IP is delivered.
- Did the close contact occur in a hospital
- Residing outside the current geographic recruitment area
- Does the index case who has SARS-CoV-2 live in the same residence as the potential participant.?
- Is there anyone living in the same residence as the potential participant who has returned a positive pharyngeal PCR or a RAT for SARS-CoV-2 in the last 2 weeks.
- The potential participant lives alone
- Has a general practitioner/primary care physician
- • The name, address and phone number of that doctor
- Has attended that doctor's practice in the last 12 months (including by telehealth).
- History of past infection with SARS-CoV-2
- Use of Ivermectin for any purpose in 5 weeks prior to enrolment
- Known past allergy or severe adverse reaction to Ivermectin
- Weight <45kg or >120kg (round weight up to the nearest kg if integer weight not given by participant).
- Pregnant or breast feeding
- Not willing to refrain from falling pregnant or fathering a child for 6 months after last dose of investigational product
- Cirrhosis or known decompensated liver disease (Child-Pugh B or C – Refer to Appendix 6)
- Current use, or use within the last 3 months, of the drug amiodarone.
- Current use of any of the following drugs: warfarin, verapamil, diltiazem, quinidine, spironolactone, ciclosporin, tacrolimus, cobicistat, indinavir, ritonavir, didanosine (DDI), ketoconazole, itraconazole, fusidic acid, erythromycin, clarithromycin.
- Past sedation or somnolence from products containing codeine
- History of residency or travel to loa loa endemic areas ("Angola, Cameroon, Central African Republic, Chad, Democratic Republic of Congo, Ethiopia,

Equatorial, Guinea, Gabon, Republic of Congo, Nigeria and Sudan”, Chaccour et al 2020b).

- Severe Asthma Encephalopathy
- Head injury requiring medical attention in the last 6 months
- Concussion within the last 6 months
- Fit, seizure, stroke, TIA (transient ischaemic attack) or transient global amnesiain the last 6 months.
- History of epilepsy
- Dementia of any type
- Not usually fully independent in activities of daily living and self-care including: washing, toileting, dressing and dental care.
- Inability of participant to communicate to the level necessary to provide verbal or written consent.
- Incarcerated by local, state or federal authorities
- Conditions which in the opinion of the investigative team would make successful trial completion (including follow up data collection) unlikely, for example including uncontrolled substance use, poorly controlled mental state disorder.
- Coronavirus vaccination status including type of vaccine, number of times vaccine has been administered and date of administration of last vaccine dose.
- Already enrolled in another Coronavirus RCT
- In Australia, lack of a valid Medicare Card
- Unable or unwilling to have a RAT upon receipt of investigational product, on Days 1, 2, 3, 4 and 5, and on days 6 and 14 following close contact.
- Unable or unwilling to have a pharyngeal swab PCR test for SARS-CoV-2 at 6 and/or 14 days post initial exposure to a close contact if such tests are then practical and available given the then public health circumstances.

If potential participant fails screening:

- “Would the person be happy for NTA to contact them directly regarding other Coronavirus trials?” (Yes/No)

9.2.2. Baseline data on Day 1= Day of Treatment

<u>From participant</u>

<u>Target time of collection:</u> As soon as possible after consent.

- Height (Self-reported)
- Drugs charted at time of enrolment (names, strengths, doses)

- Non-pharmaceuticals charted at time of enrolment e.g. vitamins (names, strengths, doses)
- Health summary including
  - Smoking status (current, former, never)
  - Alcohol consumption (grams per week, in Australia one standard drink will be considered 10 grams)
  - Comorbidities (specifically: Intercurrent Diabetes (Type 1, 2 unknown), Heart Disease (and if Yes :then History of heart attack (yes/no), Angina (yes/no), Coronary Artery Bypass Surgery (yes/no), Coronary Stent (yes/no)), Lung disease (and if Yes then: Emphysema/COPD (yes/no), Asthma (yes/no))…., Hypertension, Kidney disease (and if Yes then: has dialysis (yes/no), Renal Transplant (yes/no)), cancer (which organ), history of stroke)

- Is there anyone else in your residence who is also a close contact of an index case of SARS-CoV-2? (Record Yes/No and number of such people)

- Is there anyone in your residence who has been diagnosed in the last 2 weeks with SARS-CoV-2 (Record Yes/No and number of such people and when each was diagnosed as being infected).

- Are you at isolating at home?

9.2.3. Data collection 4 hours after Investigational product delivered

Rapid antigen test (RAT)

-
- Did you perform the rapid antigen test (yes/no)
- If not, why not?
- And if not performed, participant is advised to immediately perform that RAT and email a photograph of the result (see Appendix 2b).
- What was the result of the RAT?
- Did you email a photograph of the result?

Urine pregnancy test

- Did you perform the pregnancy test?
- If not performed and participant is a woman under age 60, participant is instructed to perform it immediately and email a photograph of the result
- What was the result?
- Did you email a photograph of the result?

Investigational product

- Did you take the Investigational Product (Ivermectin/Placebo tablets) as

directed? (Yes/No)
- If not, why not?
- If not taken as directed, what did you do instead?

Adverse reaction to Ivermectin/Placebo
Any potential side effects of Ivermectin/Placebo
- Yes (specify)/No
- Date of potential side effect
- Severity (as mild, moderate, or severe)
- Response and/or treatment (e.g. given acetominophen)

Symptoms of SARS-CoV-2

Participants are reminded to complete the symptom diary (see Appendix 4) starting the calendar day Ivermectin/Placebo was taken

Participants are reminded:
If you develop any symptom, if you feel it is an emergency call an ambulance immediately (insert country specific ambulance phone number).

If you don't feel it is an emergency, you must telephone your usual general practitioner and remind the doctor you are a close contact of a person with Coronavirus.

You must also telephone your local public health agency on this 24 hour telephone number (insert country specific number).

Participants are reminded to keep the trial clinical contact card with them for the duration of the study and that it has a phone number which participants, doctors or other health workers can use if they wish to speak with a trial doctor.

Experienced symptoms of SARS-CoV-2 between enrolment (consent) and taking the tablets
- Symptoms to check: fever, new cough, sore throat, rhinorrhoea, loss of smell, loss of taste, more difficulty breathing than usual

Experienced symptoms of SARS-CoV-2 between taking the tablets and the 4 hour phone call
- Symptoms to check: fever, new cough, sore throat, rhinorrhoea, loss of smell, loss of taste, more difficulty breathing than usual

9.2.4. Data collection on Days 8, 15, 22, 29

If symptoms consistent with SARS-CoV-2 are experienced, participant must have a pharyngeal swab

PCR for SARS-COV-2 (unless public health instructions preclude this at the time participant is in the trial)

From participant

Endpoint data

Day 8 data collection only:

- Did you do a RAT on day 6 following your close contact (counting the day of the close contact as day 0)?
- If not, why not?
- If not, participant is instructed to do one immediately unless has already returned a positive PCR result for SARS-CoV-2
- What was the result of the RAT?
- Have you emailed the photograph of the result?

Day 15 data collection only:

- Did you do a RAT on day 14 following your close contact (counting the day of the close contact as day 0)?
- If not, why not?
- If not, participant is instructed to do one immediately unless has already returned a positive PCR result for SARS-CoV-2
- What was the result of the RAT?
- Have you emailed the photograph of the result?

The following is for data collection on Days 8,15,22 and 29

Participants are reminded to present for PCR for SARS-CoV-2 if symptoms develop (unless public health instruction is to the contrary at the time of their trial participation)

Tested for SARS-CoV-2 by PCR in the last week

- Yes/No

If yes, then for *each* of those tests

Tested positive for SARS-CoV-2 by PCR

- Yes/No

If yes (tested positive), has the participant telephoned

1) the public health authority to confirm they are aware of that

result and to ask what management is required
2) his/her GP to confirm the GP is aware of that result and to discuss management.

- For each (positive and negative test) Date of test
- For each (positive and negative test) Name of testing Laboratory

- In addition, for *each* test, from participant or from testing laboratory:
- Copy of negative PCR test result (for example screen shot photograph of laboratory text message, sent to trial email address)
- Copy of positive PCR test result (from example, electronic copy sent to trial email address from testing laboratory)

<u>Tested for SARS-CoV-2 by RAT in the last week</u>

- Yes/No

<u>If yes, then for *each* of those tests</u>

<u>Tested positive for SARS-CoV-2 by RAT</u>

- Yes/No

<u>If yes (tested positive)</u>, has the participant telephoned
1) the public health authority to confirm they are aware of that result and to ask what management is required
2) his/her GP to confirm the GP is aware of that result and to discuss management.

- For each (positive and negative test) Date of test
- For each (positive and negative test) Name of testing location, type of test (saliva or nasal swab) and name of test kit

- In addition, for *each* test, from participant or from testing location:
- Copy of negative RAT test result (for example screen shot photograph of laboratory text message, sent to trial email address)
- Copy of positive RAT test result (from example, electronic copy sent to trial email address from testing laboratory)

<u>Experienced symptoms of SARS-CoV-2 in the last week</u>

Participants will be asked to keep a symptom diary (see Appendix 4) and the following data will be obtained:

- Fever
- New cough
- Sore throat
- Rhinorrhoea
- Loss of smell
- Loss of taste

- More difficulty breathing than usual.

If participant experienced any of the above symptoms in the last week

- Did you report those symptoms by telephone to your general practitioner/primary care physician? (Yes/No)
- Did you report those symptoms by telephone to your public health authority monitoring your care? (Yes/No)
- If No to either of the above, the participant is instructed to make such a report by telephone immediately and reminded that they are under the care of the above practitioners (see Section 5.1.7 above).

Participants are reminded:
If you develop any symptom, if you feel it is an emergency call an ambulance immediately on (insert country specific ambulance phone number).

If you don't feel it is an emergency, you must telephone your usual general practitioner and remind the doctor you are a close contact of a person with Coronavirus.

You must also telephone your local public health agency on this 24 hour telephone number (insert country specific number).

<u>Presentation to hospital and/or acute hospital care and/or to outpatient care under hospital supervision in the last week</u>

- Yes/No
- Dates of presentation/ongoing care
- Hospital name
- Treating Doctor
- Awaiting a rehabilitation or non-acute care facility bed (date from which waiting)

If discharged following hospital admission

- Date of discharge from hospital
- Discharge destination: (record: Home, Rehabilitation Facility, Aged Care Facility or Other Institution)

Other data

<u>Pharmacotherapy</u>

- Drugs charted (names, strengths, doses)
- Non-pharmaceuticals charted e.g. vitamins (names, strengths, doses)

- Have you received any treatment against Coronavirus in the last week (name, dose and date of therapy)?

Adverse reaction to Ivermectin/Placebo in the last week

- Any potential side effects of Ivermectin/Placebo since last data collection point
    - Yes (specify)/No
    - Date of potential side effect
    - Severity (as mild, moderate, or severe)
    - Response and/or treatment (e.g. given acetominophen)

Isolation/Circumstance of Residence in the last week

- In the last week, which days were you in isolation?
- In the last week, has there been anyone else in your residence who is also a close contact of an index case of SARS-CoV-2 (Record Yes/No and number of such people and which days each was present in the residence)
- Has there been anyone in your residence who has been diagnosed in the last week with SARS-CoV-2 (Record Yes/No and number of such people and when (date) each was diagnosed as being infected with SARS-CoV-2)

9.2.5. Months 2,3,4,5,6

From participant

(or back-up contact or general practitioner/primary care physician if participant has passed away)

Death since last data collection (Yes/No)

- Date of death
- Cause of death
- Death determined by a site investigator as due to SARS-CoV-2 (Yes/No)

Hospitalised since last data collection (Yes/No)

- Date of admission
- Hospital name

Any potential side effects of Ivermectin/Placebo since last data collection point

- Yes (specify)/No
- Date of potential side effect
- Severity (as mild, moderate, or severe)
- Response and/or treatment (e.g. given acetominophen)

9.3. Efficacy Assessments

Efficacy will be assessed by the primary and secondary endpoints.

9.4. Safety Assessments

Safety Assessments will be conducted by asking about symptoms and potential adverse events and will be captured in REDCap.

9.4.1. Physical Examinations

Physical Examinations will not be conducted specifically for the study as participants may be infectious with SARS-CoV-2 infection.

9.4.2. Vital Signs

Monitoring of vital signs will be by the participant's usual clinical carers as part of their routine clinical care.

9.4.3. Clinical Safety Laboratory Assessments

There are no clinical safety assessments for this study as participants may be infectious with SARS-CoV-2. Laboratory monitoring is done by the participants' usual caring doctors as part of their routine clinical care.

9.5. Adverse Events and Serious Adverse Events

An Adverse Event (AE) is any untoward medical occurrence in a participant temporally associated with the use of the investigational product, whether considered related to the investigational product or not. An AE can therefore be any unfavourable and unintended sign (including an abnormal laboratory finding), symptom, or disease (new or exacerbated) temporally associated with the use of the product, whether considered related to the product or not. This also includes abuse or misuse.

AEs will be reported by a participant (or, when appropriate, by a surrogate) or a investigator.

In this trial, AE and SAE are detected, documented, and recorded during CRO or investigator follow up of participants. The CRO will bring AEs and SAEs to the attention of the MM who will help classify each event as an AE or SAE and advise on follow up. The CRO will pass the advice of the MM as appropriate to the investigators and/or data safety monitoring board (DSMB) and will advise the sponsor of AEs and SAEs. The sponsor or CRO will also notify the Regulatory agency/ies and/or HREC/IRB as required of AEs and/or SAEs.

The Medical Monitor will make an assessment of intensity for each AE and SAE reported during the study and assign it to one of the following categories:

• **Mild:** An event that is easily tolerated by the participant, causing minimal discomfort and not interfering with everyday activities.

• **Moderate:** An event that causes sufficient discomfort to interfere with normal everyday activities.

• **Severe:** An event that prevents normal everyday activities. An AE that is assessed as severe should not be confused with an SAE. Severe is a category utilized for rating the intensity of an event; and both AEs and SAEs can be assessed as severe.

9.5.1. Time Period and Frequency for Collecting AE and SAE Information

All SAEs will be collected from the start of intervention until 6 months.

Medical occurrences that begin before the start of study intervention but after obtaining informed consent will be recorded on the Medical History/Current Medical Conditions section of the case report form (eCRF), not the AE section.

All SAEs will be recorded and reported via trial personnel to the MM within 24 hours.

Investigators are not obligated to actively seek AE or SAE after conclusion of the study participation. However, if the investigator learns of any SAE, including a death, at any time after a participant has been discharged from the study, and he/she considers the event to be reasonably related to the study intervention or study participation, the investigator must promptly notify the sponsor.

9.5.2. Method of Detecting AEs and SAEs

Care will be taken not to introduce bias when detecting AEs and/or SAEs. Open-ended and non-leading verbal questioning will be used.

Investigators are not required to report SAEs which are consistent with SARS-CoV-2 and expected disease progression including:

- venous thromboembolism.
- chest pain.
- shortness of breath.
- fever or other features judged clinically to be consistent with SARS-CoV-2 disease progression.

9.5.3. Follow-up of SAEs

All SAEs will be followed until resolution, stabilization, the event is otherwise explained, participant has completed all study follow ups, death, or the participant is lost to follow-up.

9.5.4. Regulatory Reporting Requirements for SAEs

Suspected unexpected serious adverse reactions (SUSARs) will be reported to the reviewing HREC/IRB and regulatory agency/ies according to the country requirements.

9.5.5. Disease-Related Events and/or Disease-Related Outcomes Not Qualifying as SAEs

SARS-CoV-2 infection affects different people in different ways. Most infected people will develop mild to moderate illness and recover without hospitalization.

9.5.5.1. Most common symptoms:

- fever
- new dry cough
- tiredness

9.5.5.2. Less common symptoms:

- aches and pains
- sore throat
- diarrhoea
- conjunctivitis
- headache
- loss of taste
- loss of smell

9.5.5.3. Serious symptoms:

- difficulty breathing or shortness of breath

Because these events are typically associated with the disease under study, they will not be reported according to the standard process for expedited reporting of SAEs even though the event may meet the definition of a SAE. These events will be recorded in the participant's CRF

NOTE: However, if either of the following conditions applies, then the event must be recorded and reported as an SAE (instead of a DRE):

- *The event is, in an investigator's or a MM's opinion, of greater intensity, frequency, or duration than expected for the individual participant.*

  OR

- *An investigator or a MM considers that there is a reasonable possibility that the event was related to study intervention.*

9.6. Treatment of Overdose

In this study the possibility of overdose is small since participants will only receive standard 200ug/kg oral Ivermectin or placebo at a single timepoint.

"There are reports of accidental overdosing of Ivermectin, but no fatalities have been attributable to Ivermectin overdosing.

In significant accidental intoxication with unknown quantities of a veterinary formulation, symptoms

have resembled those seen in animal toxicology studies, which were chiefly rash, contact dermatitis, oedema, headache, dizziness, asthenia, nausea, vomiting, diarrhoea, mydriasis, somnolence, depressed motor activity, tremors and ataxia. Other adverse effects that have been reported include seizure, dyspnoea, abdominal pain, paraesthesia and urticaria.

In case of accidental poisoning, supportive therapy, if indicated, should include parenteral fluids and electrolytes, respiratory support (oxygen and mechanical ventilation if necessary) and pressor agents if clinically significant hypotension is present. Induction of emesis and/or gastric lavage as soon as possible, followed by purgatives and other routine antipoison measures may be indicated if needed to prevent absorption of ingested material. Although data are not available for man, it would appear advisable to avoid GABA-agonistic drugs in the treatment of accidental Ivermectin intoxication.

In a study in which healthy volunteers were orally administered up to 2000 µg/kg Ivermectin in a fasted state or up to 600 µg/kg ivermectin following a high-fat (48.6 g of fat) meal, there were no indications of central nervous system toxicity observed at any dose irrespective of food intake." (Stromectol Product Information – Appendix 1).

10. Data management, monitoring and storage

10.1. REDCap data entry and backup

Data entry and storage will be on password protected computers. The data will be regularly and routinely backed up, have cybersecurity provision, and the CRO will monitor ongoing cybersecurity risk.

Trial personnel will be responsible for entering participant data into REDCap. Required data is listed in Sections 9.1, 9.2 and 9.4.

The CRO will monitor the risk of missing data and ensure data is collected according to the protocol, entered onto the database through case report forms (CRF) at the earliest opportunity and that missing data is promptly chased.

Access to data during the trial will be permitted to: staff of the CRO, principal investigators, investigators, trial statisticians, members of the trial Data Safety Monitoring Board, Medical Monitors and/or trial steering committee (see below) and/or staff working for or under the direction of or volunteering with the above for the purpose of the trial from time to time according to their roles. The CRO will be responsible for provision of such data access.

Study staff employed by the CRO may work from wherever that organization deems safe and appropriate for this trial, especially in the context of a pandemic lockdown and that organization will be responsible for monitoring to ensure that with this approach participant privacy and confidentiality is maintained and that data entry is prompt and accurate in that work structure.

The CRO will supply the raw data in tabulated computer file form (for example Excel files) to a study statistician(s) and/or the co-ordinating principal investigator and/or other members of the trial steering committee and/or the data safety monitoring board as and when required.

Consent will be obtained for indefinite data storage. This is because the SARS-CoV-2 virus has only recently been described as a cause of human illness and this and the uncertain future of the SARS-CoV-2 pandemic, combined with international intense research into SARS-CoV-2 pandemic mean

there may be potential for the data to contribute to future SARS-CoV-2 research or other research in a manner that may be unforeseen at trial inception.

When the investigators have completed all trial analyses and reporting, a copy of the computer database and an archive of any paper records (such as faxed discharge summaries), together with the pharmacy data will be stored indefinitely (and for a minimum 15 years) as archived data in a secure manner determined at that time, in consultation with the sponsor, depending on the technology and infrastructure then available to the sponsor.

10.2. Identification or De-identification of data

Participants will be advised in the PICF that each participant will be allocated a unique study number.

Participants will also be advised that they are consenting, for the purposes of this research trial that: data stored and analysed by the researchers will be stored as identified data. They will be advised that this is essential for study functioning, for example to be able to confidently deliver study drug to the correct person or to communicate with other health providers such as primary care physicians, hospitals and SARS-CoV-2 testing centres. It is also important to confirm integrity of study data if there are missing or outlying data to be chased. This is in keeping with standard clinical practice whereby, particularly in this pandemic, health professionals are texting, emailing and generally corresponding electronically about patients with identifiable data in patient letters and are accessing databases online (such as of pathology or radiology providers) for routine clinical care which identify patients by name and date of birth.

Data sharing for presentation or publication will be de-identified and presented in summary form wherever possible. No individually identified information will be released other than as required for the health care of a given individual participant. Sharing of non-identifiable data (such as to support collaborative meta-analysis within ethics-approved projects or for individual pooled data analysis with ethics approval) will be encouraged to maximize scientific value accruing from participation.

Thus, participants are being openly and prospectively advised in the PICF that data will be stored in an identifiable manner (although only publicly shared, presented or published in a de-identifiable manner). They are advised that this is a voluntary study. They have the option not to participate in the study. However, if they choose to participate, they are being given the research respect to autonomously and voluntarily choose to do so knowing data will be handled as above. The above approach is in keeping with past HREC approved research of the investigators including, for example the Australian study TPEP-Corona-1, see Australia New Zealand Clinical Trials Registry (ACTR) ACTRN12620000982910.

10.2.1 Sponsor access to de-identified participant level data

Participants will be advised in the PICF that, for the purposes of seeking regulatory approval for use of Ivermectin should it demonstrate benefit in this and/or related studies, the RCT global sponsor will have access to de-identified participant level data. This access will not occur before the Trial Steering Committee has determined that statistical analysis of the primary endpoint has been performed.

11. Statistical Considerations

The trial aims to recruit until 40 participants have a positive pharyngeal swab PCR or a positive RAT for SARS-CoV-2 within 14 days of exposure to an infectious index case.

11.1. Power and sample size

The trial has over 90% power to detect (2-sided testing with an alpha of 0.05) a 66% reduction (Ivermectin versus Placebo) of the incidence of a positive pharyngeal swab PCR or a positive RAT for SARS-CoV-2 at or before Day 14 after close contact with an index case of SARS-CoV-2.

This is a pilot trial. The number of events was chosen to be able to detect a large clinical effect, for example of a magnitude in keeping with the point estimates of an Ivermectin prophylaxis effect in meta-analyses above, and to provide data which may be used for power calculations for future larger trials should the effect of Ivermectin be less.

In an unvaccinated Spanish population, approximately 18% of those who had negative pharyngeal PCR for SARS-CoV-2 were reported to develop a positive pharyngeal PCR for SARS-CoV-2 during the trial (Mitja et al 2020). The investigators are unaware of any published Australian RCT data regarding percentage conversion to a positive pharyngeal PCR for SARS-CoV-2 after exposure to an infectious index case. Their own observations based on data reported in the media regarding SARS-CoV-2 chains of transmission suggest approximately 10% of those who are primary close contacts in the circumstances which are inclusion criteria for this RCT, namely of i) a home or ii) an indoor work environment or iii) a family gathering or a social or a religious function or a ceremony each being of less than 30 people, will develop a positive pharyngeal PCR for SARS-CoV-2.

On this basis, at least 400 participants would need to be recruited for at least 40 participants to develop a positive pharyngeal PCR following their close contact exposure. The trial will cease recruitment when 40 participants have returned a positive result on either a pharyngeal swab PCR or a RAT for SARS-CoV-2. In order to give greater confidence for achieving 40 such participants, given the uncertainty of the percent of positive contact who will develop a positive pharyngeal PCR or RAT, this pilot trial is resourced to recruit up to 1000 participants.

This pilot trial is collecting data on vaccination status. It may thus develop data regarding the percentage of close contacts who convert to a positive pharyngeal PCR or RAT in a real-world vaccinated population. Such data could be used for the design of future larger trials.

11.2. Basic description of participants

There will be descriptive reporting of participants as a whole and according to treatment cohort.

11.2.1. For the primary endpoints

If 200ug/kg oral Ivermectin has no benefit for post-exposure prophylaxis within 72 hours of close contact with an infectious index case of SARS-CoV-2, then, one would expect; that of the 40 participants who convert to a positive pharyngeal swab PCR or RAT for SARS-CoV-2, approximately half will have received Ivermectin and approximately half will have received placebo.

The proportion of those 40 participants who convert to a positive pharyngeal swab PCR or RAT for SARS-CoV-2, who received Ivermectin, will thus be tested as being different from a proportion of 0.5.

11.2.2. For the secondary endpoints

Amongst the 40 participants who convert to a positive pharyngeal swab PCR or positive TGA-approved RAT for SARS-CoV-2, for the secondary endpoints of:

- Days alive free of symptoms of SARS-CoV-2 at 14 days.
- Days alive free of symptoms of SARS-CoV-2 at 28 days
- Days alive free of presentation to hospital and/or acute hospital care and/or to outpatient care under hospital supervision at Day 28.
- Time from exposure to an index case of SARS-CoV-2 to a positive pharyngeal swab PCR or positive TGA-approved RAT for SARS-CoV-2.

If the data are normally distributed, then mean number of days (for those who received Ivermectin) will be tested for a difference from the mean number of days (for those who received Placebo) by 2 sample T-test. If the data are not normally distributed, then respective medians will be compared by Mann-Whitney analysis.

11.2.3. General statistical points

The primary analysis will be an intention-to-treat. For this analysis, all participants who consumed any trial tablet will be included.

P-values will be reported, and point estimates and 95% confidence intervals of parameters will be provided.

11.2.4. Prespecified subgroups

<u>Country</u>
All analyses will be performed for all participants (adjusting for country of treatment in adjusted analyses) and separately according to country of treatment.

<u>Isolation and presence in isolation of others who have SARS-CoV-2 or are themselves isolating as close contacts of an index case of SARS-CoV-2</u>

Analyses will be performed for all participants, adjusted for whether the participant was in isolation

and if so, whether isolated in the presence or absence of others, who either developed intercurrent SARS-CoV-2 or who were themselves in isolation because they were close contacts of an index case of SARS-CoV-2 (which may or may not be the same index case as relates to that participant).

Analyses will also be performed separately, according to whether participants were in isolation, and if so, whether isolated in the presence or absence of others, who either developed intercurrent SARS-CoV-2 or who were themselves in isolation because they were close contacts of an index case of SARS-CoV-2 (which may or may not be the same index case as relates to that participant).

11.2.5. Further comment on study design: confounding from secondary exposure to SARS-CoV-2 whilst in isolation

A cluster randomized design, randomizing by residence of isolation, with all residents living at the same address randomized to the same treatment (Ivermectin or placebo), was considered as a possible design to reduce confounding from secondary exposure to SARS-CoV-2 whilst in isolation; from others living at the same residence who may have been exposed to the same or a different index case as the participant.

However, in this pilot trial, such an approach was not adopted including for the following reasons. Clustering would not remove the risk of secondary exposure if the residence included people who were ineligible for the trial (such as children or pregnant women) or who were eligible but declined to participate. Recruiting only from residences in which every person was eligible and gave consent to participate would overcome that confounding but would reduce the practicality of recruitment. This is both because there would be fewer such residences available to the study and because of the logistic difficulty of all participants in the same residence being contacted and consented within 72 hours of contact with an index case and having also returned a negative pharyngeal PCR swab or a negative RAT in that timeframe, as required by the Inclusion Criteria. Furthermore, clusters themselves would be heterogeneous with respect to intra-residence risk of secondary SARS-CoV-2 exposure, for example due to differing residence ventilation and differing intimacy amongst residents at different addresses.

Another approach considered was a “ring” cluster randomized design, based on clustering around each index case and randomizing each cluster to Ivermectin or placebo (Mitja et al 2021). However, these clusters may have heterogeneity from differing viral load of each index case and this design may have the same limitations as noted above of a cluster randomized design based on isolation residence.

The current trial’s response to the possibility of confounding from secondary exposure to SARS-CoV-2 includes the following. It will exclude potential participants if the index case lives in the same residence as the potential participant. It will exclude potential participants if the potential participant lives in the same residence as someone who returned a positive pharyngeal swab PCR or positive TGA-approved RAT for SARS-CoV-2 in the last 14 days. It will prospectively collect data noting whether the participant is isolating and if so, whether anyone else living with a participant is themselves in isolation after contact with an index case of SARS-CoV-2 or returns a positive pharyngeal swab PCR or positive TGA-approved RAT for SARS-CoV-2 during the period of

participant follow up. It includes a prespecified plan to analyse data separately according to whether participants were in isolation and if so, in the presence or absence of others, who were themselves in isolation because they were close contacts of an index case of SARS-CoV-2 or who developed intercurrent SARS-CoV-2 infection. It includes a secondary endpoint of time from exposure to an index case of SARS-CoV-2 to a positive pharyngeal swab PCR or positive TGA-approved RAT for SARS-CoV-2. If Ivermectin protects against infection from an index case but those randomized to Ivermectin become infected from a confounding secondary exposure thereafter, there should be a longer time to positive pharyngeal swab PCR or positive TGA-approved RAT for SARS-CoV-2 amongst those who received Ivermectin compared with those who received placebo. However, it is acknowledged that other variables affecting incubation time may reduce the sensitivity of this endpoint.

With the above responses to potential confounding from secondary exposure to SARS-CoV-2, the design adopted for this pilot trial, of randomizing individuals and continuing until 40 events occur, was deemed most practical. A different trial design could be considered for trials that may follow consequent upon the outcome data of this trial.

11.2.6 Sensitivity analyses

Participants who did not take their investigational product according to instruction and/or those whose administered dose was <200ug/kg (see Appendix 3: Table indicating actual dose (ug/kg) of Ivermectin) will be excluded and the above analyses will be repeated.

Participants who experienced symptoms of infection with SARS-CoV-2 between enrolment (Consent) and taking their investigational product tablets and/or who experienced symptoms of infection with SARS-CoV-2 within the first 4 hours after taking their investigational product tablets and/or who were asymptomatic but returned a positive or an inconclusive result on a rapid antigen test for SARS-CoV-2 prior to or within 4 hours after taking the investigational product will be excluded and the above analyses will be repeated.

11.3. Interim Analyses

In order not to delay analysis and urgent public health information, in the context of the SARS-CoV-2 pandemic, analysis may proceed immediately after the 40th participant converts to a positive pharyngeal swab PCR or positive TGA-approved RAT for SARS-CoV-2, (or after stopping by the Data Safety Monitoring Board) using the data available at that point, that is, before final trial data lock. This timepoint corresponds to the last participant reaching the time of the primary endpoint determination. Analyses (such as longer term follow up for adverse events) may be repeated when further data become available.

11.4 Additional endpoints after the 40th participant converts to a positive pharyngeal swab PCR or positive TGA-approved RAT for SARS-CoV-2.

The trial ceases recruitment after a 40th participant converts to a positive pharyngeal swab PCR or positive TGA-approved RAT for SARS-CoV-2. Whilst the interim analyses (Section 11.3 above) occur after the 40th participant converts to a positive pharyngeal swab PCR or positive TGA-approved

RAT for SARS-CoV-2, data collection will still continue on all participants. Further participants, who were already recruited to the trial, may later convert to a positive pharyngeal swab PCR or positive TGA-approved RAT for SARS-CoV-2. As all participants recruited will be eligible for the analyses according to the approaches in the subsections of Section 11, analyses subsequent to the interim analyses could include more than 40 participants who converted to a positive pharyngeal swab PCR or positive TGA-approved RAT for SARS-CoV-2.

12. Regulatory, Ethical, and Study Oversight Considerations

12.1. Human Research Ethics Committee/Institutional Review Board Approval

The protocol will be submitted for approval to the appropriate HREC/IRB. Prior to initiation of the study, written HREC/IRB approval of the protocol and study PICF/ICF must be obtained. This approval letter will identify the study PICF/ICF by version and date and the study protocol by protocol number, title and date. The sponsor or designee will receive all the documentation needed for submitting the present protocol to the HREC/IRB. The composition of the HREC/IRB will also be provided to the sponsor or designee. If approval is suspended or terminated by the HREC/IRB, the investigator will notify the sponsor immediately.

It is the responsibility of the sponsor or designee to report study progress to the HREC/IRB as required or at intervals not greater than 1 year.

The sponsor or designee will be responsible for reporting any SAEs to the HREC/IRB as soon as possible, and in accordance with the guidelines of the HREC/IRB.

12.2. Regulatory and Ethical Consideration

Relevant study documentation will be submitted to the regulatory authorities of the participating countries, according to local/national requirements, for review and approval before the beginning of the study in their jurisdiction. On completion of the study at a site, the regulatory authorities will be notified according to local/national requirements that the study has ended at that site.

The sponsor, CRO and investigators will ensure that this study is conducted in full compliance with the protocol, the Declaration of Helsinki, the ICH GCP guidelines and according to: Federal Drug Administration (FDA) regulations or, Therapeutic Goods Administration (TGA) regulations and/or all applicable local laws and regulations. Compliance with these standards provides assurance that the rights, safety, and well-being of patients are protected.

In agreeing to the provisions of the protocol, these responsibilities are accepted by the investigator.

The local sponsor or designee will be responsible for the following:

- Providing written summaries of the status of the study to the IRB/IEC annually or more frequently in accordance with the requirements, policies, and procedures established by the IRB/IEC
- Notifying the IRB/IEC of SAEs or other significant safety findings as required by IRB/IEC procedures

12.3. Financial Disclosure

Financial Disclosure collection is not applicable for this study.

12.4. Informed Consent Process

The process of obtaining informed consent must be in accordance with applicable regulatory requirement(s) and must adhere to ICH GCP guidelines.

The investigator is responsible for ensuring that no participant undergoes any study related activity beyond the screening stage before that participant has given written or verbal informed consent to participate in the study.

The investigator or designated personnel (who may be functioning under the direction of the CRO) will inform the participant of the objectives, methods, anticipated benefits and potential risks and inconveniences of the study. The participant should be given every opportunity to ask for clarification of any points s/he does not understand and, if necessary, ask for more information. At the end of the interview, the participant will be given sufficient time to consider the study.

It should be emphasised that the participant may refuse to enter the study or to withdraw from the study at any time, without consequences for their further care or penalty or loss of benefits to which the participant is otherwise entitled. Participants who refuse to give or who withdraw written informed consent should not be included or continue in the study.

If new information becomes available that may be relevant to the participant's willingness to continue participation in the study, a new PICF/ICF will be approved by the HREC/IRB (and regulatory authorities, if required). Participants will be informed about this new information and reconsent will be obtained.

A copy of the ICF must be provided to the participant but this may be an electronic copy given considerations of isolation and infection control with SARS-CoV-2.

12.5. Data Protection

Participants will be assigned a unique identifier by the database.

Database access will be password protected.

The REDCap database will be stored on a server which is behind a firewall and regularly backed up.

The participant must be informed that his/her trial records may be examined by authorized personnel appointed by the sponsor, by appropriate IRB/IEC members, and by inspectors from regulatory authorities.

12.6. Committees Structure

This study will have oversight by two committees each with a very different brief.

12.6.1. Data Safety Monitoring Board

The investigators are seeking to establish a fully independent Data Safety Monitoring Board (DSMB) (National Health and Medical Research Council (2018), Data Safety Monitoring Boards (DSMBs))

of suitably qualified and willing people. However, if this is not possible in the timeframe necessitated by the present public health circumstance and would delay trial initiation, the investigators may invite people to serve on the DMSB who have some affiliation with the investigators (for example from the same institution) as is permitted by the National Health and Medical Research Council (2018), Data Safety Monitoring Boards (DSMBs)) publication.

The DSMB will be constituted of at least 3 people, one of whom will chair the committee. The chair should have previous experience on a DSMB. At least one will be a statistician with clinical trials' experience (who will not be a statistician otherwise engaged for this trial) and at least one will be an infectious diseases physician. None will be investigators of this trial. There will be an additional member from each country outside of Australia in which the trial recruits.

The DSMB may meet by computer video conference. Meetings will be organized by the CRO to occur monthly.

The DSMB will examine the data after 20 participants have converted to a positive pharyngeal swab PCR or positive TGA-approved RAT within 14 days of close contact with an infectious case of SARS-CoV-2.

If at any stage, the DSMB concludes it should recommend stopping the trial early, it should then immediately advise the co-ordinating principal investigator, the members of the Trial Steering Committee and the reviewing HREC/IRB.

The DSMB may elect to hold additional meetings in response to notification of serious or non-serious adverse events or if one of more of the committee has/have a trial concern they wish the committee to discuss. The DSMB will record minutes of meetings including date, attendance, meeting duration, outcomes. Any decision to stop the trial requires attendance of all members of the DSMB. If a member is unwilling or unable to serve on the DSMB, the Contract Research Organisation (CRO) will be informed and a replacement will be approached by that organization after consultation with the investigators.

The DMSB may elect to recommend stopping the trial for other reasons. Any such recommendation and its rationale should be shared immediately with the co-ordinating principal investigator, the members of the Trial Steering Committee and the reviewing HREC.

In the unlikely event that the Trial Steering Committee disagrees with such any such DSMB recommendation, the trial may pause recruitment for up to 1 week while the Trial Steering Committee and the HREC/IRB consider the recommendation and its rationale and actively discuss these between them. If after 1 week of such discussion, if the Trial Steering Committee still wishes to continue the trial, the HREC/IRB will determine whether the trial should continue in its current form, continue with modifications to the protocol or cease recruitment. If permission is given to continue the trial in that setting but the Trial Steering Committee subsequently aligns its assessment with the recommendation of the DSMB, the trial will stop early at that point.

- The CRO will alert the data safety monitoring board (DSMB) and principal investigators to serious adverse events immediately on becoming aware of these. Adverse events will be reported to the DSMB monthly.
- The CRO will arrange for the clinical trials pharmacies or the database manager to directly supply the DSMB with the progressive treatment allocation lists as required so

that the DSMB may be unblinded if required for its decisions while maintaining blinding of the CRO and the investigators.

- Suspected unexpected serious adverse reactions (SUSARs) will be reported by the Sponsor to the relevant Regulatory agency/ies, DSMB and reviewing HREC/IRB according to the local requirements.

12.6.2. Trial Steering Committee

A trial steering committee (TSC) will be formed of all blinded principal investigators and the CRO's trial project manager (TPM).

TSC is responsible for the overall project management of the study.

The CRO will organize video conference meetings weekly for the first 2 trial weeks then at least monthly thereafter.

The TPM will record minutes.

The TSC will receive updates at each meeting from the TPM that will include:

- rate of recruitment,
- any problems with application of trial protocol,
- any safety concerns expressed by a member of the MM or DSMB and
- any budgetary or regulatory concerns.

The TSC will recommend responses to any problems brought to its notice by the TPM.

12.6.3. Medical Monitor

One or more specialist doctors will serve as a medical monitor (MM) in each country in which the trial recruits.

The CRO will notify a MM of any adverse event within 24 hours of that organization becoming aware of that adverse event. It may also ask a MM to assist with categorization of an untoward medical occurrence (for example to distinguish an adverse event (AE) from an adverse reaction (AR) or an AR from a serious adverse reaction (SAR), (National Health and Medical Research Council (2016). Guidance: Safety monitoring and reporting in clinical trials involving therapeutic goods. Canberra: National Health and Medical Research Council). Any SAE will be reported by the MM to the sponsor within 24 hours of a MM becoming aware of that event. The CRO will be responsible for preparing a draft of the report for the MM to review and conveying such a report to the sponsor.

Medical monitors within any one country may meet by computer video conference. Meetings will be organized by the CRO to occur monthly. The CRO will arrange for the clinical trials pharmacy or the database master to directly supply the MM with the progressive treatment allocation list as required so that the MM may be unblinded on request for decisions while maintaining blinding of the CRO and the investigators.

In the unlikely event of a secondary or incidental finding, the CRO will seek advice from the MM who may refer to the TSC or DSMB as necessary for advice as the best way to proceed in the clinical interests of the participant.

The MM will review cumulative AE and AR (including serious AE or AR) and express any concerns to the DSMB. (Note the DSMB will already be independently notified as above of SUSARs).

The MM will report to the sponsor. If MM meet by committee, they will record minutes of MM meetings including date, attendance, meeting duration, outcomes. If a MM is unwilling or unable to continue to serve as a MM, the CRO will be informed and a replacement will be approached by that organization after consultation with the investigators.

In this trial,

i) The responsibilities of the co-ordinating principal investigator to capture all AEs will fall to the CRO and that investigator's responsibility of assessment of the AEs will fall to the MM and DSMB as detailed above.
ii) The responsibility to report Serious adverse events (SAE) to the MM will fall to the CRO as above.
iii) The responsibility of the co-ordinating principal investigator to report all safety critical events and any additional information related to reported deaths will fall to the MM. The CRO will collate and organize the data and prepare such reports for the MM who will review and sign them.
iv) The responsibility of the co-ordinating principal investigator to report to the institution within 72 hours of becoming aware of all significant safety issues or SUSARs will fall to the MM for the former and DSMB for the latter and the CRO will collate and organize the data and prepare such reports for the relevant MM and/or committee, who or a member of which, will review and sign them.

12.7. Dissemination of Clinical Study Data

12.7.1. Data Quality Assurance

- All participant data relating to the study will be recorded on an electronic CRF. For this study, the eCRF will be REDCap.
- The CRO collecting data, trial personnel working under instruction of the CRO, the trial investigator and/or his/her personnel must maintain accurate documentation (source data) that supports the information entered in the CRF.
- Study-related monitoring will be performed for this study by country CRO. The CRO will perform logic checks of the data entered REDCap.
- The Trial Project Manager or his/her delegates will perform source data verification to confirm that data entered into the CRF by authorized trial personnel are accurate, complete, and verifiable from source documents; that the safety and rights of participants are being protected; and that the study is being conducted in accordance with the currently approved protocol and any other study agreements, ICH GCP, and all applicable regulatory requirements.

- Records and documents pertaining to the conduct of this study must be retained by the investigators and/or sponsor for 15 years after study completion unless local regulations or institutional policies require a longer retention period. No records may be destroyed during the retention period without the written approval of the sponsor and investigators. No records may be transferred to another location or party without written approval of the sponsor and investigators.

12.7.2. Source Documents

- Source documents provide evidence for the existence of the participant and substantiate the integrity of the data collected.
- Data reported on the CRF or entered in the eCRF that are transcribed from source documents must be consistent with the source documents or the discrepancies must be explained.

12.7.3. Pharmaceutical company supplying investigational product access to data for preparation of regulatory applications

Edenbridge Pharmaceuticals may have confidential access to de-identified (anonymised) patient level data, subject to agreement with the investigators, for the purpose of preparing an application to a regulatory authority. However, this access will not occur prior to the investigators analysing the data. Access to primary outcome data will not occur prior to the investigators analysing the data relevant to the primary outcome. Access to secondary outcome data will not occur prior to the investigators analysing the data relevant to the secondary outcomes. Access to follow up data including up to 6 months will not occur prior to the investigators analysing those data.

12.8. Study Closure

The trial is considered closed when all required documents and study supplies have been collected and closure procedures have been performed.

Reasons for the early closure of the study by the sponsor or investigators may include but are not limited to:

- Failure of the investigators to comply with the protocol, the requirements of the HREC/IRB or local health authorities, the sponsor's procedures, or GCP guidelines
- Inadequate recruitment of participants
- Discontinuation of further study intervention development

12.9. Trial Registration

The sponsor will register the trial on a clinical trials registration site prior to enrolment of first participant. However, given the pressing public health need to know the trial findings, at the discretion

of the TSC, delay with registration (for example the registration site website being under repair) will not be allowed to delay trial commencement.

12.9. Publication Policy

The results of this study will be published or presented at scientific meetings. There should be no restriction placed on the principal investigators' capacity to submit the trial results for publication especially given the potential public health relevance of the trial in the setting of a pandemic.

Authorship will be determined by mutual agreement and in line with International Committee of Medical Journal Editors authorship requirements.

13. List of Abbreviations

AE(s)	adverse event(s)
C_{max}	maximum (peak) concentration of drug
CRF	Case Report Form
CRO	Contract Research Organisation
CYP	cytochrome P450
DRE	Drug Related Event
DSMB	Data Safety Monitoring Board
eCRF	Electronic Case Report Form
FDA	Food and Drug Administration
GP	General Practitioner
HREC	Human Research Ethics Committee
IC_{50}	Half the maximum inhibitory concentration
IEC	Independent Ethics Committee
IRB	Institutional Review Board
MM	Medical Monitor
NTA	Neuroscience Trials Australia (Australian CRO)
PCR	Polymerase Chain Reaction
PICF	Participant Information and Consent Form
RAT	Rapid Antigen Test
SAE	Serious Adverse Event
SARS-CoV-2	Severe Acute Respiratory Syndrome Coronavirus 2
SUSAR	Suspected, Unexpected Serious Adverse Reaction
TGA	Therapeutic Goods Administration
TPM	Trial Project Manager
TSC	Trial Steering Committee

14. References

Arshad, U.; Pertinez, H.; Box, H.; Tatham, L.; Rajoli, R.K.R.et al Prioritisation of Anti- SARS-Cov-2 Drug Repurposing Opportunities Based on Plasma and Target Site Concentrations Derived From Their Established Human Pharmacokinetics. Clin Pharmacol Ther. 2020, doi: 10.1002/cpt.1909.

Barkwell R, Shields S 1997a. Deaths associated with ivermectin treatment of scabies. Lancet. 349(9059):1144-1145.

Barkwell R, Shields S 1997b. Deaths associated with ivermectin for scabies. Lancet. 350(9072):216.

Baudou E, Lespine A, Durrieu G, et al. Serious Ivermectin Toxicity and Human *ABCB1* Nonsense Mutations. N Engl J Med. 2020;383(8):787-789

Blakley BR, Rousseaux CG. Effect of ivermectin on the immune response in mice. Am J Vet Res. 1991;52(4):593-595.

Biber A, Mandelboin M, Harmelin G, Lev D, Ram L, Shaham A, Nemet I, Kliker L, Erster O, Schwartz E 2021 Favourable outcome on viral load and culture viability using Ivermectin in early treatment of non-hospitalized patients with mild COVID-19-A double-blind randomized placebo-controlled trial. MedRxiv https://doi.org/10.1101/2021.05.31.21258081

Bredal WP 1997. Deaths associated with ivermectin for scabies. Lancet. 350(9072):216

Bryant A, Lawrie TA, Dowswell T, Fordham EJ, Mitchell S, Hill SR, Tham TC 2021 Ivermectin for Prevention and Treatment of COVID-19 Infection: A Systematic Review, Meta-analysis, and Trial Sequential Analysis to Inform Clinical Guidelines. Am J Ther Jun 21;28(4):e434-e460.

Caly L, Druce JD, Catton MG, Jans DA, Wagstaff KM 2020 The FDA-approved drug ivermectin inhibits the replication of SARS-CoV-2 in vitro Antiviral Res 2020 Jun;178: Epub 2020 Apr 3.

Cepelowicz Rajter JC, Sherman MS, Fatteh N, Vogel F, Jamie Sacks J, Rajter J-J 2020a ICON (Ivermectin in COvid Nineteen) study: Use of Ivermectin is Associated with Lower Mortality in Hospitalized Patients with COVID19 medRxiv (Preprint) https://doi.org/10.1101/2020.06.06.20124461

Cepelowicz Rajter JC, Sherman MS, Fatteh N, Vogel F, Jamie Sacks J, Rajter J-J 2020b ICON (Ivermectin in COvid Nineteen) Study: Use of Ivermectin is Associated with Lower Mortality in Hospitalized Patients with COVID19 Chest Oct 13:S0012-3692(20)34898-4.

Chaccour C, Hammann F, Rabinovich NR 2017. Ivermectin to reduce malaria transmission I. Pharmacokinetic and pharmacodynamic considerations regarding efficacy and safety. Malar J

16(1):161.

Chaccour C, Hammann F, Ramon-Garcıa S and Rabinovich NG 2020a Ivermectin and COVID-19: Keeping Rigor in Times of Urgency (Editorial) Am. J. Trop. Med. Hyg., 102(6), pp. 1156–1157.

Chaccour C, Ruiz-Castillo P, Richardson MA, Moncunill G, Casellas A, Carmona-Torre F, Giráldez M, Mota JS, Yuste JR, Azanza JR, Fernández M, Reina G, Dobaño C, Brew J, Sadaba B, Hammann F, Rabinovich R. The SARS-CoV-2 Ivermectin Navarra-ISGlobal Trial (SAINT) to Evaluate the Potential of Ivermectin to Reduce COVID-19 Transmission in low risk, non-severe COVID-19 patients in the first 48 hours after symptoms onset: A structured summary of a study protocol for a randomized control pilot trial. Trials. 2020b Jun 8;21(1):498.

Chaccour C, Casellas A, Blanco-Di Matteo A, Pineda I, Fernandez-Montero A, Ruiz-Castillo P, Richardson MA, Rodríguez-Mateos M, Jordán-Iborra C, Brew J, Carmona-Torre F, Giráldez M, Laso E, Gabaldón-Figueira JC, Dobaño C, Moncunill G, Yuste JR, Del Pozo JL, Rabinovich NR, Schöning V, Hammann F, Reina G, Sadaba B, Fernández-Alonso M. The effect of early treatment with ivermectin on viral load, symptoms and humoral response in patients with non-severe COVID-19: A pilot, double-blind, placebo-controlled, randomized clinical trial. EClinicalMedicine. 2021 Feb;32:100720. doi: 10.1016/j.eclinm.2020.100720.

Chandler RE 2018 Serious Neurological Adverse Events after Ivermectin-Do They Occur beyond the Indication of Onchocerciasis? Am J Trop Med Hyg. Feb;98(2):382-388.

Common Terminology Criteria for Adverse Events (CTCAE) v5.0 2017, US Department of Health and Human Services accessed from https://ctep.cancer.gov/protocoldevelopment/eleCROnic_applications/docs/CTCAE_v5_Quick_Reference_5x7.pdf

Corbo-Rodgers E, Staub ES, Zou T, Smith A, Kambayashi T, Maltzman JS 2012 Oral ivermectin as an unexpected initiator of CreT2-mediated deletion in T cells. Nat Immunol 13(3):197-8.

Coyne PE, Addiss DG 1997. Deaths associated with ivermectin for scabies. Lancet. 350(9072):215-216.

Crump A, Omura S 2011 Ivermectin, ‘Wonder drug’ from Japan: the human use perspective (review) Proc. Jpn. Acad., Ser. B 87:13-28.

Currie BJ, McCarthy JS. Permethrin and ivermectin for scabies. N Engl J Med. 2010 Feb 25;362(8):717-25

Edwards G 2003 Ivermectin: does P-glycoprotein play a role in neurotoxicity? Filaria Journal

2(Suppl 1) S8.

Guzzo CA, Furtek, CI, Porras AG, Chen C, Tipping R et al 2002 Tolerability, and Pharmacokinetics of Escalating High Doses of Ivermectin in Healthy Adult Subjects Journal of Clinical Pharmacology, 42:1122-1133.

Khan MSI, Khan MSI, Debnath CR, Nath PN, Mahtab MA, Nabeka H, Matsuda S, Akbar SMF 2020a. Ivermectin Treatment May Improve the Prognosis of Patients With COVID-19. Arch Bronconeumol 56(12):828-830. English, Spanish. doi: 10.1016/j.arbres.2020.08.007.

Khan MSI, Khan MSI, Debnath CR, Nath PN, Mahtab MA, Nabeka H, Matsuda S, Akbar SMF 2020b. Reply to "Ivermectin Treatment May Improve the Prognosis of Patients With COVID-19". Arch Bronconeumol S0300-2896(20)30554-8. doi: 10.1016/j.arbres.2020.12.013.

Krolewiecki A, Lifschitz A, Moragas M, Travacio M, Valentini R, Alonso DF, Solari R, Tinelli MA, Cimino RO, Álvarez L, Fleitas PE, Ceballos L, Golemba M, Fernández F, Fernández de Oliveira D, Astudillo G, Baeck I, Farina J, Cardama GA, Mangano A, Spitzer E, Gold S, Lanusse C 2021 Antiviral effect of high-dose ivermectin in adults with COVID-19: A proof-of-concept randomized trial. EClinicalMedicine.;37:100959

Lifschitz A, Virkel G, Sallovitz J, Sutra JF, Galtier P, Alvinerie M, Lanusse C 2000. Comparative distribution of ivermectin and doramectin to parasite location tissues in cattle. Vet Parasitol ;87(4):327-38

Mitjà O, Corbacho-Monné M, Ubals M, Alemany A, Suñer C et al 2021 A Cluster-Randomized Trial of Hydroxychloroquine for Prevention of Covid-19. N Engl J Med Feb 4;384(5):417-427.

National Health and Medical Research Council (2016). Guidance: Safety monitoring and reporting in clinical trials involving therapeutic goods. Canberra: National Health and Medical Research Council.

National Health and Medical Research Council (2018), Data Safety Monitoring Boards (DSMBs)

National Health and Medical Research Council (2018), Risk-based Management and Monitoring of Clinical Trials Involving Therapeutic Goods.

National Statement on Ethical Conduct in Human Research 2007 (Updated 2018). The National Health and Medical Research Council, the Australian Research Council and Universities Australia. Commonwealth of Australia, Canberra.

Popp M, Stegemann M, Metzendorf MI, Gould S, Kranke P, Meybohm P, Skoetz N, Weibel S 2021. Ivermectin for preventing and treating COVID-19. Cochrane Database Syst Rev. Jul

28;7(7):CD015017.

Reintjes R, Hoek C. 1997 Deaths associated with ivermectin for scabies. Lancet. 350(9072):215-216.

Sajid MS, Iqbal Z, Muhammad G, et al 2007. Effect of ivermectin on the cellular and humoral immune responses of rabbits. Life Sci. 80(21):1966-1970.

Schmith, V.D.; Zhou, J.J.; Lohmer, L.R. The Approved Dose of Ivermectin Alone is not the Ideal Dose for the Treatment of COVID-19. Clin Pharmacol Ther. 2020, doi: 10.1002/cpt.1889.

Sistonen J, Madadi P, Ross CJ, Yazdanpanah M, Lee JW, Landsmeer ML, Nauta M, Carleton BC, Koren G, Hayden MR 2012. Prediction of codeine toxicity in infants and their mothers using a novel combination of maternal genetic markers. Clin Pharmacol Ther. Apr;91(4):692-9.

Smit MR, Ochomo EO, Aljayyoussi G, Kwambai TK, Abong'o BO, Chen T, Bousema T, Slater HC, Waterhouse D, Bayoh NM, Gimnig JE, Samuels AM, Desai MR, Phillips-Howard PA, Kariuki SK, Wang D, Ward SA, Ter Kuile FO 2018. Safety and mosquitocidal efficacy of high-dose ivermectin when co-administered with dihydroartemisinin-piperaquine in Kenyan adults with uncomplicated malaria (IVERMAL): a randomized, double-blind, placebo-controlled trial. Lancet Infect Dis. 18(6):615-626.

Smit MR, Ochomo EO, Waterhouse D, Kwambai TK, Abong'o BO, Bousema T, Bayoh NM, Gimnig JE, Samuels AM, Desai MR, Phillips-Howard PA, Kariuki SK, Wang D, Ter Kuile FO, Ward SA, Aljayyoussi G 2019 Pharmacokinetics-Pharmacodynamics of High-Dose Ivermectin with Dihydroartemisinin-Piperaquine on Mosquitocidal Activity and QT-Prolongation (IVERMAL). Clin Pharmacol Ther 105(2):388-401

Stankiewicz M, Cabaj W, Jonas WE, Moore LG, Millar K, Ng Chie W 1995. Influence of ivermectin on cellular and humoral immune responses of lambs. Vet Immunol Immunopathol 44(3-4):347-358.

Stromectol Product Information

Ventre E, Rozières A, Lenief V, et al 2017 Topical ivermectin improves allergic skin inflammation. Allergy.72(8):1212-1221.

Wagstaff KM, Sivakumaran H, Heaton SM, Harrich D, Jans DA 2012. Ivermectin is a specific inhibitor of importin alpha/beta mediated nuclear import able to inhibit replication of HIV-1 and dengue virus. Biochem. J. 443 (3), 851–856.

Appendix 1: Stromectol (Ivermectin) Product Information

AUSTRALIAN PRODUCT INFORMATION STROMECTOL BLISTER PACK (ivermectin) Tablets

1 NAME OF THE MEDICINE

Ivermectin

2 QUALITATIVE AND QUANTITATIVE COMPOSITION

Ivermectin 3 mg tablets.
Ivermectin is derived from the avermectins, a class of highly active broad-spectrum antiparasitic agents isolated from fermentation broths of *Streptomyces avermitilis*.
For the full list of excipients, see **Section 6.1 List of Excipients**.

3 PHARMACEUTICAL FORM

STROMECTOL (ivermectin) is available as white, round, flat tablets with a bevelled edge, engraved with MSD on one side and 32 on the other side.

4 CLINICAL PARTICULARS

4.1 THERAPEUTIC INDICATIONS

STROMECTOL (ivermectin) is indicated for the treatment of:

a) onchocerciasis and intestinal strongyloidiasis (anguillulosis).
b) crusted scabies in conjunction with topical therapy.
c) human sarcoptic scabies when prior topical treatment has failed or is contraindicated.

Treatment is only justified when the diagnosis of scabies has been established clinically and/or by parasitological examination. Without formal diagnosis, treatment is not justified in case of pruritus alone.

4.2 DOSE AND METHOD OF ADMINISTRATION

STROMECTOL is available as white tablets each containing 3 mg ivermectin. Treatment is administered as a single oral dose given with water.
The dose is determined by the patient's weight as shown below.

Strongyloidiasis

The dosage aims to provide approximately 200 µg ivermectin/kg body weight.

Table 1
Dosage in Strongyloidiasis

Body Weight (Kg):	Dose (Number of 3 mg tablets):
15-24	One
25-35	Two

36-50	Three
51-65	Four
66-79	Five
≥ 80	Approx 200 □g/kg

In general, additional doses are not necessary, however, a follow-up examination of stool to verify efficacy should be performed.

Onchocerciasis

The dosage aims to provide approximately 150 µg ivermectin/kg body weight.

Table 2 Dosage in Onchocerciasis:

Body Weight (Kg):	Dose (Number of 3 mg tablets):
15-25	One
26-44	Two
45-64	Three
65-84	Four
Alternatively, the dose of ivermectin for use in mass chemotherapy campaigns may be determined by the patients height, as follows:	
Height (cm):	Dose (Number of 3 mg tablets):
90-119	One
120-140	Two
141-158	Three
> 158	Four
[Reference: Alexander, N.D.E., ivermectin dose assessment without weighing scales. Bulletin of the World Health Organisation, 71 (3/4): 361-366 (1993)]	

The suggested dose interval for most patients is 12 months. At some sites it may be preferable to use a 6 months interval depending on such considerations as density of skin microfilariae and/or prevalence.

Sarcoptes scabiei (scabies)

The dosage aims to provide approximately 200 µg ivermectin/kg body weight per dose.

Table 3

Dosage in *Sarcoptes scabiei* (scabies)

Body Weight (Kg):	Dose (Number of 3 mg tablets):
15-24	One
25-35	Two
36-50	Three
51-65	Four
66-79	Five
> 80	Approx 200 □g/kg

Classic/typical scabies:

2 doses (1 dose on day 1 and another dose between day 8 and day 15). Ivermectin can be used alone or in combination with a topical scabicide.

Crusted scabies (ivermectin in combination with a topical scabicide administered as):

- Mild cases: 2 doses (1 dose on day 1 and another dose between day 8 and day 15).
- Moderate to severe cases: More than 3 doses may be required for effective treatment.

Patients with crusted scabies should use keratolytics on days they are not treated with topical scabicides to assist with the reduction of scaling that harbours the mite. Consultation with a dermatologist or infectious diseases physician is recommended.
The life cycle of *S. scabiei* begins with the pregnant female laying two to three eggs a day in burrows several millimetres to several centimetres in length in the stratum corneum (outermost layer) of the skin. The eggs hatch in two weeks. The larvae form intra-epidermal lesions whilst they mature into the adult form. This maturation takes 2-3 weeks.

4.3 CONTRAINDICATIONS

Hypersensitivity to any component of the product.

4.4 SPECIAL WARNINGS AND PRECAUTIONS FOR USE

If any hypersensitivity reaction to this product occurs, no further dose should be given.
Neurological toxicity, including depressed level of consciousness and coma, has also been reported in patients with the use of ivermectin in the absence of *Loa loa* infection.

For treatment of onchocerciasis

Ivermectin should be used only when infections with *O. volvulus* have been diagnosed or are suspected. No data are available to support its use prophylactically.
After treatment with microfilaricidal drugs, patients with hyperreactive onchodermatitis (sowda) may be more likely than others to experience severe adverse reactions, especially oedema and aggravation of onchodermatitis.
Rarely, patients with onchocerciasis who are also heavily infected with *Loa Loa* may develop a serious or even fatal encephalopathy either spontaneously or following treatment with an effective microfilaricide. In these patients, the following adverse experiences have also been reported: pain (including neck and back pain), red eye, conjunctival haemorrhage, dyspnoea, urinary and/or faecal incontinence, difficulty in standing/walking, mental status changes, confusion, lethargy, stupor, seizures or coma. This syndrome has been seen very rarely following the use of ivermectin.

Use in hepatic impairment

Ivermectin has not been studied in patients with impaired hepatic function. As ivermectin is extensively metabolised by the liver, caution should be exercised if ivermectin is administered to patients with impaired hepatic function.

Use in renal impairment

Ivermectin has not been studied in patients with impaired renal function.

Use in the elderly

Clinical studies of STROMECTOL did not include sufficient numbers of elderly subjects aged 65 years and over to determine whether they respond differently from younger subjects. In general, treatment of elderly patients should be cautious, reflecting the greater frequency of decreased hepatic, renal or cardiac function, and of concomitant disease or other drug therapy.

Paediatric use Onchocerciasis:

Ivermectin should not be used in children under five years of age as safety in this age group has not been established.
The safety profile of ivermectin in children 5 to 12 years of age is similar to that observed in adults (see

Section 4.8 Adverse Effects (Undesirable Effects), Onchocerciasis).
Strongyloidiasis:
Efficacy has not been established in children under twelve years of age.
Sarcoptes scabiei (scabies):
Ivermectin should not be used in children under 15 kg and under 5 years of age as safety in these groups has not been established.

Effects on laboratory tests

No data are available.

4.5 INTERACTIONS WITH OTHER MEDICINES AND OTHER FORMS OF INTERACTIONS

Interactions between ivermectin and other drugs have not been studied in clinical trials.
Very rare post-marketing reports of increased INR (International Normalised Ratio) have been reported when ivermectin was co-administered with warfarin.

4.6 FERTILITY, PREGNANCY AND LACTATION

Effects on fertility

No data are available.

Use in pregnancy (Category B3)
Ivermectin should not be used in pregnancy as safety in pregnancy has not been established. Ivermectin caused cleft palates in mice and rats at oral doses of 0.4 and 10 mg/kg/day respectively, and cleft palates and clubbed feet in rabbits dosed at 3 mg/kg/day.
Use in lactation
Ivermectin is excreted in breast milk and safety in newborn infants has not been established. In rats, reduced survival occurred in control pups and pups exposed *in utero* that were crossfostered to treated dams, but not in control pups or pups exposed *in utero* cross-fostered to control dams.
The drug should be given to nursing mothers only if the benefit to the mother outweighs the potential risk to the breast-fed infant, and the treatment of mothers who intend to breast-feed their infants should be delayed until at least one week after the birth of the child.

4.7 EFFECTS ON ABILITY TO DRIVE AND USE MACHINES

The effects of this medicine on a person's ability to drive and use machines were not assessed as part of its registration.

4.8 ADVERSE EFFECTS (UNDESIRABLE EFFECTS)

Clinical Trial and Published Data
Strongyloidiasis:

Ivermectin has been demonstrated to be generally well tolerated in the treatment of strongyloidiasis. In three clinical studies involving a total of 109 patients given either one or two doses of 170200 µg/kg of ivermectin, the following adverse reactions were reported as possibly, probably, or definitely related to ivermectin:

Body as a whole: asthenia/fatigue (0.9%), abdominal pain (0.9%)
Gastrointestinal: anorexia (0.9%), constipation (0.9%), diarrhoea (1.8%), nausea (1.8%), vomiting (0.9%)
Nervous System/Psychiatric: dizziness (2.8%), somnolence (0.9%), vertigo (0.9%), tremor (0.9%)
Skin: pruritus (2.8%), rash (0.9%), and urticaria (0.9%)
Ivermectin was better tolerated than thiabendazole in comparative studies involving 37 patients treated with thiabendazole.
In a WHO sponsored study, children and adults (9-22 years) with strongyloidiasis were treated with ivermectin 200 µg/kg as a single dose or albendazole 200 mg twice daily for 3 days. The safety results are summarised in the following table:

Table 4

WHO Study - Number of Patients Developing Adverse Effects Within 3 Days After Treatment, Regardless of Drug Relationship

Body System/ Symptoms	Ivermectin (n=163)	Albendazole (n=170)
Body As A Whole		
Abdominal Distension	7*	1
Chest Pain/Tightness	7*	0
Fever	10	7
Digestive System		
Loose Stools	16	17
Nausea	5	6
Watery Diarrhoea	2	3
Nervous System		
Headache	15	18
Dizziness, vertigo	5	10
Skin		
Diffuse Itching	3	6
Respiratory System		
Cough	11	8

* = $p < 0.05$

The Mazzotti-type and ophthalmologic reactions associated with the treatment of onchocerciasis or the disease itself would not be expected to occur in strongyloidiasis patients treated with ivermectin (see **Section 4.8 Adverse Effects (Undesirable Effects), Onchocerciasis**).

Laboratory Test Findings:

In clinical trials involving 109 patients given either one or two doses of 170-200 µg/kg ivermectin, the following laboratory abnormalities were seen irrespective of drug relationship: elevation in ALT and/or AST (2%), decrease in leukocyte count (3%). Leukopenia and anaemia were seen in one patient.

Onchocerciasis:

Ivermectin has been demonstrated to be generally well tolerated in the treatment of onchocerciasis. Historical data have shown that microfilaricidal drugs, such as diethylcarbamazine citrate (DEC-C), might cause cutaneous and/or systemic reactions of varying severity (the Mazzottitype reaction) and ophthalmological reactions in patients with onchocerciasis. These reactions are probably due to allergic and inflammatory responses to the death of microfilariae. Patients treated with ivermectin for onchocerciasis may experience these reactions in addition to clinical adverse reactions possibly, probably, or definitely related to the drug itself.

In clinical trials involving 963 adult patients treated with 100 to 200 µg/kg ivermectin and 315 patients on placebo, worsening of the following Mazzotti-type reactions during the first 4 days post-treatment were reported (ivermectin, placebo, respectively): arthralgia/synovitis (9.3%, 4.4%), axillary lymph node enlargement (11.0%, 2.9%), axillary lymph node tenderness (4.4%, 1.0%), cervical lymph node enlargement (5.3%, 4.1%), cervical lymph node tenderness (1.2%, 0.6%), inguinal lymph node enlargement (12.6%, 6.7%), inguinal lymph node tenderness (13.9%, 5.7%), other lymph node enlargement (3.0%, 1.6%), other lymph node tenderness (1.9%, 0.6%), pruritus (27.5%, 17.2%), skin involvement including oedema, papular and pustular or frank urticarial rash (22.7%, 9.2%), and fever (22.6%, 4.8%).

In clinical trials, ophthalmological conditions were examined in 963 adult patients before treatment, at day 3 and months 3 and 6 after treatment with 100 to 200 µg/kg ivermectin. Changes observed were primarily deterioration from baseline 3 days post-treatment. Most changes either returned to baseline condition or improved over baseline severity at the month 3 and 6 visits. The percentages of patients with worsening of the following conditions at day 3, month 3 and 6, respectively, were: limbitis: 5.5%, 4.8%, and 3.5% and punctate opacity: 1.8%, 1.8%, and 1.4%. The corresponding percentages for patients treated with placebo were: limbitis: 6.2%, 9.9% and 9.4% and punctate opacity: 2.0%, 6.4% and 7.2%.

In clinical trials involving 963 adult patients who received 100 to 200 µg/kg ivermectin and 315 patients on placebo, the following clinical adverse reactions were reported as possibly, probably, or definitely related to the drug in ≥ 1% of the patients (ivermectin and placebo, respectively): facial oedema (1.2%, 0%), peripheral oedema (3.2%, 0.6%), orthostatic hypotension (1.1%, 0%), and tachycardia (3.5%, 0.6%). Drug-related headache and myalgia occurred in < 1% of patients given ivermectin (0.2%, and 0.4%, respectively). However, these were the most common adverse experiences reported overall during these trials regardless of causality (22.3%, and 19.7%, respectively).

A similar safety profile was observed in an open study in paediatric patients ages 5 to 12.

The following ophthalmological side effects do occur due to the disease itself but have also been reported after treatment with ivermectin: abnormal sensation in the eyes, eyelid oedema, anterior uveitis, conjunctivitis, limbitis, keratitis, and chorioretinitis or choroiditis. These have rarely been severe or associated with loss of vision and have generally resolved without corticosteroid treatment.

Laboratory Test Findings:

In controlled clinical trials, the following laboratory adverse experiences were reported as possibly, probably, or definitely related to the drug in ≥ 1% of the patients (ivermectin and placebo, respectively): oeosinophilia (3%, 0%) and haemoglobin increase (1%, 0%).

Sarcoptes scabiei (scabies):

Adverse effects reported in the literature review were similar to those reported in clinical trials for the other indications. The most common adverse drug reactions reported with ivermectin in the review involved the transient exacerbation of pruritus that can sometimes occur as a result of sensitisation of the human host to mite antigens, with a consequent immunologic reaction (1.4%). Sensitisation also frequently results in delayed resolution of symptoms. Patients should be warned that itching may persist for one to two weeks after treatment, even if the mite is successfully eradicated. Other

frequently reported reactions consisted of headache (< 1.0%), arthralgia (< 1.0%) and anorexia (< 1.0%). Additionally, lethargy (< 1.0%), listlessness (< 1.0%), abdominal discomfort (< 1.0%), rash (< 1.0%), and dizziness (< 1.0%) were reported. Up to 1.86% of patients noticed that they had passed Ascaris worms during the week after treatment.

Post-Marketing Experience

Onchocerciasis: Conjunctival haemorrhage

All Indications:

Very rarely, hypotension (mainly orthostatic hypotension), worsening of bronchial asthma, toxic epidermal necrolysis, Stevens-Johnson syndrome, seizures, elevation of liver enzymes and elevation of bilirubin.

Neurological toxicity including depressed level of consciousness and coma (see **Section 4.4 SPECIAL WARNINGS AND PRECAUTIONS FOR USE**).

Reporting suspected adverse effects

Reporting suspected adverse reactions after registration of the medicinal product is important. It allows continued monitoring of the benefit-risk balance of the medicinal product. Healthcare professionals are asked to report any suspected adverse reactions at www.tga.gov.au/reporting-problems.

4.9 OVERDOSE

There are reports of accidental overdosing of ivermectin, but no fatalities have been attributable to ivermectin overdosing.

It is important to adhere to recommended dosages. Cases of depressed level of consciousness and coma have been reported with overdosage of ivermectin.

In significant accidental intoxication with unknown quantities of a veterinary formulation, symptoms have resembled those seen in animal toxicology studies, which were chiefly rash, contact dermatitis, oedema, headache, dizziness, asthenia, nausea, vomiting, diarrhoea, mydriasis, somnolence, depressed motor activity, tremors and ataxia. Other adverse effects that have been reported include: seizure, dyspnoea, abdominal pain, paraesthesia and urticaria.

In case of accidental poisoning, supportive therapy, if indicated, should include parenteral fluids and electrolytes, respiratory support (oxygen and mechanical ventilation if necessary) and pressor agents if clinically significant hypotension is present. Induction of emesis and/or gastric lavage as soon as possible, followed by purgatives and other routine antipoison measures may be indicated if needed to prevent absorption of ingested material. Although data are not available for man, it would appear advisable to avoid GABA-agonistic drugs in the treatment of accidental ivermectin intoxication.

In a study in which healthy volunteers were orally administered up to 2000 µg/kg ivermectin in a fasted state or up to 600 µg/kg ivermectin following a high-fat (48.6 g of fat) meal, there were no indications of central nervous system toxicity observed at any dose irrespective of food intake.

For information on the management of overdose, contact the Poison Information Centre on 131126 (Australia).

5 PHARMACOLOGICAL PROPERTIES

5.1 PHARMACODYNAMIC PROPERTIES

Mechanism of action

Ivermectin inhibits signal transmission from the ventral cord interneurons to the excitatory motor neurones in nematodes by stimulating release of the inhibitory neurotransmitter, gamma-aminobutyric acid (GABA) from pre-synaptic nerve terminals. In arthropods, a similar mechanism inhibits signal transmission at the neuromuscular junction. Ivermectin does not readily penetrate the CNS of

mammals, and thus does not interfere with mammalian GABAdependent neurotransmission.

Clinical trials

Onchocerciasis in Adults:

A total of 758 adult patients with onchocerciasis received a single oral dose of 150-220 µg/kg ivermectin in three studies. In these studies, a significant reduction in microfilarial counts was reported in evaluable patients who received ivermectin, compared to those who received diethylcarbamazine or placebo. At 2-4 days after ivermectin dosing, microfilariae (mf) counts less than 5 per mg skin were reported in 29% to 96% of evaluable subjects, at 3 months after dosing < 5 mf/mg were reported in 88% to 96% of evaluable subjects, and at 12 months after dosing < 5 mf/mg were reported in 48% to 62% of evaluable patients. In patients with ocular involvement there was a significant reduction in intraocular microfilariae maintained for up to 12 months.

Onchocerciasis in Children:

In an open study, ivermectin reduced skin microfilaria in children with onchocerciasis for up to 12 months. One hundred and three (103) children aged between 5 to 12 years were treated with the targeted dose of 150 µm/kg ivermectin as a single oral dose. Resulting geometric mean microfilariae (mf) counts fell from a pre-treatment value of 36.4 mf/mg skin to 5.8%, 0.7%, 2.2% and 5.4% of the pre-treatment value on day 3, month 3, month 6 and month 12 respectively.

Intestinal Strongyloidiasis:

Two controlled clinical studies using albendazole as the comparative agent were carried out in countries where albendazole is approved for the treatment of strongyloidiasis of the gastrointestinal tract, and two controlled studies were carried out using thiabendazole as the comparative agent. Efficacy, as measured by cure rate, was defined as the absence of larvae in follow-up stool examinations post-therapy. Based on this criterion, efficacy was significantly greater for ivermectin (a single dose of 170 to 200 µg/kg) than for albendazole (200 mg b.i.d. for 3 days). Ivermectin administered as a single dose of 200 µg/kg for 1 day was as efficacious as thiabendazole administered at 25 mg/kg b.i.d. for 3 days.

Table 5

Summary of Cure Rates for Ivermectin versus Comparative Agents in the Treatment of Strongyloidiasis

	Cure Rate* (%)	
	Ivermectin**	Comparative Agent
Albendazole*** Comparative		
International Study	22/28 (79)	10/23 (43)
WHO Study	126/152 (83)	67/149 (45)
Thiabendazole† Comparative		
International Study	10/15 (67)	13/15 (87)
US Study	14/14 (100)	16/17 (94)

* Number and % of evaluable patients

** 170-200 □g/kg

*** 200 mg b.i.d. for 3 days

† 25 mg/kg b.i.d. for 3 days

Sarcoptes Scabiei:

Classic variety/Typical Scabies:

The clinical data evaluated for this indication were from a literature-based submission which uncovered 8 randomized controlled trials of ivermectin in typical scabies (see **Section 11**

References), as well as a number of reviews, including a 2010 Cochrane Review (Strong and Johnstone, 2010).

Placebo-controlled studies:

Macotela-Ruiz *et al.* (1993) conducted a trial comparing a single dose of ivermectin 200 μg/kg body weight with placebo in the treatment of scabies in 55 patients. The authors found that 26 (79.3%) patients were cured, defined as absence of itching and no dermatologically active lesions, with ivermectin on their first follow-up visit vs. 4 (16%) in the placebo group ($p<0.001$). The overall cure rates were 37 (74%) and 4 (16%) for ivermectin and placebo, respectively ($p<0.001$).

Active-comparator trials:

Bachewar *et al.* (2009) conducted a non-blinded, prospective, randomized trial comparing benzyl benzoate 25% lotion (applied nightly for 2 nights), permethrin 5% cream (applied once), and a single dose of ivermectin 200 μg/kg body weight in 103 patients with noncrusted scabies. The primary outcome of interest was cure rate, defined as no lesions, measured after 1 and 2 weeks.

After 1 week, the cure rates were 76%, 82.1%, and 55.6% for benzyl benzoate, permethrin, and ivermectin, respectively ($p<0.05$ for permethrin vs. ivermectin). After 2 weeks, the cure rates were 92% for benzyl benzoate, 96.4% for permethrin, and 100% for ivermectin.

Chouela *et al.* (1999) compared ivermectin and lindane in a randomized, controlled, doubleblind study in 53 patients with scabies. Patients received either a single oral dose of ivermectin 150-200 μg/kg body weight or topical application of 1% lindane solution. Treatment was repeated if clinical cure had not occurred, with clinical cure defined as absence of both pruritus and clinical lesions or a reduction in severity of signs and symptoms to 'mild'.

At day 15, 14/19 (74%) patients in the ivermectin group showed healing compared with 13/24 (54%) in the lindane group ($p=0.22$). At day 29, 18/19 (95%) patients receiving ivermectin were healed, and 23/24 (96%) receiving lindane were healed ($p>0.99$).Further analysis of the equivalence of therapeutic efficacy showed that ivermectin was statistically no less efficacious than lindane ($p<0.02$).

Ly *et al.* (2009) compared 2 regimens of benzyl benzoate (BB) and ivermectin in a randomized, open-label trial in 181 patients with scabies. BB was administered as either one application of 12.5% benzyl benzoate which was not removed for 24 hours (BB1), or as two such applications, each over 24 hours (BB2). Ivermectin was administered as a single oral dose of 150-200 μg/kg body weight.

The primary outcome measure was complete disappearance of visible lesions and itching at day 14. Patients who had clearly worsened at day 7 were given the same treatment as the week before. If no change was noted at day 7 or if the patient had improved, nothing further was done until day 14. If treatment failure was observed at day 14, the treatment was applied again. If treatment failure was observed again at day 28, patients in the BB1 and ivermectin groups were switched to the BB2 application, and those in the BB2 group were switched to ivermectin.

For the primary endpoint of efficacy at day 14, cure rates were 37/68 (54.4%) with BB1, 33/48 (68.8%) with BB2, and 16/65 (24.6%) with ivermectin, with a significance level of $p<10^{-6}$ overall. The comparison of BB1 and BB2 combined was also superior to ivermectin ($p<10^{-5}$). At day 28, cure rates were 52/68 (76.5%) for BB1, 46/48 (95.8%) for BB2, and 28/65 (43.1%) for ivermectin ($p<10^{-5}$ overall).

Madan *et al.* (2001) compared ivermectin and 1% topical lindane solution in a randomized trial in 200 patients with scabies. Patients received either a single dose of ivermectin 200 μg/kg body weight or 1% lindane topical solution applied overnight for 1 night. Efficacy was evaluated over the first 3 days, and at 2 weeks and 4 weeks. Improvement was measured on a categorical scale of: Complete/100% (no signs or symptoms of scabies); Good/75% (itching absent, lesions except burrows present; Moderate/50% (Itching markedly reduced, burrows +); Slight/25% (Itching slightly reduced, burrows ++); Poor/0% (no improvement); Negative (Further aggravation/exacerbation).

After 48 hours of treatment, no patients in either treatment group reported complete improvement, but

17.5% of patients in the ivermectin group and 7.1% in the lindane group reported good improvement. After 2 weeks, 36.1% of patients in the ivermectin group had complete improvement compared with 22.4% in the lindane group. At 4 weeks, 82.6% of patients in the ivermectin group had complete improvement compared with 44.4% in the lindane group. After 4 weeks, 2.8% of patients in the ivermectin group reported negative improvement compared with 4.9% in the lindane group.

Mushtaq *et al.* (2010) compared ivermectin and permethrin 5% cream in 100 patients with scabies. Patients received either a single dose of ivermectin 200 µg/kg body weight or a single application of topical permethrin 5% cream at night on the whole body for 12 hours. Patients not responding after the 1 week dose were given a second dose. Efficacy was determined by disappearance of itching, clearance of skin lesions, and absence of mites on microscopy of skin lesions.

Two weeks after the first dose, 24 (54.5%) patients in the ivermectin group were cured of disease, and 20 (47.6%) were cured in the permethrin group (p=0.5). Cure rates at 4 weeks were 79.5% and 88.1%, respectively (p=0.157). Itching at 4 weeks was mild in 6.8% of patients in the ivermectin group, and 2.4% in the permethrin group. No patients in either group had positive mite scrapings at week 2.

Nnoruka and Ague (2001) evaluated ivermectin and 25% benzyl benzoate topical application in an uncontrolled prospective study in 58 patients with scabies. Patients received ivermectin as a single oral dose at 200 µg/kg body weight or 25% benzyl benzoate applied from neck to toes for 72 hours. Clinical scores were calculated using skin lesions located in 16 different locations of the body. An area was given a score of 2 if there was at least one active lesion, a score of 1 if the most important lesion was healed, and a score of 0 if there was no lesion present. A classical burrow was scored a 2. A patient's clinical score was the sum off all individual area scores, which could range from 2 to 30 at study entry and from 0 to 30 at subsequent visits. In addition, pruritus was measured on a 10 cm visual analogue scale, where 0 = not present and 10 = extremely severe.

Mean clinical scores at baseline were 16.1 for ivermectin and 16 for benzyl benzoate. At day 7, scores were 5.4 and 10.3, respectively. At day 14 scores were 2.3 and 6.1, respectively, and at day 30 scores were 1.1 and 3.5 respectively. Analysis of variance showed a significant decrease in scores with ivermectin (p=0.004). At day 7, 31% of patients receiving ivermectin had complete disappearance of lesions and pruritus. On day 14, 65.3% of patients receiving ivermectin and 34.5% of patients receiving benzyl benzoate were healed. By day 30, 93.7% of patients receiving ivermectin were healed. By day 30, response to pruritus was rated as excellent in 93.1% of patients receiving ivermectin, and 48.3% in patients receiving benzyl benzoate.

Usha and Nair (2000) conducted a randomized trial comparing ivermectin and permethrin cream in the treatment of scabies in 85 patients and their family contacts. Ivermectin was administered as a single oral dose of 200 µg/kg body weight, and 5% permethrin cream was given as a single overnight topical application. Treatment was considered to be effective if, after 2 weeks, there was improvement in pruritus (measured on a visual analogue scale), clinical improvement in skin lesions with no new lesions, and absence of mites or the products on microscopy. Improvement was considered mild if there was less than 50% reduction in the number of lesions and pruritus; moderate if there was more than 50% reduction; and good if there was complete clearance. Treatment was considered a failure if, after 2 weeks, there was no improvement in pruritus and skin lesions, appearance of new lesions, or presence of mites or their products on microscopy. In the case of treatment failure, another dose of the randomized treatment was repeated. If the repeat treatment failed, patients were crossed over to the other treatment group. The primary endpoint was the complete disappearance of clinical signs and symptoms and no appearance of new lesions at the end of 2 months after the last dose was taken.

After the first week, 50% of patients in the ivermectin group and 84.3% of patients in the permethrin group had symptom improvement. By the second week, 70% in the ivermectin group and 97.8%, in the permethrin group improved. By the fourth week, 95% of patients in the ivermectin and 100% of patients in the permethrin groups improved. Two (5%) patients did not respond to 2 doses of ivermectin; both were cured after crossing over to permethrin after a single application. There was no

recurrence of scabies at the end of 2 months of followup in either group. Complete clearance of lesions (graded as “good” improvement) occurred earlier with permethrin than with ivermectin, with significant differences at weeks 1 (p=0.03), 2 (p=0.003), and 4 (p=0.0005). At 8 weeks, all patients were completely cured (p=0.21).

Crusted variety:

No randomised controlled trials in crusted scabies have been reported in the literature. A systematic review of the literature on crusted scabies identified observational studies, case series and individual case reports in which 260 patients (mean age (range) 41.7 years (2-94 years)) received ivermectin. Of these cases, 182 received ivermectin at a dose of 200 µg/kg of body weight.

Seventy eight percent (78%) of all patients were managed in a clinic setting following confirmation of high mite count. The majority of patients for whom this was reported (70%) received ivermectin after proving refractory to classical topical treatments.

At least 72% of cases were treated with combination ivermectin and topical scabicide. In the large Australian cohort studies, all patients were administered topical keratolytic therapy in keeping with Australian clinical protocols.

Ivermectin was shown to have an overall clinical efficacy across these publications of an 87% cure rate, however, this must be interpreted with caution because of the potential for publication bias (i.e. publication of cases with good outcomes). The majority of patients presenting with mild to severe forms of crusted scabies were adequately managed with one to two oral doses of 200 µg/kg ivermectin. One to two doses may not be adequate in treating very severe cases of crusted scabies.

5.2 PHARMACOKINETIC PROPERTIES

Absorption and distribution

Ivermectin is incompletely absorbed (~50% bioavailable relative to an oral hydroalcoholic solution) following oral doses of ivermectin tablets, with a T_{max} of ~4 hours. With 12 mg single dose tablets administered in healthy male volunteers, the mean peak plasma concentration of the major component was 46.6 (± 21.9) ng/mL (range 16.4-101.1 ng/mL).

Administration of 30 mg (333 to 600 µg/kg) ivermectin following a high-fat meal resulted in an approximate 2.5-fold increase in bioavailability relative to administration of 30 mg ivermectin in the fasted state in healthy volunteers.

Metabolism and excretion

Ivermectin is metabolised in humans, and ivermectin and/or its metabolites are excreted almost exclusively in the faeces over an estimated 12 days with less than 1% of the administered dose being excreted in the urine. The plasma half-life of ivermectin in man is about 12 hours (9.8-14.3 h) and that of the metabolites is about 3 days.

The pharmacokinetics of ivermectin has not been studied in patients with impaired hepatic or renal function.

5.3 PRECLINICAL SAFETY DATA

Genotoxicity

Ivermectin was negative in three *in vitro* assays for genotoxicity (mutagen assays in bacteria and mouse cells, and unscheduled DNA synthesis in human cells). No tests have been done to test the potential of ivermectin for producing clastogenicity.

Carcinogenicity

There have been no carcinogenicity studies with ivermectin. Ninety-four and 105 week carcinogenicity studies on mice and rats respectively were conducted with the closely related compound abamectin and were negative at up to 8 mg/kg/day in mice and up to 2 mg/kg/day in rats.

6 PHARMACEUTICAL PARTICULARS

6.1 LIST OF EXCIPIENTS

Each tablet of STROMECTOL contains the following inactive ingredients: microcrystalline cellulose, pregelatinised maize starch, magnesium stearate, butylated hydroxyanisole and citric acid.

6.2 INCOMPATIBILITIES

Incompatibilities were either not assessed or not identified as part of the registration of this medicine.

6.3 SHELF LIFE

The expiry date can be found on the packaging. In Australia, information on the shelf life can be found on the public summary of the ARTG.

6.4 SPECIAL PRECAUTIONS FOR STORAGE

Store below 30°C.

6.5 NATURE AND CONTENTS OF CONTAINER

The tablets are packaged in aluminum foil blister packs in cardboard cartons. Pack size: 4 tablets.

6.6 SPECIAL PRECAUTIONS FOR DISPOSAL

In Australia, any unused medicine or waste material should be disposed of by taking to your local pharmacy.

6.7 PHYSICOCHEMICAL PROPERTIES

Ivermectin is a white to yellowish-white non-hygroscopic crystalline powder which is practically insoluble in water, freely soluble in methanol, and soluble in 95% ethanol.

Molecular Formulae:	Molecular Weight:		
22, 23-dihydroavermectin B_{1a}	$C_{48}H_{74}O_{14}$	($R=C_2H_5$)	875.10
22, 23-dihydroavermectin B_{1b}	$C_{47}H_{72}O_{14}$	($R=CH_3$)	861.07

Ivermectin contains a minimum of 90% of 22, 23-dihydroavermectin B_{1a} (where the R group is ethyl) and a maximum of 10% of 22, 23-dihydroavermectin B_{1b} (the R group is methyl).

Chemical structure

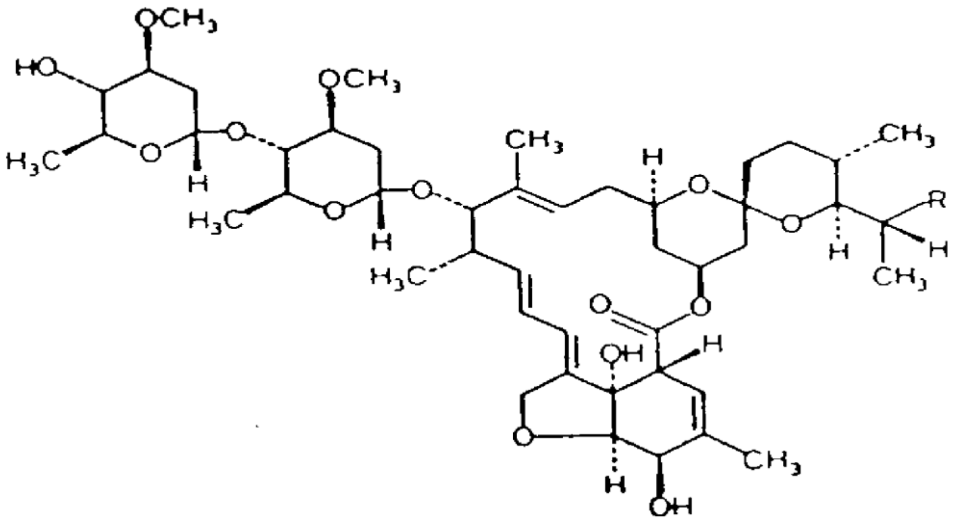

CAS number 70288-86-7

7 MEDICINE SCHEDULE (POISONS STANDARD)

Prescription Only Medicine (S4)

8 SPONSOR

Merck Sharp & Dohme (Australia) Pty Limited
Level 1, Building A, 26 Talavera Road Macquarie
Park NSW 2113 www.msd-australia.com.au

9 DATE OF FIRST APPROVAL

27 August 1999

10 DATE OF REVISION

22 July 2021

11 REFERENCES

Bachewar N P, et al. Comparison of safety, efficacy, and cost effectiveness of benzyl benzoate, permethrin, and Ivermectin in patients of scabies. Indian J Pharmacol. Feb 2009;41(1):9-14.
Chouela E N, et al. Equivalent therapeutic efficacy and safety of Ivermectin and lindane in the treatment of human scabies. Archives of Dermatology. 1999;135(6):651-5.
Ly F, et al. Ivermectin versus benzyl benzoate applied once or twice to treat human scabies in Dakar, Senegal: a randomized controlled trial. Bull World Health Organ. 2009;87:424-30.
Macotela-Ruiz E, et al. The treatment of scabies with oral ivermecrin [Tratamiento de la escabiasis con ivermectina por via oral]. Gaceta Medica de Mexico 1993;129(3):201-5.
Madan V, et al. Oral Ivermectin in scabies patients: A comparison with 1% topical lindane lotion. The Journal of Dermatol. 2001;28(9):481-84.
Mushtaq A, et al. Comparison of efficacy and safety of oral Ivermectin with topical permethrin in treatment of scabies. Journal of Pakistan Association of Dermatologists. 2010;20:227-31.
Nnoruka EN, et al. Successful treatment of scabies with oral Ivermectin in Nigeria. Tropical Doctor. 2001;31(1):15-18.

Strong M, Johnstone P. Interventions for treating scabies (Review). The Cochrane Collaboration. 2010, Issue 10.
Usha V, et al. A comparative study of oral Ivermectin and topical permethrin cream in the treatment of scabies. J Am Acad Dermatol. 2000;42(2)(Part1):236-40.

SUMMARY TABLE OF CHANGES

Section Changed	Summary of new information
ALL	Reformat of PI
4.4, 4.8, 4.9	Added neurological toxicity including depressed level of consciousness and coma<br><br>Added recommended dosages statement

S-IPC-MK0933-T-032021
RCN000019992-AU

Appendix 2A: Number of Tablets of Ivermectin (each tablet being 3mg Ivemectin) given according to weight.

Ivermectin dose is 200ug/kg.

**Weight**<br>**(Participant weight is rounded up to the nearest kg)**	**Number of Tablets**
**Patients <45kg and > 120kg are excluded from this trial**	
45-50	3
51-65	4
66-79	5
80-91	6
92-105	7
106-120	8

Appendix 2 B: Tablet Instruction Sheet (Unapproved RAT kits)

BEFORE TAKING TABLETS read all these instructions carefully. Following these instructions will reduce your risks and will improve the smooth and accurate running of the study

During this study you will be asked to do 2 sets of tasks

1) Tasks required by the government and
2) Tasks required by the study

These tasks are summarized on this page to give you an overview.
The additional pages fill in important details and must all be read before taking the tablets.

Government Tasks
1) If you develop symptoms of Coronavirus infection, you must go for a PCR
2) If you do NOT develop symptoms of Coronavirus infection,
then you must use a TGA (government) approved RAT kit on day 6 of close contact (counting day of close contact as day 0).

The RAT kits we have given you are NOT government or TGA approved. They are only for research. If you cannot obtain a TGA or government approved RAT kit for Day 6 testing after close contact, go to a PCR testing center and ask them to give you one.

If your Day 6 after close contact RAT is positive, you must register on the government website (insert website), phone your GP and phone the health department on 1800 675 398.
If your Day 6 after close contact RAT is not reliable you must obtain another or go for PCR.

Study Tasks
1) Pregnancy test: Woman under 60 must first use the pregnancy test.
Do NOT take the tablets if you are pregnant.

2) Study rapid antigen test kits (manufactured by PCL)
These are saliva RAT kits. You should use them as follows

1. Before you take the tablets: and do NOT take the tablets if the test is positive or unreliable.
2. On EACH of the 1st, 2nd, 3rd and 4th days AFTER taking study tablets
(counting the day of tablets as Day zero for that purpose)
3. On day 14 of your close contact
(counting the day of your close contact as Day zero for that purpose)

In total you will do 7 RATs:
1 TGA approved RAT for the government on day 6 of close contact that you will obtain and
6 study saliva RATs that we have given you.

3) Symptom questionnaires: Please fill in the brief questionnaire each night before bed.

STUDY RAT kits (made by PCL):
You have 7 copies of this page.
Use a fresh page each time you perform a RAT.

You will need to read the separate RAT instructions sheet carefully.
Make sure you read the sheet that is appropriate for your particular RAT kit.
Read that sheet each time you use a RAT to remind you and improve accuracy.

Watch the Youtube video or QR linked video before using a RAT kit the first time
The Youtube link for the saliva kits we have given you is:

BEFORE performing the RAT, complete the information requested below on this sheet. Do NOT eat, drink or smoke for 30 minutes prior to doing the test.

When you use the RAT place the **testing cassette on the rectangle below.**

After the RAT, photograph this whole page and email to (insert CRO trial email address).

IF YOUR FIRST RAT IS POSITIVE, or if it is **not reliable** (for example if the positive control did not work), then **DO NOT TAKE the trial tablets.**

AND if any saliva RAT during the study is positive or not reliable, you must immediately go for a PCR throat swab at a public testing center.

If you develop symptoms at any time during the study**, DO NOT perform a RAT.** Instead y**ou must immediately go for a PCR** throat swab at a public testing center**.**

Name:

Date of birth:

Date of this test:

Time of this test:

When using testing cassette,
place it on the rectangle

Photograph and email this page after each RAT to the email address above

PREGNANCY TEST KIT

WOMEN UNDER 60 YEARS MUST FIRST USE THE PREGNANCY TEST KIT.

Please read the instructions inside the pregnancy test kit carefully before use

If you are pregnant do NOT take the tablets.

If you are pregnant, you must immediately telephone your GP and explain that you are a close contact of a person with Coronavirus, are pregnant, having just returned a positive result on a urine pregnancy test, and whether or not you are in isolation.

If you are pregnant, you must also immediately telephone the public health authority on (insert phone number) and explain that you are a close contact of a person with Coronavirus, are pregnant, having just returned a positive result on a urine pregnancy test, and whether or not you are in isolation.

When you have completed the pregnancy test,

Photograph the result for our records.

Email that photo to our study email (insert email).

Include your **name** with that email so we can record the result against the correct participant.

TAKING THE TABLETS

Women under 60 first do the pregnancy test and
All participants first do a RAT.

Only take tablets if these tests are negative.

Wait at least 2 hours since your last meal,
Then take all the tablets with water.
You should not eat your next meal for at least 1 hour after taking the tablets.

IF YOU DO NOT TAKE THE TABLETS
Any unused tablets should be disposed in the rubbish in a way that children or pets cannot access them. Do NOT return any tablets.

Appendix 2 C: Tablet Instruction Sheet (Approved RAT kits)

Tablet instruction sheet

BEFORE TAKING TABLETS read all these instructions carefully. Following these instructions will reduce your risks and will improve the smooth and accurate running of the study

During this study you will be asked to do 2 sets of tasks

1) Tasks required by the government and
2) Tasks required by the study

These tasks are summarized on this page to give you an overview.
The additional pages fill in important details and must all be read before taking the tablets.

<u>Government Tasks</u>
1) If you develop symptoms of Coronavirus infection, you must go for a PCR
2) If you do NOT develop symptoms of Coronavirus infection, then you must use a RAT kit on day 6 of close contact (counting day of close contact as day 0).

The RAT kits we have given you are only for this study

If your Day 6 after close contact RAT is positive, you must register on the government website (https://www.coronavirus.vic.gov.au/report), phone your GP and phone the health department on 1800 675 398.
If your Day 6 after close contact RAT is not reliable you must obtain another or go for PCR.

<u>Study Tasks</u>
1) Pregnancy test: Woman under 60 must first use the pregnancy test.
Do NOT take the tablets if you are pregnant.

2) Study rapid antigen test kits (manufactured by PCL)
These are saliva RAT kits. You should use them as follows

1. Before you take the tablets: and do NOT take the tablets if the test is positive or unreliable.
2. On EACH of the 1st, 2nd, 3rd and 4th days AFTER taking study tablets
(counting the day of tablets as Day zero for that purpose)
3. On days 6 and 14 of your close contact
(counting the day of your close contact as Day zero for that purpose)

In total you will do 7 study saliva RATs that we have given you.

3) Symptom questionnaires: Please fill in the brief questionnaire each night before bed.

You will need to read the rapid antigen test kit instructions carefully and to also watch the company instructional video on https://protect-au.mimecast.com/s/pnkdCYWLOxhLMW4Vrh08iOa?domain=youtube.com. .
Please note that you should not eat, drink or smoke for 30 minutes prior to doing the test.

STUDY RAT kits (made by PCL):
You have 7 copies of this page.
Use a fresh page each time you perform a RAT.

You will need to read the separate RAT instructions sheet carefully.
Make sure you read the sheet that is appropriate for your particular RAT kit.
Read that sheet each time you use a RAT to remind you and improve accuracy.

Watch the Youtube video or QR linked video before using a RAT kit the first time

The Youtube link for the saliva kits we have given you is: https://protect-au.mimecast.com/s/pnkdCYWLOxhLMW4Vrh08iOa?domain=youtube.com

BEFORE performing the RAT, complete the information requested below on this sheet. Do NOT eat, drink or smoke for 30 minutes prior to doing the test.

When you use the RAT place the **testing cassette on the rectangle below.**

After the RAT, photograph this whole page and email to (ivemsq@florey.edu.au).

IF YOUR FIRST RAT IS POSITIVE, or if it is **not reliable** (for example if the positive control did not work), then **DO NOT TAKE the trial tablets.**

AND if any saliva RAT during the study is positive or not reliable, you must immediately go for a PCR throat swab at a public testing center.

If you develop symptoms at any time during the study**, DO NOT perform a RAT.** Instead y**ou must immediately go for a PCR** throat swab at a public testing center**.**

Name:

Date of birth:

Date of this test:

Time of this test:

When using testing cassette,

place it on the rectangle

PREGNANCY TEST KIT

Women UNDER 60 YEARS must first use the pregnancy test kit.

Please read the instructions inside the pregnancy test kit carefully before use

If you are pregnant do NOT take the tablets.

If you are pregnant, you must immediately telephone your GP and explain that you are a close contact of a person with Coronavirus, are pregnant, having just returned a positive result on a urine pregnancy test, and whether or not you are in isolation.

If you are pregnant, you must also immediately telephone the public health authority on 1800 675 398 and explain that you are a close contact of a person with Coronavirus, are pregnant, having just returned a positive result on a urine pregnancy test, and whether or not you are in isolation.

When you have completed the pregnancy test,

Photograph the result for our records.

Email that photo to our study email (ivemsq@florey.edu.au).

Include your name with that email so we can record the result against the correct participant.

TAKING THE TABLETS

Women under 60 first do the pregnancy test and
All participants first do a RAT.

Only take tablets if these tests are negative.

Wait at least 2 hours since your last meal,
Then take all the tablets with water.
You should not eat your next meal for at least 1 hour after taking the tablets.

IF YOU DO NOT TAKE THE TABLETS
Any unused tablets should be disposed in the rubbish in a way that children or pets cannot access them. Do NOT return any tablets.

Appendix 2D: Trial clinical contact card

Participants will each receive a trial clinical contact card in the bag dispensed by pharmacy.

The text of the card will be as follows

Keep this card with you during the study

This trial is gathering research information but is not providing medical care

If you develop any symptom, if you feel it is an emergency call an ambulance immediately on (insert country specific ambulance phone number).

If you don't feel it is an emergency, you must telephone your usual general practitioner and remind the doctor you are a close contact of a person with Coronavirus.

You must also telephone your local public health agency on this 24 hour telephone number (insert country specific number).

If doctors or health care workers wish to speak with one of our trial doctors, then please ask them to phone (insert trial clinical contact phone number).

Appendix 3: Table indicating actual dose (ug/kg) of Ivermectin

Table indicating actual dose (ug/kg) of Ivermectin that participants in the treatment arm will each receive (according to their weight, rounded up to the nearest kg). The dose for weights up to 80kg is based on the recommended number of tablets for a 200ug/kg oral dose listed in the Stromectol Product Information, Appendix 1 (Stromectol is the form of Ivermectin tablet marketed in Australia). The Stomectol Product Information does not list the exact number of tablets to be administered for weights over 80kg but stipulates a dose of "Approx 200ug/kg". For a weight above 80kg, the dose calculation was to round weight up to the nearest kg and round Ivermectin tablet number up to the nearest tablet. In some participants, the dose will be higher than 200ug/kg. However, if for a given weight, the actual dose in ug/kg then exceeded by over 15% a dose of 200ug/kg, the tablet number was then rounded down.

Weight (kg)	Number of tablets for a treatment dose	Actual dose (ug/kg)
45	3	200
46	3	196
47	3	191
48	3	188
49	3	184
50	3	180
51	4	235
52	4	231
53	4	226
54	4	222
55	4	218
56	4	214
57	4	211
58	4	207
59	4	203

60	4	200
61	4	197
62	4	194
63	4	190
64	4	188
65	4	185
66	5	227
67	5	224
68	5	221
69	5	217
70	5	214
71	5	211
72	5	208
73	5	205
74	5	203
75	5	200
76	5	197
77	5	195
78	5	192
79	5	190
80	6	225
81	6	222
82	6	220

83	6	217
84	6	214
85	6	212
86	6	209
87	6	207
88	6	205
89	6	202
90	6	200
91	6	198
92	7	228
93	7	226
94	7	223
95	7	221
96	7	219
97	7	216
98	7	214
99	7	212
100	7	210
101	7	208
102	7	206
103	7	204
104	7	202
105	7	200

106	8	226
107	8	224
108	8	222
109	8	220
110	8	218
111	8	216
112	8	214
113	8	212
114	8	211
115	8	209
116	8	207
117	8	205
118	8	203
119	8	202
120	8	200

Appendix 4: Symptom questionnaire

Instructions

NB: This trial is gathering research information but is not providing medical care.

If you develop any symptom, if you feel it is an emergency call an ambulance immediately on (insert country specific ambulance phone number).

If you don't feel it is an emergency, you must telephone your usual general practitioner and remind the doctor you are a close contact of a person with Coronavirus.

You must also telephone your local public health agency on this 24 hour telephone number (insert country specific number).

You will need to have a repeat test for Coronavirus if you experience any symptoms of Coronavirus (such as: fever, new cough, sore throat. runny nose, loss of smell, loss of taste or more difficulty breathing than usual).

If at any stage during this study you receive a positive result for a Coronavirus test, you must immediately telephone the public health authority on [insert phone number] to confirm they are aware of that result and to ask what care is required and then you must telephone your GP to confirm your GP is aware of that result and to discuss your health care.

Answer the questionnaire every day, just before you go to bed.

Circle Yes or No to record whether you had each symptom that day.

If you forget a day, catch up first thing the next morning.

At the end of 7 days, take a photo of the page and email it to (insert CRO trial email address).

Your name: ________________

When did you start to answer this questionnaire: Day:_________ Date: _____

Symptom	*Monday*	*Tuesday*	*Wednesday*	*Thursday*	*Friday*	*Saturday*	*Sunday*
**Fever**	Yes/No	Yes/No	Yes/No	Yes/No	Yes/No	Yes/No	Yes/No
**New Cough**	Yes/No	Yes/No	Yes/No	Yes/No	Yes/No	Yes/No	Yes/No
**Sore throat**	Yes/No	Yes/No	Yes/No	Yes/No	Yes/No	Yes/No	Yes/No
**Runny nose**	Yes/No	Yes/No	Yes/No	Yes/No	Yes/No	Yes/No	Yes/No
**Loss of smell**	Yes/No	Yes/No	Yes/No	Yes/No	Yes/No	Yes/No	Yes/No
**Loss of taste**	Yes/No	Yes/No	Yes/No	Yes/No	Yes/No	Yes/No	Yes/No
**More trouble breathing than usual**	Yes/No	Yes/No	Yes/No	Yes/No	Yes/No	Yes/No	Yes/No
**List any other symptoms you don't usually have, which you had that day**							

For any symptoms you don't usually have which you listed in the last row, grade them by writing in brackets under each: (mild), (moderate) or (severe)

Appendix 5: Proforma Letter to General Practitioner/Primary Care Physician

Insert Name of general practitioner/primary care physician
Insert Address of general practitioner/primary care physician

Insert Date

Dear Dr (insert name of general practitioner/primary care physician)

Re: Insert name and address of participant

(Insert participant name) has advised us that he/she has been a close contact of a person infected with SARS-CoV-2 (Coronavirus).

We are conducting a randomized controlled trial to see whether it is possible to prevent Coronavirus infection after someone had been a close contact of an index case.

(Insert participant name) has consented to participate in this trial and we have prescribed him/her a single oral dose of Ivermectin 200ug/kg or Placebo.

(Insert participant name) has identified (insert general practitioner/primary care physician name) as his/her general practitioner/primary care physician.

Whilst the trial will be gathering follow up data by telephone, **the trial is not clinically examining or monitoring (inset participant name) and we leave you to clinically monitor (insert participant name) who has been exposed to Coronavirus.**

Information about the trial is also available on the Australian New Zealand Clinical Trial Registry www.anzctr.org.au . On that website one can use the search function and enter the trial registry number ACTRN12621001535864p.

Information about Ivermectin is also available from the product information in MIMS or in your prescribing software.
Please may we draw to your attention the exclusion criteria for our trial.
Insert name of participant has advised us that the following do not apply to (insert him or her).
If you are aware that your patient (insert name of participant) has one of these exclusion criteria please contact one of the doctors associated with this trial on 0493079326.

List of exclusion criteria:

The close contact with an infectious index case of SARS-CoV-2 occurred in a hospital

- Not agreeable to being at home for delivery of the investigational product
- Residing outside the current geographic recruitment area
- The index case who has SARS-CoV-2 lives in the same residence as the potential participant.

- Another person who lives in the same residence as the potential participant has returned a positive pharyngeal PCR or RAT for SARS-CoV-2 in the last 2 weeks.
- Unable to provide the name, address and phone number of the potential participant's general practitioner/primary care physician OR does not have such a general practitioner/primary care physician.
- Has not attended a doctor at the practice of the above general practitioner/primary care physician for more than 12 months. (NB Given the pandemic, that attendance may have been by telehealth).
- Lives alone (as potentially at higher risk should there be a serious adverse event).
- Unable to provide the name and phone number of a back-up contact person.
- History of past infection with SARS-CoV-2.
- Use of Ivermectin for any purpose in 5 weeks prior to enrolment.
- Known past allergy or severe adverse reaction to Ivermectin.
- Weight <45kg or > 120kg.
- Pregnant or breast feeding.
- Not willing to avoid pregnancy during the course of the research and for a period of 6 months after completion of the research project
- Not willing to refrain from falling pregnant or fathering a child for 6 months after last dose of investigational product.
- Cirrhosis or known decompensated liver disease (Child-Pugh B or C).
- Current use, or use within the last 3 months, of the drug amiodarone.
- Current use of any of the following drugs: warfarin, verapamil, diltiazem, quinidine, spironolactone, ciclosporin, tacrolimus, cobicistat, indinavir, ritonavir, didanosine (DDI), ketoconazole, itraconazole, fusidic acid, erythromycin, clarithromycin.
- Past sedation or somnolence from products containing codeine as may be a marker for risk for rare serious adverse effect from Ivermectin.
- History of residency or travel to loa loa endemic areas ("Angola, Cameroon, Central African Republic, Chad, Democratic Republic of Congo, Ethiopia, Equatorial, Guinea, Gabon, Republic of Congo, Nigeria and Sudan", Chaccour et al 2020b).
- Severe Asthma (as Stromectol product information notes in "Post-marketing Experience" "very rarely,…worsening of bronchial asthma"
- Encephalopathy.
- Head injury requiring medical attention in the last 6 months.
- Concussion within the last 6 months.
- Fit, seizure, stroke, TIA (transient ischaemic attack) or transient global amnesia in the last 6 months.
- History of epilepsy (as Stromectol product information notes in "Post-marketing Experience" "very rarely,…seizures)
- Dementia of any type.
- Not usually fully independent in activities of daily living and self-care, including: washing, toileting, dressing and dental care.
- Inability of participant or person responsible to communicate to the level necessary to provide verbal or written consent.
- Incarcerated by local, state or federal authorities.
- Conditions which in the opinion of the investigative team would make successful trial

completion (including follow up data collection) unlikely, for example including uncontrolled substance use, poorly controlled mental state disorder.

- Unable to advise trial staff of Coronavirus vaccination status including date of administration of last vaccine dose.

(NB past vaccination against Coronavirus is NOT an exclusion as whilst it may reduce disease severity it may not prevent infection. However, randomization stratifies by Coronavirus vaccination status, knowledge of which is thus a prerequisite for the protocol)

- Already enrolled in another Coronavirus RCT
- In Australia, lack of a valid Medicare Card
- Unable or unwilling to have a RAT upon receipt of investigational product, on Days 1, 2, 3, 4 and 5, and on days 6 and 14 following close contact.
- Unable or unwilling to have a pharyngeal swab PCR test for SARS-CoV-2 at 6 and/or 14 days post initial exposure to a close contact if such tests are then practical and available given the then public health circumstances.

If you wish further information about this trial or wish to speak with a doctor associated with the trial, please telephone 0493079326..

If you know of other close contacts of an index case who may wish to consider joining this trial, you may wish to direct them to the trial website (insert website).

Yours sincerely,
The Trial Investigators:
Dr Mark Stein
Kylie Wagstaff
Professor David Jans
Professor Joseph Torresi

Appendix 6: Child-Pugh classification of severity of cirrhosis

**Parameter**	**Points assigned**		
	**1**	**2**	**3**
Ascites	Absent	Slight	Moderate
Bilirubin	<2 mg/dL (<34.2 micromol/L)	2 to 3 mg/dL (34.2 to 51.3 micromol/L)	>3 mg/dL (>51.3 micromol/L)
Albumin	>3.5 g/dL (35 g/L)	2.8 to 3.5 g/dL (28 to 35 g/L)	<2.8 g/dL (<28 g/L)
Coagulation			
Use Prothrombin time prolongation (in seconds)	<4	4 to 6	>6
OR Use INR	<1.7	1.7 to 2.3	>2.3
Encephalopathy	None	Grade 1 to 2	Grade 3 to 4

Modified Child-Pugh classification of the severity of liver disease according to the degree of ascites, the serum concentrations of bilirubin and albumin, the coagulation and the degree of encephalopathy. A total Child-Turcotte-Pugh score of 5 to 6 is considered Child-Pugh class A (well-compensated disease), 7 to 9 is class B (significant functional compromise), and 10 to 15 is class C (decompensated disease). These classes correlate with one- and two-year patient survival: class A: 100 and 85%; class B: 80 and 60%; and class C: 45 and 35%.
INR: international normalized ratio.
