# Supplementary material for: A Pilot, Randomised, Placebo-Controlled, Double-Blind Trial of a Single Oral Dose of Ivermectin for Post-Exposure Prophylaxis of SARS-CoV-2
Source: Pharmaceutics. 2025 Sep 16;17(9):1205. doi: 10.3390/pharmaceutics17091205 (PMC12473430; doi:10.3390/pharmaceutics17091205)
Supplement: Supplementary file 1 [file pharmaceutics-17-01205-s001.zip › Supplementary Material S2.pdf]

Supplementary Material S2

The following Pages are the Statistical Analysis Plan for the trial presented exactly as per the pre-analysis submission to both Belberry Human Research Ethics Committee and the Data Safety Monitoring Board.

Ivermectin Trial

A pilot randomized placebo-controlled double-blind trial of single dose oral Ivermectin for post-exposure prophylaxis of SARS-CoV-2

Statistical Analysis Plan

Protocol Number: EP-Ivermectin-01

Version 4.1

Date: 1st July 2024

Author: Alan Herschtal

(with contributions from Mark Stein, David Jans and Kylie Wagstaff)

Contents

1. INTRODUCTION 4
2. STATISTICAL CONSIDERATIONS & OBJECTIVES 4
2.1. GENERAL CONSIDERATIONS 4
2.2. MISSING DATA 4
2.2.1. SPECIFIC RAT/PCR TESTS NECESSARY FOR DETERMINATION OF CONVERSION STATUS 4
2.3. PRIMARY OBJECTIVE 5
2.3.1. PRIMARY ANALYSIS 5
2.3.2. RANDOMIZATION 6
2.3.3. METHODS OF ANALYSIS 6
2.4. SECONDARY OBJECTIVES 9
2.5. SURVIVAL CURVE 11
2.5.1 Subgroup Analysis 11
2.6. EXPLORATORY OBJECTIVE 12
2.7. SENSITIVITY ANALYSES 12
2.7.1. FINAL ANALYSIS OF PRIMARY AND SECONDARY ENDPOINTS 12
2.7.2. FOLLOW UP ANALYSIS 12
3. TABLE SHELLS 14
4. REFERENCES 19

1. Introduction

This document describes the Statistical Analysis Plan (SAP) for a randomized placebo-controlled double-blind trial of single dose oral Ivermectin for post-exposure prophylaxis of SARS-CoV-2. The background, trial design (including randomisation), eligibility criteria, endpoint definitions and sample size calculation for this trial may be found in the corresponding Protocol document, version 10.0, dated 2nd February 2023. All references to "the Protocol" should be taken as referring to this version.

In the event of conflict between this SAP and the Protocol, the SAP will take precedence.

2. Statistical considerations & objectives

2.1. General considerations

The definitive analyses for both the primary and secondary objectives will be based on the intention-to-treat (ITT) principle. For ITT analyses, subject to the availability of the data necessary for the given endpoint (see the section on missing data below) all eligible participants who consumed any trial tablet will be included and analysed in accordance with the treatment arm to which they were accrued.

As screening was effectively completed after randomisation (due to the public health circumstances of this pandemic trial) with a mandatory RAT immediately prior to taking IP, any participant who took IP without performing the mandatory preceding RAT was ineligible to take IP and hence is not included in the ITT.

2.2. Missing Data

If a participant missed one of the specific RATs/PCRs defined in section 2.2.1 below, it will be considered that an assessment of negative conversion status cannot be relied upon. This missing response value will not be imputed. To avoid possible bias, all such participants will be considered to have a missing response value at that timepoint for the relevant primary and secondary objectives, regardless of whether or not one of the RATs/PCRs they subsequently performed yielded a positive result.

2.2.1. Specific RAT/PCR Tests necessary for determination of conversion status

The RAT/PCR tests which are considered essential to the determination of conversion status are as follows.

- a pharyngeal swab PCR or a TGA-approved RAT or a trial RAT on Day 14 post close contact, and
- a pharyngeal swab PCR or a TGA-approved RAT on Day 6 post close contact and
- at least three out of four trial RATs from the first four days following IP

Participants missing an essential RAT/PCR test will be excluded from all analyses. The exceptions to this rule are: i) that participants who return a positive RAT/PCR test and then do not perform a subsequent 'essential' test will be included in the analysis and considered to have converted, since the only reason for missing the essential test was that it was unnecessary, given that the participant already returned positive on a previous test and ii) that participants who performed a RAT prior to IP but then did not perform three out of four trial RATs from the first four days following IP would be included in the analysis if they performed a PCR at Day 6 post close contact and a PCR or TGA-approved RAT or trial RAT at Day 14 post close contact because the PCR at Day 6 post close contact would give confidence of conversion status (negative or positive) up to that time point.

In addition, for the proportion estimation method of the primary endpoint (see below), all eligible participants who returned a positive RAT/PCR test at any time point will be included.

The number of potential participants screened, the number excluded and the reasons for exclusion, the number randomized to each arm, the number lost to follow up and/or withdrawn (and the reason for any withdrawal) and the number missing specific RAT/PCR tests (above) will be reported per arm in tabular form.

2.3. PRIMARY OBJECTIVE

2.3.1. PRIMARY ANALYSIS

The primary objective is to test whether Ivermectin 200 ug/kg administered orally on Day 1 after exposure to SARS-CoV-2, is superior to placebo as post-exposure prophylaxis for SARS-CoV-2. Note that all references to Day numbers in this SAP are relative to the Day of taking IP, which is to be considered as Day 1 . The Day of taking IP may be different from the day of close contact. Participants were eligible for the trial if they had had close contact with a person infectious with SARS-CoV-2 in the preceding 72 hours at the time of assessment for eligibility.

2.3.2. Randomization

Participants were randomized with an allocation ratio of 1:1 between the Ivermectin and placebo arms. Randomization was stratified by:

- Coronavirus vaccination status, with strata defined by the pairwise combination of the participant's vaccination count, being the number of previous vaccinations (0 – 6), and the participant's vaccination recency, being the amount of time passed since the most recent vaccination (<10 days, between 10 days and 6 months, or greater than 6 months for vaccinations 1 – 2 or <10 days versus >=10 days for vaccinations 3-6). All possible combinations of vaccination count and vaccination recency were separate strata in the vaccination status. If a participant fulfilled more than one category, that participant was allotted to the last category in the above list which that participant fulfilled.
- exposure site, with strata being i) a home, ii) an indoor work environment, iii) a family gathering or a social or a religious function or a ceremony each being of less than 30 people.

2.3.3. Methods of Analysis

The primary objective will be analysed using the following methods:

<u>Proportion estimation method</u>

Considering all participants who convert to a positive PCR or positive TGA-approved RAT for SARS-CoV-2 by the close of recruitment (May 31st 2024), the estimate and 95% CI (using the Wilson score method[1]) of the proportion who received Ivermectin will be provided. A p-value will be provided for the hypothesis test with null hypothesis that the proportion is 0.5.

The rationale behind the proportion estimation method of analysis is as follows. If Ivermectin has no benefit for post-exposure prophylaxis following close contact with an infectious index case of SARS-CoV-2, one would expect that the treatment arm allocation ratio in the participants who convert to a positive pharyngeal swab PCR or RAT for SARS-CoV-2 should reflect the overall allocation ratio. Since the overall allocation ratio is 1:1, if Ivermectin has no benefit, it can be expected that half of the converted participants will have received Ivermectin and half will have received placebo. The proportion of those participants who convert to a positive pharyngeal swab PCR or RAT for SARS-CoV-2, who received Ivermectin, can thus be tested as to whether it is different from 0.5, and this used as a test for whether Ivermectin has benefit.

Low-dimensional Logistic Regression

To complement the proportion estimation method above, a logistic regression model will be constructed with treatment arm (Ivermectin vs. placebo) as the independent variable and Conversion status (positive finding on PCR or TGA-approved RAT within 14 days following close contact vs. no positive finding on PCR or TGA-approved RAT within 14 days following close contact) as the Boolean-valued dependent (response) variable. All participants who had completed 14 days following close contact by the close of recruitment (May 31st 2024) and had sufficient RAT/PCR tests as per section 2.2.1 will be included in this analysis. The variables stratified for in the randomization will then be controlled for as potential confounders in the model by including them as additional independent variables.

Firth's bias adjustment method[2-4], which removes the first-order term from the asymptotic bias of maximum likelihood estimators such as are used in logistic regression, will be used to mitigate the likely problem of near or complete separation which is likely to occur in logistic regression models with small sample sizes and/or strong effects.

To mitigate the problem of omitted variable bias (unmeasured heterogeneity) [6] in the calculation of odds ratios from logistic regression models, results will be reported in the form of the 'average treatment effect'[7]. This is the average change across all participants included in the analysis set for the primary objective, in the probability of conversion to positivity predicted by the model, when the exposure changes from placebo to Ivermectin.

The average treatment effect and its 95% CI will be reported. P-values for the hypothesis test with null hypothesis of no association (change in the predicted probability of conversion to positivity = 0) will also be reported.

High-dimensional Logistic Regression

In order to allow for the possibility of adjusting for confounders other than the stratification variables, a high-dimensional binary logistic regression model will also be constructed to address the same objective. The inclusion criteria, the dependent variable (Conversion status) and the independent variable (treatment arm) for this analysis will be the same as those for the low-dimensional logistic regression above. Additional confounding variables will be controlled for by incorporating them into the model as additional independent variables. The confounder variables currently planned for inclusion appear below, however this list may be reviewed and changed at any time prior to analysis. The final decision as to what confounding variables to

control for, as well as any transformations to apply to them, will be made based on analysis of blinded pooled participant data[i], prior to performing any of the study analyses[8].

As for the low-dimensional logistic regression model above, Firth's bias adjustment method[2,3] will be used to mitigate the likely problem of near or complete separation and results will be reported in the form of the 'average treatment effect'[7] (point estimate and 95% CI). P-values for the hypothesis test with null hypothesis of no association (change in the predicted probability of conversion to positivity = 0) will also be reported.

High-dimensional Logistic Regression Confounding Variables

The following is the initially proposed list of confounders to be controlled for in the high-dimensional logistic regression model. As stated above, this list may be modified at any time prior to analysis. This list of confounders will also be controlled for in analysis of the 4th secondary objective.

All factor valued confounders in this list will be dummy coded using step functions in order to avoid any assumption of an equal difference between consecutive factor levels.

A) Vaccination status: a binary indicator which is positive if the individual has had at least 2 vaccines and the most recent one was > 10 days but within 6 months prior to the date of accrual.
B) Location of close contact: home, work, social
C) Isolation status at day 0: A four-level dummy variable with the following levels: not isolating; in isolation with someone else positive for SARS-CoV-2; in isolation with another close contact of a case of SARS-CoV-2; or isolating with no-one else positive for or a close contact of a case of SARS-CoV-2
D) Age
E) Prior infection with Coronavirus
F) Inhaled glucocorticoid (dose per day)
G) Vitamin D (dose per day)
H) ACE inhibitor usage (yes/no)
I) Time interval between close contact and receipt of IP (Ivermectin or placebo)

[i] The statistician may be granted access to unblinded data for the purpose of calculating arm specific summary statistics, but these statistics will only be presented in blinded form to the study team for the purpose of assessing inclusion of confounders in the model.

J) Oral glucocorticoid use at baseline

Model Goodness of Fit

Goodness of fit of the logistic regression will be assessed graphically by sorting the data by predicted risk, splitting into quintiles and then graphing the observed risk against the predicted risk for each quantile. This will be done for both the low-dimensional and the high-dimensional logistic regression models.

Primary Objective Conclusions

If the high-dimensional logistic regression converges, and satisfies the goodness of fit assessment, conclusions will be based on the high-dimensional logistic regression test, and the proportion test and the low-dimensional logistic regression test will be considered as sensitivity analyses. However, in the event that the high-dimensional logistic regression fails to converge, or fails to satisfy the goodness of fit assessment, conclusions will be based on the low-dimensional logistic regression test.

2.4. SECONDARY OBJECTIVES

The secondary objectives below will be tested amongst participants who completed 14 or 28 days post close contact by the close of recruitment date (May 31st 2024) and who converted to a positive PCR or positive TGA-approved RAT for SARS-CoV-2.

1. To characterise the difference between arms in days alive and free of SARS-CoV-2 symptoms until Day 14.
2. To characterise the difference between arms in days alive and free of SARS-CoV-2 symptoms until Day 28.
3. To characterise the difference between arms in days alive and free of presentation to hospital, acute hospital care and outpatient care under hospital supervision until Day 28.
4. To characterise the difference between arms in time (days) from exposure to an index case of SARS-CoV-2 until the first positive PCR or positive TGA-approved RAT for SARS-CoV-2.

The symptoms to be considered as SARS-CoV-2 symptoms for the purpose of the secondary objectives above are as follows: fever, new cough, sore throat, rhinorrhoea, loss of smell, loss of taste, headache, more difficulty breathing than usual.

For each of the symptoms considered separately, the number of days alive and free of the symptom until Day 28 will be displayed graphically in a histogram for each treatment arm separately.

For the secondary objectives numbered 1 – 4 above, analysis will proceed as follows.

A beta binomial regression model[9] will be constructed with the number of days (i.e. either the number of days alive and free of SARS-CoV-2 symptoms, or the number of days free of presentation to hospital, acute hospital care and outpatient care under hospital supervision, or the number of days until the first positive test) as the response variable (dependent variable) and the treatment arm as the exposure variable (independent variable). The overdispersion parameter of the beta binomial model will be assumed constant across arms in modelling. Potential confounders to be controlled for are discussed below.

For each secondary objective, the beta binomial regression models will be used to estimate the average treatment effect across participants in the analysis set. The treatment effect for an individual participant is defined as the model predicted number of days for that participant if treatment was Ivermectin, minus the model predicted number of days for that participant if treatment was placebo. The point estimate of the average treatment effect, its 95% CI, and the p-value for the hypothesis test with a null hypothesis of zero difference in average treatment effect will be provided. Models will be constructed using the betabin function in the R package aod[10].

For the first three secondary objectives, below is the initially proposed list of the potential confounders to be controlled for in modelling. The final decision as to what variables to include in this list, as well as any transformations to apply to them will be made following analysis of the blinded pooled participant data[ii], prior to performing any of the study analyses[8].

A) Past Coronavirus infection
B) Vaccination status
C) Time interval between exposure and receipt of IP (Ivermectin or placebo)
D) Age

[ii] As above, the statistician may be granted access to unblinded data in order to calculate arm specific summary statistics, but will present these in blinded form only to the study team for assessing inclusion of confounders.

E) BMI
F) Diabetes (yes/no)
G) Vitamin D (dose/day)
H) Hypertension (yes/no)
I) Lung disease (yes/no)
J) Heart disease (yes/no)
K) Current smoker (yes/no)
L) Aspirin (taking/not taking)
M) Proton pump inhibitor (taking/not taking)
N) HMG CoA Reductase (statin) (taking/not taking)
O) Oral glucocorticoid use at baseline

As for the primary objective, a low-dimensional version of each of the models for the secondary objectives will also be produced. Conclusions will be based on the high-dimensional model if it converges, and on the low-dimensional model otherwise.

The variables to be controlled for in analysis of the 4th secondary objective (among those who convert to a positive PCR or TGA-approved RAT the difference (those who received Ivermectin versus those who received placebo) in time from exposure to an index case of SARS-CoV-2 to a positive PCR or positive TGA-approved RAT for SARS-CoV-2) will be the same as those for the primary objective.

2.5. Survival Curve

A Kaplan-Meier product limit curve will be provided for the time until a positive test.

2.5.1 Subgroup Analysis

Additionally, analyses above will be repeated for sub-groups defined by isolation status on Day 1, where isolation status is one of: i) not isolating; ii) isolating with someone else who has tested positive; iii) isolating with a close contact of a case of SARS-CoV-2 iv) isolating alone or with no-one else who was a close contact of a case of, or tested positive for, SARS-CoV-2.

As for the main primary objective, a low-dimensional version of each of the models for the sub-group analyses will also be produced. Conclusions will be based on the high-dimensional model if it converges, and on the low-dimensional model otherwise.

2.6. Exploratory Objective

An additional, exploratory objective, will be addressed amongst all participants eligible for inclusion in the analysis of the primary objective using the logistic regression method. This objective is to test for a relationship between conversion status (dependent variable) and each of various covariates (independent variables) considered in separate models. Following the same method as for the primary objective, logistic regression models will be built, adjusting for separation using Firth's correction. The average effect of the covariate, its 95% CI and p-value will be reported. All models will be univariate. For multinomial (i.e. categorical) covariates, the average treatment effect will not be reported. For continuous valued covariates, the average treatment effect per unit change in the covariate will be reported. Independent variables to be considered include the confounders which will be used for analysis of the primary endpoint.

2.7. Sensitivity analyses

All trial analyses (both primary and secondary) will be repeated separately using each of the following two eligibility criteria.

1. Excluding participants whose administered Ivermectin dose was <200ug/kg.
2. Excluding participants who experienced symptoms of infection with SARS-CoV-2 between enrolment (consent) and taking their investigational product tablets or who experienced symptoms of infection with SARS-CoV-2 within the first 4 hours after taking their investigational product tablets or who were asymptomatic but returned a positive or an inconclusive result on a rapid antigen test for SARS-CoV-2 within 4 hours after taking the investigational product.

If, for either of these sets of proposed sensitivity analyses, none of the participants are excluded based on these criteria, then that set of analyses will not be performed.

2.7.1. Final analysis of primary and secondary endpoints

All analyses above will be repeated once all participants have reached their 14-day post close contact time point (analyses for the primary endpoint) and 28-day post IP follow-up time point (analyses for the secondary endpoints).

2.7.2. Follow up analysis

After Day 28, participants are followed monthly for 6 months following IP. Where sufficient data exist for meaningful analysis, mortality and admission to hospital will be reported for months 2, 3, 4, 5 and 6 separately and cumulatively and tested for difference between treatment arms. Logistic regression models will be constructed with mortality (or hospital admission) in the time period of interest as the dependent variable and treatment arm as the independent variable of interest. Variables stratified by in the randomization will be controlled for in these models. Where data are insufficient for meaningful analysis, counts of mortality and admission to hospital will be reported by treatment arm descriptively.

Adverse Events (AEs) will be characterized by tabulating the count of AEs until 6 months post accrual per AE type and grade, considering the worst grade for each patient and AE type.

Serious Adverse Events will be listed, together with their relatedness to treatment and outcome.

3. TABLE SHELLS

Table 1: Basic descriptive statistics of baseline demographic and disease characteristics

Characteristic	Statistic	Treatment Group		Overall
		Ivermectin	Placebo	
**Age at Study Entry (years)**	n	xx	xx	xx
	Mean (SD)	xx.x (xx.x)	xx.x (xx.x)	xx.x (xx.x)
	Median [IQR]	xx.x [xx, xx]	xx.x [xx, xx]	xx.x [xx, xx]
**Height* (cm)**	n	xx	xx	xx
	Mean (SD)	xxx.x (xx.x)	xxx.x (xx.x)	xxx.x (xx.x)
	Median [IQR]	xxx.x [xxx, xxx]	xxx.x [xxx, xxx]	xxx.x [xxx, xxx]
**Weight* (kg)**	n	xx	xx	xx
	Mean (SD)	xxx.x (xx.x)	xxx.x (xx.x)	xxx.x (xx.x)
	Median [IQR]	xxx.x [xxx, xxx]	xxx.x [xxx, xxx]	xxx.x [xxx, xxx]
**BMI (kg/m^2)**	n	xx	xx	xx
	Mean (SD)	xx.x (xx.x)	xx.x (xx.x)	xx.x (xx.x)
	Median [IQR]	xx.x [xx, xx]	xx.x [xx, xx]	xx.x [xx, xx]
**Sex**	n	xx	xx	xx
	Male	xx (xx.x%)	xx (xx.x%)	xx (xx.x%)
	Female	xx (xx.x%)	xx (xx.x%)	xx (xx.x%)
**Isolation Status**	n	xx	xx	xx
	:	xx (xx.x%)	xx (xx.x%)	xx (xx.x%)
	:	xx (xx.x%)	xx (xx.x%)	xx (xx.x%)
	:	xx (xx.x%)	xx (xx.x%)	xx (xx.x%)
**Number of prior coronavirus infections**	0	xx (xx.x%)	xx (xx.x%)	xx (xx.x%)
	1	xx (xx.x%)	xx (xx.x%)	xx (xx.x%)
	2	xx (xx.x%)	xx (xx.x%)	xx (xx.x%)
	More than 2	xx (xx.x%)	xx (xx.x%)	xx (xx.x%)
**Coronavirus vaccination status, had… ^**	Zero	xx (xx.x%)	xx (xx.x%)	xx (xx.x%)
	1st vaccination	xx (xx.x%)	xx (xx.x%)	xx (xx.x%)
	2nd <10 days ago	xx (xx.x%)	xx (xx.x%)	xx (xx.x%)
	2nd ≥10 or more days ago	xx (xx.x%)	xx (xx.x%)	xx (xx.x%)
	2nd ≥=10 or more days ago but <=6 months ago	xx (xx.x%)	xx (xx.x%)	xx (xx.x%)
	2nd ≥6 months ago	xx (xx.x%)	xx (xx.x%)	xx (xx.x%)
	3rd < 10 days ago	xx (xx.x%)	xx (xx.x%)	xx (xx.x%)
	3rd ≥10 days ago	xx (xx.x%)	xx (xx.x%)	xx (xx.x%)

	4th <10 days ago	xx (xx.x%)	xx (xx.x%)	xx (xx.x%)
	4th ≥10 days ago	xx (xx.x%)	xx (xx.x%)	xx (xx.x%)
	5th <10 days ago	xx (xx.x%)	xx (xx.x%)	xx (xx.x%)
	5th ≥10 days ago	xx (xx.x%)	xx (xx.x%)	xx (xx.x%)
	6th <10 days ago	xx (xx.x%)	xx (xx.x%)	xx (xx.x%)
	6th ≥10 days ago	xx (xx.x%)	xx (xx.x%)	xx (xx.x%)
	n	xx	xx	xx
**Smoking Status**	Current	xx (xx.x%)	xx (xx.x%)	xx (xx.x%)
	Former	xx (xx.x%)	xx (xx.x%)	xx (xx.x%)
	Never	xx (xx.x%)	xx (xx.x%)	xx (xx.x%)
**Alcohol consumption (g/week)**	n	xx	xx	xx
	Mean (SD)	xx.x (xx.x)	xx.x (xx.x)	xx.x (xx.x)
	Median [IQR]	xx.x [xx, xx]	xx.x [xx, xx]	xx.x [xx, xx]
**Intercurrent diabetes**	Type 1	xx (xx.x%)	xx (xx.x%)	xx (xx.x%)
	Type 2	xx (xx.x%)	xx (xx.x%)	xx (xx.x%)
**History of Heart Disease**	Yes	xx (xx.x%)	xx (xx.x%)	xx (xx.x%)
	No	xx (xx.x%)	xx (xx.x%)	xx (xx.x%)
**History of Lung Disease**	Yes	xx (xx.x%)	xx (xx.x%)	xx (xx.x%)
	No	xx (xx.x%)	xx (xx.x%)	xx (xx.x%)
**History of Kidney Disease**	Yes	xx (xx.x%)	xx (xx.x%)	xx (xx.x%)
	No	xx (xx.x%)	xx (xx.x%)	xx (xx.x%)
**Hypertension**	Yes	xx (xx.x%)	xx (xx.x%)	xx (xx.x%)
	No	xx (xx.x%)	xx (xx.x%)	xx (xx.x%)
**Cancer**	Organ 1	xx (xx.x%)	xx (xx.x%)	xx (xx.x%)
	Organ 2	xx (xx.x%)	xx (xx.x%)	xx (xx.x%)
**Hypertension**	Organ 3	xx (xx.x%)	xx (xx.x%)	xx (xx.x%)
	Organ 4	xx (xx.x%)	xx (xx.x%)	xx (xx.x%)
**History of stroke Taking Vitamin D**	Yes	xx (xx.x%)	xx (xx.x%)	xx (xx.x%)
	No	xx (xx.x%)	xx (xx.x%)	xx (xx.x%)
	Yes	xx (xx.x%)	xx (xx.x%)	xx (xx.x%)
	No	xx (xx.x%)	xx (xx.x%)	xx (xx.x%)
**Vitamin D daily dose (IU)**	≤ 200	xx (xx.x%)	xx (xx.x%)	xx (xx.x%)
	>200 and ≤400	xx (xx.x%)	xx (xx.x%)	xx (xx.x%)
**Taking Vitamin D**	>400 and ≤1000	xx (xx.x%)	xx (xx.x%)	xx (xx.x%)
	>1000 and ≤2000	xx (xx.x%)	xx (xx.x%)	xx (xx.x%)
**Vitamin D daily dose (IU)**	>2000	xx (xx.x%)	xx (xx.x%)	xx (xx.x%)
	Yes	xx (xx.x%)	xx (xx.x%)	xx (xx.x%)

**Inhaled glucocorticoid**	No	xx (xx.x%)	xx (xx.x%)	xx (xx.x%)
**ACE inhibitor**	Yes	xx (xx.x%)	xx (xx.x%)	xx (xx.x%)
	No	xx (xx.x%)	xx (xx.x%)	xx (xx.x%)
**HMG CoA reductase inhibitor (statin)****	Yes	xx (xx.x%)	xx (xx.x%)	xx (xx.x%)
	No	xx (xx.x%)	xx (xx.x%)	xx (xx.x%)
**Proton pump inhibitor**	Yes	xx (xx.x%)	xx (xx.x%)	xx (xx.x%)
	No	xx (xx.x%)	xx (xx.x%)	xx (xx.x%)
**Aspirin*****	Yes	xx (xx.x%)	xx (xx.x%)	xx (xx.x%)
	No	xx (xx.x%)	xx (xx.x%)	xx (xx.x%)

n = count of non-missing values, SD = standard deviation, IQR = inter-quartile range
*Self-reported
^ refers to participant's most recent vaccination for Coronavirus
** Includes statin alone and in the form of combination medication with ezetimibe
*** Includes aspirin alone and aspirin in the form of a combination with another agent
Isolating data in this table refers to isolation at baseline (Day 1 of trial). I1= not isolating; I2 = in isolation with someone else who is isolating after testing positive for SARS-CoV-2; I3 = in isolation with another close contact of a case of SARS-CoV-2; I4= in isolation alone or with no-one else who has tested positive for SARS-CoV-2

If the tabulation for specific variables in table 1 proves to be very sparse, then levels for those variables will be collapsed as necessary.

Table 2: History of Heart Disease

**Heart Disease Characteristic (History of …)**	**Ivermectin**		**Placebo**	
	**Present**	**Absent**	**Present**	**Absent**
Heart Attack	nn	nn	nn	nn
Angina	nn	nn	nn	nn
Coronary Artery Bypass Surgery	nn	nn	nn	nn
Coronary Stent	nn	nn	nn	nn

Table 3: History of Lung Disease

**Lung Disease Characteristic (History of …)**	**Ivermectin**		**Placebo**	
	**Present**	**Absent**	**Present**	**Absent**
Emphysema/COPD	nn	nn	nn	nn
Asthma	nn	nn	nn	nn

Table 4: History of Kidney Disease

**Kidney Disease Characteristic (History of …)**	**Ivermectin**		**Placebo**	
	**Present**	**Absent**	**Present**	**Absent**

Dialysis	nn	nn	nn	nn
Renal Transplant	nn	nn	nn	nn

Tables 2, 3 and 4 will be provided for the subsets of participants who, according to table 1, have a history of Heart, Lung and Kidney disease respectively.

Table 5: Primary objective using the Proportion Test. The table refers to participants who developed a positive PCR or TGA-approved RAT for SARS-CoV-2

**Treatment Arm**	**n**	**Proportion who took Ivermectin**		
		**Estimate**	**95% CI**	**P-value**
**Ivermectin**	nn	xx	[x.xx, x.xx]	x.xx
**Placebo**	nn			

Proportion who took Ivermectin = Proportion of participants testing positive for SARS-CoV-2 who were given Ivermectin (as opposed to placebo). The p-value corresponds to the hypothesis test with a null hypothesis of a proportion of 0.5.

Table 6: Primary objective using Logistic Regression. The main analysis in Table 6 refers to all participants who followed protocol and were not lost to follow up in the first 14 days after treatment

**Analysis Set**	**Average Treatment Effect**	**95% CI**	**P-value**
**Main analysis**	x.xx	[x.xx, x.xx]	x.xx
**Ivermectin Dose ≥200ug/kg**	x.xx	[x.xx, x.xx]	x.xx
**Not positive for, or symptomatic of, SARS-CoV-2 within 4 hours of IP**	x.xx	[x.xx, x.xx]	x.xx

The Average Treatment Effects take the placebo group as baseline. P-values correspond to the hypothesis test with a null hypothesis of an Average Treatment Effect of 0.

Analyses for the subsets (participants with Ivermectin dose ≥200ug/kg and participants not symptomatic of, or positive for, SARS-CoV-2 within 4 hours of IP) will be provided only if their participant inclusion sets differ from that of the main analysis.

Tables 5 and 6 will be provided twice (Tables 5a and 5b, and 6a and 6b), once for the main analysis, which is to be conducted based on data available on May 31st 2024, and once for the final analysis, which is to be conducted once 14 days of follow-up post close contact are available for all participants accrued.

Table 7: Secondary objectives

Measurement	Mean			95% CI of Difference in Means	P-value
	Ivermectin	Placebo	Difference		
**DAFS at Day 14**	xx	xx	xx	[x.xx, x.xx]	x.xx
**DAFS at Day 28**	xx	xx	xx	[x.xx, x.xx]	x.xx
**DAFPH at Day 28**	xx	xx	xx	[x.xx, x.xx]	x.xx
**TTP**	xx	xx	xx	[x.xx, x.xx]	x.xx

Differences use placebo as baseline – i.e. Difference = Ivermectin minus Placebo. DAFS: Days alive and free of SARS-Cov-2 symptoms; DAFPH: days alive and free of presentation to hospital, acute hospital care and outpatient care under hospital supervision; TTP: time from exposure to an index case of SARS-CoV-2 until a positive PCR or positive TGA-approved RAT.

Table 7 will be repeated for sub-groups defined by isolation status on Day 1, where isolation status is one of: i) not isolating; ii) isolating with someone else who has tested positive to SARS-CoV-2; iii) isolating with another close contact of a case of SARS-CoV-2; iv) isolating alone or with no-one else positive. These tables will be numbered 7a, 7b, 7c and 7d.

The analysis method for the primary objective will be repeated with each of the confounders used as the independent variable in a series of univariate models.

Table 8: Univariate tests of relationships between the covariates and conversion to a positive PCR or TGA-approved RAT for SARS-CoV-2 by 14 days following exposure to an index case.

Covariate	Average Treatment Effect	95% CI	P-value
**Vaccination Status**	x.xx	[x.xx, x.xx]	x.xx
**Location of close contact**	x.xx	[x.xx, x.xx]	x.xx
**Isolation status:**	x.xx	[x.xx, x.xx]	x.xx
**Age**	x.xx	[x.xx, x.xx]	x.xx
**Prior Infection with SARS-CoV-2**	x.xx	[x.xx, x.xx]	x.xx
**Inhaled glucocorticoid**	x.xx	[x.xx, x.xx]	x.xx
**Vitamin D**	x.xx	[x.xx, x.xx]	x.xx
**ACE inhibitor**	x.xx	[x.xx, x.xx]	x.xx
**Time interval between close contact and receipt of IP**	x.xx	[x.xx, x.xx]	x.xx

Table 9: i)Univariate tests of relationships between taking oral glucocorticoid at baseline and conversion to a positive PCR or TGA-approved RAT for SARS-CoV-2 by 14 days following close contact to an index case ii)the secondary endpoints relating to symptoms (in the first 14 or 28 days post IP) or requirement for acute medical care (in the first 28 days post IP),

Outcome	**Average Treatment Effect**	**95% CI**	**P-value**
Positive PCR or RAT	x.xx	[x.xx, x.xx]	x.xx
DAFS14	x.xx	[x.xx, x.xx]	x.xx
DAFS28	x.xx	[x.xx, x.xx]	x.xx
DAFPH	x.xx	[x.xx, x.xx]	x.xx

4. References

1. Wilson EB. Probable inference, the law of succession, and statistical inference. *Journal of the American Statistical Association*. 1927;22(158):209-212. doi:10.1080/01621459.1927.10502953
2. Firth D. Bias reduction of maximum likelihood estimates. *Biometrika*. 1993;80:27-38.
3. *logistf: Firth's Bias-Reduced Logistic Regression*. 2023. https://CRAN.R-project.org/package=logistf
4. Heinze G, Schemper M. A solution to the problem of separation in logistic regression. *Statistics in Medicine*. 2002;21(16):2409-2419. doi:https://doi.org/10.1002/sim.1047
5.
6. Mood C. Logistic Regression: Why We Cannot Do What We Think We Can Do, and What We Can Do About It. *European Sociological Review*. 2010;26:67-82. doi:10.1093/esr/jcp006
7. *marginaleffects: Predictions, Comparisons, Slopes, Marginal Means, and Hypothesis*. 2024. https://CRAN.R-project.org/package=marginaleffects
8. EMA. *ICH Topic E 9 Statistical Principles for Clinical Trials*. 1998.
9. Crowder MJ. Beta-Binomial Anova for Proportions. *Journal of the Royal Statistical Society Series C (Applied Statistics)*. 1978;27(1):34-37. doi:10.2307/2346223
10. *aod: Analysis of Overdispersed Data*. 2012.
